# Proteogenomic Analysis Identifies Clinically Relevant Subgroups of Collecting Duct Carcinoma

**DOI:** 10.34133/research.0859

**Published:** 2025-09-03

**Authors:** Yuanyuan Qu, Xiaoru Pei, Jinwen Feng, Xin Yan, Linhui Zhang, Jun Wang, Xin Yao, Jiasheng Bian, Yu Gan, Hualei Gan, Xuewen Jiang, Ping Yang, Maoping Cai, Liqing Li, Xinqiang Wu, Weiwei Jing, Chao Zhang, Jianyuan Zhao, Hailiang Zhang, Guohai Shi, Xiang Zhou, Dingwei Ye, Chen Ding

**Affiliations:** ^1^ Department of Urology, Fudan University Shanghai Cancer Center, State Key Laboratory of Genetics and Development of Complex Phenotypes, School of Life Sciences, Human Phenome Institute, Fudan University, Shanghai 200433, China.; ^2^ Department of Oncology, Shanghai Medical College, Fudan University, Shanghai Genitourinary Cancer Institute, Shanghai 200032, China.; ^3^ Department of Urology, Sun Yat-sen University Cancer Center, State Key Laboratory of Oncology in South China, Guangdong Provincial Clinical Research Center for Cancer, Sun Yat-sen University Cancer Center, Guangzhou 510060, China.; ^4^ Department of Genitourinary Oncology, Tianjin Medical University Cancer Institute and Hospital, National Clinical Research Center of Cancer, Key Laboratory of Cancer Prevention and Therapy Tianjin, Tianjin’s Clinical Research Center for Cancer, Tianjin 300060, China.; ^5^ Department of Urology Surgery, Shandong Cancer Hospital and Institute, Shandong First Medical University and Shandong Academy of Medical Sciences, Jinan 250117, China.; ^6^ Tissue Bank and Department of Pathology, Fudan University Shanghai Cancer Center, Shanghai 200032, China.; ^7^ Department of Urology, Qilu Hospital of Shandong University, Jinan 250012, China.; ^8^ Department of Pathology, State Key Laboratory of Oncology in South China, Guangdong Provincial Clinical Research Center for Cancer, Sun Yat-sen University Cancer Center, Guangzhou 510060, China.; ^9^ Huzhou Central Hospital, Fifth Affiliated Clinical Medical College of Zhejiang Chinese Medical University, Huzhou 313000, China.; ^10^ Institute for Development and Regenerative Cardiovascular Medicine, MOE-Shanghai Key Laboratory of Children’s Environmental Health, Xinhua Hospital, Shanghai Jiao Tong University School of Medicine, Shanghai 200092, China.; ^11^ Department of Cancer Research Institute, Affiliated Cancer Hospital of Xinjiang Medical University, Xinjiang Key Laboratory of Translational Biomedical Engineering, Urumqi 830000, China.

## Abstract

Collecting duct carcinoma (CDC) is a rare but aggressive form of renal cell carcinoma (RCC) that has limited understanding and an undefined systemic therapeutic regimen. Herein, we conducted a comprehensive proteogenomic analysis of CDC tumors and normal adjacent tissues to elucidate the biology of the disease. CDC exhibited high heterogeneity in tumor mutational burden, and enhanced ribosome biogenesis was the most striking malignant feature of CDC, even compared with other common kidney carcinomas. Genomic data indicated that *UTP6* and *HN1* amplification on chromosome 17q were associated with the activations of ribosome biogenesis and cell migration, respectively, which were relevant to tumor proliferation and metastasis. Proteomic-based classification identified 3 clusters, among which, tumors overexpressing ribosome biogenesis signaling (GP1) clustered into the most aggressive subtype, while tumors with increased energy metabolism (GP3) exhibited significant sensitivity to anti-vascular endothelial growth factor agents. Immune subtyping revealed a complex immune landscape of CDC. Additionally, increased RPF2, contributing to ribosome production, was validated to be associated with malignant phenotypes, and targeting RPF2 could exert an anti-oncogenic role by disrupting ribosome biogenesis and perturbing the MDM2–p53 interaction.

## Introduction

Collecting duct carcinoma (CDC), also known as Bellini duct carcinoma, is a rare but aggressive form of renal cell carcinoma (RCC), accounting for 0.4% to 2.1% of all renal malignant epithelial tumors. Clinically, available series indicate that the median age at diagnosis of CDC is comparable to that of clear cell RCC (ccRCC) (63 vs. 65), while CDC demonstrates greater male predominance (70% vs. 62%) [1,2]. Patients with CDC have shorter median survival (13.2 vs. 122.5 months), higher proportions of high-grade tumors (G3+G4: 62.3% vs. 24.4%), and more advanced T-stage disease (T3+T4: 57% vs. 20.9%) compared to those with ccRCC [3]. In addition, a retrospective analysis reveals that CDC presents with more advanced TNM stage than ccRCC and exhibits higher cancer-specific mortality, even when compared to G4 ccRCC [4,5]. These results indicate that CDC shows greater clinical aggressiveness than ccRCC. Unfortunately, the underlying mechanism remains unclear.

Tumor mutational burden (TMB) is a highly studied biomarker for emerging treatment evaluation and tumor prognosis across various malignant carcinomas, including ccRCC [6]. However, the role of TMB in CDC has not yet been explored. For another, on the level of genomic aberrations, genetic studies have revealed frequent DNA deletions on chromosomal regions 8p, 16p, 1p, 9p, and 21q [7,8], and *NF2*, *SETD2*, *SMARCB1*, and *CDKN2A* were identified as the most frequently mutated genes in CDC [2,9]. Moreover, clinically relevant genomic alterations (CRGAs) of *PIK3CA*, *HRAS*, *FBXW7*, *VHL*, *DNMT3A*, and *BAP1* were also identified [2]. However, the biological mechanism connecting genome aberrations with tumorigenesis remains unclear. Furthermore, previous studies were usually limited to no more than 20 cases of CDC [2,9,10], and the alteration frequencies of frequently mutated genes often varied. Thus, more CDC samples were needed for a more comprehensive analysis.

Therapeutic options for CDC, such as immunotherapy or chemotherapy, have had limited success in controlling the disease. The majority of CDC patients unfortunately succumb to the disease within a year [11]. Oudard et al. [12] reported in metastatic CDC patients that treatment with platinum/gemcitabine, which is approved for RCC, only exhibited a median overall survival (OS) of 10.5 months and a progression-free survival (PFS) of 7.1 months. The rarity of CDC impedes the conduct of prospective randomized clinical trials, consequently making it difficult to define a systemic regimen for CDC. Therefore, there is an extremely urgent need to classify CDC through integrative analysis and propose specific therapies for different subtypes of CDC in order to improve the effectiveness of clinical treatment. Thus, to this end, in this case, we investigated the genomic, transcriptomic, proteomic, and phospho-proteomic characteristics of 53 CDC samples and correlated the findings with clinicopathological features.

## Results

### Comprehensive proteogenomic underpinnings of CDC

**Fig. 1.**
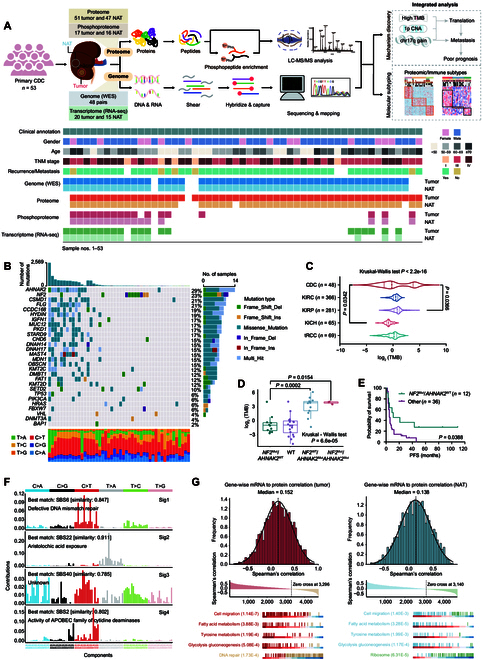
Comprehensive proteogenomic underpinnings of CDC. (A) Overview of the experimental design and the number of samples for WES, proteome, phosphoproteome, and transcriptome analyses. Clinical parameters of this CDC cohort are shown in the heatmap, including gender, age, TNM stage, recurrence, and metastasis. (B) The genomic profiles. Top panel: the number of mutations in each patient. Middle panel: somatic mutations for significantly mutated genes (SMGs) and potential SMG. Bottom panel: the distribution of 6 possible single-nucleotide substitutions in each patient. Mutation types and their frequencies are depicted by a bar plot in the right panel. (C) Violin plots illustrating the different distribution of TMB among different RCC types. Kruskal–Wallis test was used to test whether any of the differences among the RCC types were statistically significant. Wilcoxon rank-sum test was used to estimate the significance of 2 RCC types. CDC, collecting duct carcinoma; KIRC, clear cell renal cell carcinoma; KIRP, papillary renal cell carcinoma; KICH, chromophobe renal cell carcinoma; tRCC, Mit family translocation renal cell carcinoma. (D) Different distribution of TMB in patients with different genotypes of *NF2* and *AHNAK2* (Wilcoxon rank-sum test and Kruskal–Wallis test). Boxplots show the median (central line), the 25%–75% interquartile range (IQR) (box limits), AND the ±1.5×IQR (whiskers). (E) Kaplan–Meier curves of PFS for *NF2*^Mut^/*AHNAK2*^WT^ patients and other patients (2-sided log-rank test). (F) Mutational spectrum of the 4 mutational signatures extracted by Sigminer analysis. (G) Left panel: Gene-wise mRNA–protein correlation in CDC tumors. Red: pathways in which positively correlated genes were involved; yellow: pathways in which negatively correlated genes were involved. Right panel: Gene-wise mRNA–protein correlation in NATs. Blue: pathways in which positively correlated genes were involved; green: pathways in which negatively correlated genes were involved.

In total, 53 treatment-naive CDC samples from Chinese patients were profiled. All omics experiments include whole-exome sequencing (WES), RNA-sequencing (RNA-seq), proteome, and phosphoproteome. The experimental design and data availability are shown in Fig. 1A. Of the 53 CDC patients identified, 38 (72%) were male. The median age was 57 (range from 17 to 86). The ratio of male to female and the median onset age were consistent with previous reports [2,4,11]. At the time of specimen analysis, the cohort comprised predominantly advanced-stage (III/IV) patients (~91%), with only 5 stage I cases. Forty-five patients experienced recurrence or metastasis (Fig. 1A and Data S1). Moreover, compared to a previously published cohort consisting of 324 CDC patients [4], our cohort showed no significant differences in terms of gender ratio, median age, tumor size, tumor side, T stage, and M stage (Data S1), indicating that our CDC cohort is representative of the demographic and clinicopathological characteristics of CDC within the broader population.

In total, 22,929 nonsynonymous somatic mutations and 3,004 synonymous somatic mutations were identified by WES analysis. Consistent with previous studies, *NF2* and *SETD2* were the most frequently mutated genes and the mutation frequency of the 6 CRGAs (*PIK3CA*, *FBXW7*, *BAP1*, *DNMT3A*, *VHL*, and *HRAS*) was similar to that previously reported [2,9,10] (Fig. 1B, Data S2, and Fig. S1A). Alongside *NF2*, *AHNAK2* emerged as another highly mutated gene in our CDC cohort that has not been reported in other CDC studies. This might be due to either cohort size limitations or distinct mutation patterns within the ethnically Chinese population. Furthermore, we conducted a comprehensive comparison of genomic alterations between our CDC cohort and previously published CDC datasets [2,9,10], demonstrating the complex genomic variation landscape of CDC and the accuracy of the CDC diagnosis in our cohort. The detailed analyses are shown in Section SI.

TMB, quantifying cumulative genetic alterations, has demonstrated potential as a robust biomarker. Interestingly, CDC showed a relatively lower median TMB compared to the majority of tumors in The Cancer Genome Atlas (TCGA), including clear cell renal cell carcinoma (KIRC) and papillary renal cell carcinoma (KIRP) (Fig. S1B), and more than half of the CDC patients (26/48) had TMB values less than 1 (Data S2). Further compared to other RCC subtypes, CDC showed the highest TMB heterogeneity (Fig. 1C), ranging from 0.03 to 76.4 mut/Mb. Moreover, as the most frequently mutated genes, *AHNAK2* and *NF2* mutations showed different correlations with TMB, with the former being associated with higher TMB and the latter being associated with lower TMB (Fig. S1C). Interestingly, CDC patients with *NF2*^Mut^/*AHNAK2*^WT^ had the lowest median TMB value (0.51 mut/Mb) (Fig. 1D) and the best prognosis (Fig. 1E), suggesting that TMB and *NF2* mutation played a specific role in CDC and warrant further exploration.

Non-negative matrix factorization (NMF) analysis identified 4 mutational signatures (Fig. 1F and Data S2). Signatures 1 to 4 corresponded to the known COSMIC signatures: SBS6, SBS22, SBS40, and SBS2. SBS6 was correlated with defective DNA mismatch repair (MMR), while SBS22 was associated with aristolochic acid (AA) exposure, a type of carcinogen from Chinese herbs [13,14]. SBS2, commonly observed in human malignancies, has been attributed to APOBEC cytidine deaminase activity [15]. These results revealed a unique mutational signature profile of CDC.

For quality control (QC), proteomic and phosphoproteomic reproducibility was assessed via Pearson’s correlation coefficients using replicate HEK293T cell sample runs. The median inter-replicate correlation coefficient of 0.95 indicated the stability and consistency of the mass spectrometry (MS) platform (Fig. S1D). The transcriptome, proteome, and phosphoproteome of tumor (red) and normal adjacent tissues (NATs; blue) exhibited a unimodal distribution and high stability (Fig. S1E and F). A total of 12,901 protein groups were identified from all samples at a 1% false discovery rate (FDR) at the protein and peptide levels, comprising 12,161 in tumors (*n* = 51) and 11,109 in NATs (*n* = 47). Additionally, phosphoproteomic analysis identified 34,977 phosphosites corresponding to 6,864 phosphoproteins in tumor samples (*n* = 17) and 27,251 phosphosites corresponding to 5,896 phosphoproteins in NAT samples (*n* = 16). The coefficient of variation (CV) for the identified phosphosite numbers was 0.34 in tumor samples versus 0.24 in NATs, similar to previous reports in lung adenocarcinoma (LUAD) [16] and ccRCC [17], suggesting that the heterogeneity of tumor samples might cause the variable phosphoproteome identification. RNA-seq analysis identified a total of 26,472 genes, of which 13,106 genes were with fragments per kilobase of transcript per million mapped reads (FPKM) of more than 1 (Fig. S1G and H and Data S3).

To explore the relationship between the transcriptome and full proteome, we calculated gene-wise and sample-wise mRNA–protein correlations (Data S3). NATs exhibited a median gene-wise correlation of 0.138, while tumors showed an elevated median of 0.152 (Fig. 1G), similar to previous studies [18–20]. This widespread observation of low mRNA–protein correlation across multiple malignancies indicated that this phenomenon might have arisen from biological factors rather than technical ones (Supplementary Materials). Specifically, 69.05% and 66.22% of mRNA–protein pairs showed positive Spearman correlations in tumors and NATs, respectively, both of which were associated with cell migration and multiple metabolic pathways (including fatty acid, tyrosine, and glucose metabolism) (Fig. 1G). In tumors, genes with negative Spearman correlations were enriched in DNA repair; however, in NATs, genes showing negative correlations were enriched in the ribosome pathway (Fig. 1G). Sample-wise mRNA–protein correlations demonstrated a reduced median value in tumors (0.51) versus NATs (0.54), with NATs exhibiting tighter dispersion (Fig. S1I). The identified protein numbers did not affect the sample-wise mRNA–protein correlations (Fig. S1J). Additionally, the mRNA–protein correlation using log_2_ T/N (tumor/NAT) values was moderate to low with sample-wise and gene-wise median Spearman correlations of 0.31 (Fig. S1I) and 0.15 (Fig. S1K), respectively, consistent with a previous study investigating lung cancer [18]. Taken together, the distinct discordance between mRNA and protein expression indicated that proteomic data had potential to reveal unique oncogenic characters that cannot be identified by genomic or transcriptomic data alone [21].

Based on the current signatures from Lake et al. [22], we investigated the cell-type composition of CDC and common kidney cancers (including KIRC, KIRP, and chromophobe renal cell carcinoma [KICH]) from TCGA to infer the potential nephron site of origin of these malignancies. The results indicated that CDC might not only originate from medullary collecting duct epithelial cells (such as inner medullary collecting duct cells), which has been well-documented, but also be closely related to distal tubular epithelial cells (including thick ascending limb cells and distal convoluted tubule cells) (Fig. S1L and Data S1). The detailed description is shown in Section SII.

### TMB and *NF2* mutations are associated with ribosome biogenesis in CDC

**Fig. 2.**
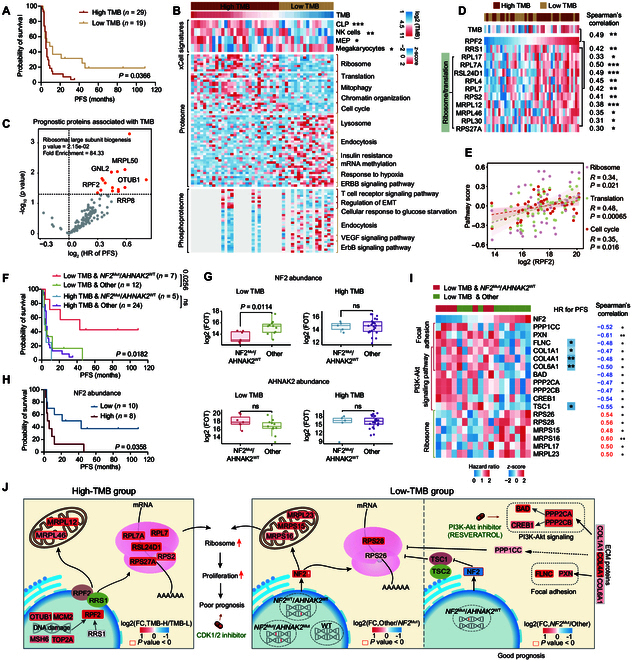
TMB and *NF2* mutations are associated with ribosome biogenesis in CDC. (A) Kaplan–Meier curves of PFS for patients with distinct TMB scores (2-sided log-rank test). (B) Heatmap illustrating different immune signatures (top panel), as well as differentially expressed proteins (middle panel) and phosphosites (bottom panel) in the high- and low-TMB groups (Wilcoxon rank-sum test) and their associated biological pathways. (C) Volcano plot showing prognostic proteins associated with TMB. Proteins with HR for PFS > 1 and *P* value < 0.05 were enriched in ribosome large subunit biogenesis. (D) Heatmap showing the abundance of ribosome proteins (RPs) significantly positive correlated with RPF2 protein abundance. The asterisks represent the statistical *P* values (*P < 0.05; **P < 0.01; ***P < 0.001). (E) Scatter plots depicting the significantly positive correlations between RPF2 protein abundance and biological pathways, including ribosome, translation, and cell cycle pathways (Spearman’s correlation test). (F) Kaplan–Meier curves of PFS for patients with different genotypes of *NF2* and *AHNAK2* combined with different TMB groups (2-sided log-rank test). (G) Boxplots showing protein abundances of NF2 and AHNAK2 in patients with different genotypes of *NF2* and *AHNAK2* combined with different TMB groups (Wilcoxon rank-sum test). (H) Kaplan–Meier curves of PFS for patients with different NF2 protein abundance in the low-TMB group (2-sided log-rank test). (I) Heatmap showing that proteins enriched in focal adhesion and PI3K-Akt signaling pathway were negatively correlated with NF2 protein abundance, while multiple RPs were positively correlated with NF2 protein abundance. Cox regression and Spearman’s correlation analyses of those protein abundances are shown on the right. Proteins with HR for PFS < 1 and *P* value <0.05 were marked. The asterisks represent the statistical *P* values (*P < 0.05; **P < 0.01). (J) A model depicting the functional impacts of TMB and genotypes of *NF2* and *AHNAK2* in CDCs.

Due to the particularly high heterogeneity of TMB (Fig. 1C), we further investigated the impacts of TMB on the clinical outcomes and proteome profiles in CDC. As previously reported, the optimal cutoff value varies depending on the TMB distribution of the different tumor types and needs to be defined dynamically, especially for rare cancers [23,24]. Herein, based on the low median value of TMB (Fig. S1A) and the ability to distinguish the PFS of CDC patients (Fig. 2A), 0.6 mut/Mb was considered as a cutoff for downstream analysis. The detailed rationale for this selection is shown in Section SIII. Twenty-nine out of 48 patients were assigned to the high-TMB group with significantly shorter PFS (Fig. 2A). The comparison analysis revealed that patients in the high-TMB group were characterized by infiltration of progenitor cells (including CLP and MEP) and NK cells, along with activation of ribosome, translation, chromatin organization, and cell cycle pathways (Fig. 2B). Further screening of prognostic proteins related to TMB revealed that OTUB1 abundance was significantly associated with worse prognosis (Fig. 2C, hazard ratio [HR] = 1.50, *P* value = 0.0299). OTUB1 has been reported to play a key non-catalytic role in DNA damage. In this cohort, the associations between OTUB1 abundance and SBS6, representing defective MMR, as well as DNA damage signal confirmed this conclusion (Fig. S2A and B), further indicating the conjectural association between DNA damage and cumulative genetic alterations in CDC.

Apart from OTUB1, pathway analysis revealed that the 15 prognostic proteins were significantly enriched in ribosome large subunit biogenesis (such as RPF2, RRP8, GNL2, and MRPL50) (Fig. 2C and Data S2), which aligned with the activation of ribosome biogenesis in the high-TMB group (Fig. 2B). Among them, RPF2, Ribosome Production Factor 2 Homolog, exhibited consistently greater fold change (FC) between high- and low-TMB groups and higher correlation coefficient with TMB, leading to its selection for downstream analyses (Data S2). RPF2 has been reported to interact with RRS1 to mediate ribosome production. In this cohort, RPF2 abundance was positively associated with the abundance of RRS1 and multiple ribosome-related proteins (RRPs) (Fig. 2D), but negatively correlated with both OS and PFS (Fig. S2C). Enhanced ribosome biogenesis and the following translation are seen as fundamental steps in tumor proliferation. Consistently, correlation analysis uncovered the positive associations between RPF2 and biological pathways, including ribosome, translation, and cell cycle (Fig. 2E). Kinase–substrate enrichment analysis (KSEA) further illustrated the activation of CDK1/2, which played a central role in cell cycle regulation, in the high-TMB group (Fig. S2D), hinting at abnormal up-regulation of tumor proliferation and therapeutic potential of CDK inhibitors. In conclusion, patients with greater TMB value showed high expression of ribosome signals, which was associated with abnormal tumor proliferation and poor prognosis (Fig. 2J).

Patients with *NF2*^Mut^/*AHNAK2*^WT^ have been found to be associated with lower TMB (Fig. 1D) and better PFS (Fig. 1F). We then explored whether this alteration had a difference in impact between the high-TMB and low-TMB groups, and a significant carcinostasis was only observed in the low-TMB group (Fig. 2F), alongside the prominent down-regulation of NF2 protein abundance (Fig. 2G). However, this alteration made no difference to AHNAK2 protein abundance regardless of TMB levels (Fig. 2G). In the low-TMB group, elevated NF2 expression was associated with adverse prognosis (Fig. 2H). Multiple RRPs were up-regulated along with the increase of NF2 abundance, whereas proteins related to focal adhesion and PI3K-Akt signaling were down-regulated as previously reported [25], including TSC1, a negative regulator of translation process [26,27] (Fig. 2I and Fig. S2E and F). PRKD1 was activated in patients with *NF2*^Mut^/*AHNAK2*^WT^ in the low-TMB group (Fig. S2G). Resveratrol, a Food and Drug Administration (FDA)-approved drug targeting PRKD1 and inhibiting PI3K-Akt signaling, is a potential treatment for this subgroup of CDC (Fig. 2J). Overall, in the low-TMB group, we reported 2 subgroups with different prognosis and ribosome signals, in which patients with *NF2*^Mut^/*AHNAK2*^WT^exhibited the decrease of ribosome signals and were associated with favorable outcomes (Fig. 2J).

### Loss of 1p is associated with improved outcome by regulating ribosome biogenesis in CDC

**Fig. 3.**
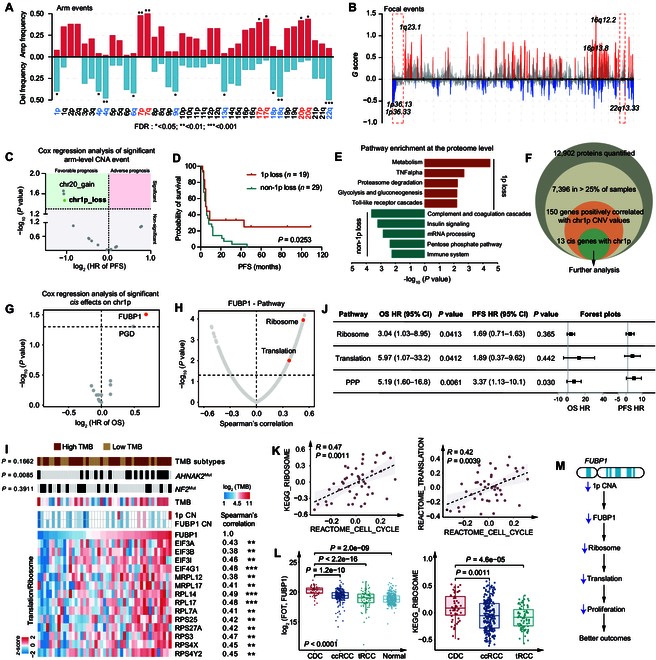
Loss of 1p is associated with improved outcome by regulating ribosome biogenesis in CDC. (A) Arm-level SCNA events. Red denotes amplification and blue denotes deletion. The asterisks represent the statistical *P* values (*P < 0.05; **P < 0.01; ***P < 0.001). (B) Focal SCNA events. Focal peaks with significant CN amplifications (red) and deletions (blue) (*q* < 0.05) are shown. (C) Cox regression analysis of significant arm-level SCNA events. The green region denotes SCNA events significantly associated with favorable prognosis and the red region denotes SCNA events significantly related to adverse prognosis. (D) Kaplan–Meier curves of PFS for patients with or without 1p loss (2-sided log-rank test). (E) Pathway enrichment analysis using DEPs for patients with 1p loss compared to patients without 1p loss. (F) The pipeline for screening significant *cis*-effects of 1p in CDC. (G) Volcano plot showing log_2_-based HR (OS) for significant positive *cis*-effect genes on 1p. (H) Volcano plot showing the Spearman’s correlation between enriched pathway scores (GSVA) and FUBP1 protein abundance. GSVA, gene set variation analysis. (I) Heatmap of FUBP1 protein abundance and *trans*-effect ribosome-translation-related proteins (Spearman’s correlation test). The asterisks represent the statistical *P* values (**P < 0.01; ***P < 0.001). The associations between FUBP1 protein abundance and genetic features are shown in the top panel (Wilcoxon rank-sum test). (J) Forest plots showing the OS and PFS HRs for selected pathways, including ribosome, translation, and pentose phosphate pathway. (K) Ribosome (KEGG) and translation (Reactome) pathways show significantly positive correlations with cell cycle (Reactome) pathway (Spearman’s correlation test). KEGG, Kyoto Encyclopedia of Genes and Genomes. (L) Left panel: Boxplots illustrating FUBP1 protein abundance among CDC, ccRCC, tRCC, and NATs. Right panel: Boxplots showing ribosome (KEGG) pathway scores among CDC, ccRCC, and tRCC. Kruskal–Wallis test was used to test whether any of the differences among the groups were statistically significant. Wilcoxon rank-sum test was used to estimate the significance of 2 groups. (M) A model depicting the functional impacts of 1p loss in CDC.

Somatic copy number alterations (SCNAs) were detected using GISTIC 2.0 [28]. Recurrent CDC DNA losses occurred at 1p, 4p, 4q, 6q, 9q, 13q, 18p, 18q, and 22q, and gains were observed in 7p, 7q, 17p, 17q, 20p, and 20q (Fig. 3A and Data S2). As shown in Fig. 3B, the most frequently deleted focal regions were found in cytobands on chromosome 1p (1p36.13, 1p36.33, etc.), consistent with a previous study investigating 29 CDC samples [8].

Since (a) chromosome 1p exhibited the highest frequency of focal deletion (Fig. 3B) and (b) 1p loss was correlated to favorable prognosis (Fig. 3C and D and Data S2), the proteome consequences of chromosome 1p loss were then systematically characterized. Pathway analysis suggested that gene sets related to metabolism, tumor necrosis factor-alpha (TNF-α), proteasome degradation, and Toll-like receptor cascades were enriched in 1p loss tumors, while complement and coagulation cascades, insulin signaling, mRNA processing, and immune system were activated in non-1p loss tumors (Fig. 3E). Interestingly, we found that glycolysis was enhanced in 1p loss tumors, while the pentose phosphate pathway (PPP) was enriched in non-1p loss tumors (Fig. 3E), suggesting distinct glucose metabolic preferences between the 2 groups. Further association analysis linking glucose metabolic pathways to clinical outcomes of patients revealed that up-regulation of both glycolysis/gluconeogenesis and PPP correlated with inferior OS and PFS (Fig. S2H and I).

Subsequently, the proteomic consequences of 1p loss were investigated. At the proteome level, high expression of 2 (*FUBP1* and *PGD*) out of 13 *cis*-effect genes was significantly correlated with poor survival (Fig. 3F and G and Fig. S2J). Phospho-gluconate dehydrogenase (PGD) is a key rate-limiting enzyme in PPP, further indicating the association between the high level of PPP and adverse outcomes. Notably, FUBP1 was the protein most associated with OS (Fig. 3G) in this cohort, which has been reported as a major regulator of transcription, translation, and RNA splicing, and has also been implicated as an oncoprotein overexpressed in multiple malignancies, including hepatocellular carcinoma (HCC), non-small cell lung cancer, breast cancer, and ccRCC [29]. Correlation analysis revealed that ribosome and translation signals had strong positive correlation with FUBP1 abundance at the proteome level (Fig. 3H). Detailed relevant proteins are shown in Fig. 3I. In addition, the activation of biological pathways, including ribosome, translation, and PPP, was a risk factor for CDC patients with poorer clinical outcomes (Fig. 3J). Moreover, significantly positive correlations were observed between cell cycle and ribosome, as well as translation signaling, indicating the enhanced tumor proliferation (Fig. 3K). Further compared to ccRCC and Mit family translocation RCC (tRCC), CDC had the significantly highest FUBP1 protein abundance and ribosome pathway score (Fig. 3L and Fig. S2K), indicating that the pharmacological targeting of FUBP1 might offer a promising therapeutic approach for CDC patients. Moreover, at the phosphoproteome level, we detected 2 phosphosites of FUBP1, namely, S630 and T629. The observed significant positive correlations between the phosphorylation levels of FUBP1 at S630 and T629 and those of translation elongation factor (EEF1B2), translation initiation factors (EIF3J and EIF4G3), and ribosomal proteins (RPS8 and RPS20) may further suggest a potentially enhanced state of intracellular translation processes (Fig. S2L and M). However, at the transcriptome level, there was no significant correlation between FUBP1 expression and pathway activity scores for ribosome or ribosome biogenesis (Fig. S2N), underscoring that proteins, as direct executors of biological function, may reveal unique oncogenic features not captured by transcriptome analysis. Collectively, we identified that 1p loss decreased FUBP1 protein expression, which was associated with down-regulation of ribosome biogenesis and translation processes, and ultimately with weakened tumor proliferation and a favorable outcome for patients with CDC (Fig. 3M).

### Enhanced ribosome biogenesis is concordant at the multi-omic level in CDC

**Fig. 4.**
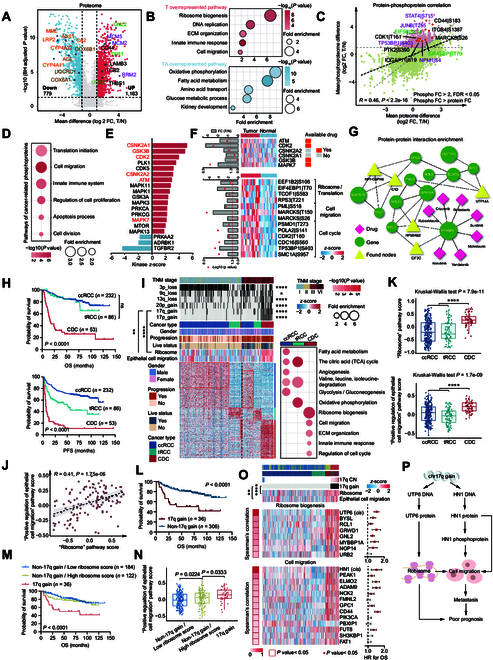
Increased ribosome biogenesis distinguishes CDC from other renal malignancies. (A) Volcano plot showing differentially expressed proteins (DEPs) in tumors and NATs (Benjamini–Hochberg-adjusted *P* value < 0.05, log_2_|FC| > 1). (B) Bubble charts showing the biological pathways enriched for the DEPs in tumors (red) and NATs (blue). (C) Comparison of abundance changes between phosphosites and their corresponding proteins. Red/pink dots: Phosphosites are greater than 2-fold changes (Benjamini–Hochberg-adjusted *P* value < 0.05) in tumors compared to NATs, and changes in phosphosite abundance are greater than changes in their corresponding protein abundance. (D) Pathways enriched in cancer-related phosphoproteins. (E) KSEA analyses of kinase activities in CDC tumors and NATs. (F) Heatmap showing the activated kinases in tumors and their corresponding substrates. The substrate-related biological pathways are shown on the right. Orange boxes indicate the existence of an FDA-approved drug. (G) Protein–protein interaction enrichment analysis for the 12 CDC candidate proteins by ProHarMeD (https://apps.cosy.bio/proharmed/). (H) Kaplan–Meier curves of OS (upper) and PFS (lower) for patients with CDC, ccRCC, and tRCC (2-sided log-rank test). (I) Left panel: Relative abundances of up-regulated proteins in the 3 RCC types (Wilcoxon rank-sum test) and associations of RCC types with clinical and genetic features (Fisher’s exact test). The asterisks represent the statistical *P* values (*P < 0.05; ****P < 0.0001). Right panel: Up-regulated pathways enriched in the 3 RCC types. (J) Scatter plot showing a significant positive correlation between ribosome pathway (GSVA) and epithelial cell migration pathway (GSVA) (Spearman’s correlation test). (K) Comparison of pathway scores for the ribosome and epithelial cell migration among the 3 RCC types. Kruskal–Wallis test was used to test whether any of the differences among the RCC types were statistically significant. Wilcoxon rank-sum test was used to estimate the significance of 2 RCC types. The asterisks represent the statistical *P* values (****P < 0.0001). Boxplots show the median (central line), the 25% to 75% IQR (box limits), and the ±1.5×IQR (whiskers). (L) Kaplan–Meier curves of OS for patients with or without 17q gain (2-sided log-rank test). (M) Kaplan–Meier curves of OS for non-17q gain patients with different ribosome pathway scores versus 17q gain patients (2-sided log-rank test). (N) Comparison of epithelial cell migration pathway scores in non-17q gain patients with different ribosome pathway scores and 17q gain patients (Wilcoxon rank-sum test). Boxplots show the median (central line), the 25% to 75% IQR (box limits), and the ±1.5×IQR (whiskers). (O) Heatmap showing the *cis*- and *trans*-effects of 17q gain and their enriched biological pathways. Top panel: The significant positive correlations between 17q gain and pathway scores for the ribosome and epithelial cell migration (Spearman’s correlation test). The asterisks represent the statistical *P* values (**P < 0.01, ****P < 0.0001). Middle panel: Heatmap showing the positive correlations between UTP6 protein abundance and ribosome biogenesis-related proteins. Bottom panel: Heatmap illustrating the positive correlations between HN1 protein abundance and cell migration-related proteins. Forest plots showing the OS HR for these proteins. (P) Systematic diagram summarizing the underlying mechanism of patients with CDC harboring 17q gain.

Principal component analysis (PCA) demonstrated marked discrimination between tumors and NATs at the transcriptome, proteome, and phosphoproteome levels (Fig. S3A). Proteomic analysis identified 1,942 differentially expressed proteins between tumors and matched NATs (FC > 2 or < 0.5, BH *P* value < 0.05), with 1,163 proteins up-regulated and 779 down-regulated in tumors (Fig. 4A). Gene set enrichment analysis (GSEA) revealed that tumor-enriched proteins were associated with pathways such as ribosome biogenesis, DNA replication, extracellular matrix (ECM) organization, innate immune response, and cell migration (Fig. 4B), whereas NAT-enriched proteins were mainly involved in pathways related to biological metabolism (including the metabolism of fatty acid, amino acid, and glucose) and kidney development (Fig. 4B). The comparison analyses at the transcriptome and phosphoproteome levels were highly similar to those at the proteome level (Fig. S3B and C).

To elucidate key signal transduction pathways, we analyzed 810 phosphosites mapped to 546 phosphoproteins, where phosphoproteomic alterations exceeded corresponding protein abundance changes (Fig. 4C, Wilcoxon rank-sum test, FC > 2, FDR< 0.05). These cancer-related phosphoproteins were enriched in pathways, including translation initiation (such as EIF4EBP1_T70 and EIF5B_S214), cell migration (such as CD44_S183 and ITGB4_S1387), and cell proliferation (such as STAT4_S715 and TP53BP1_S403) (Fig. 4D). KSEA further revealed the dominant kinases activated in tumors, where ATM, CDK2, GSK3B, and MAPK7 are targets of approved inhibitors (Fig. 4E and F). The detailed kinase selection workflow is shown in Fig. S3D. Substrates of those kinases were involved in ribosome biogenesis, translation, cell migration, and cell cycle pathways (Fig. 4F).

In addition to kinase targets, deep proteogenomic characterization of tumors and NATs also offered the possibility of nominating more potential candidates. Firstly, following rigorous proteomic screening, we nominated 21 candidate biomarkers of CDC with potential diagnostic utility (Fig. S3E). Immunohistochemistry (IHC) staining data in the Human Protein Atlas (HPA) dataset were used to help eliminate nonspecific RCC markers. As a result, 4 candidates (RPF2, GNL2, NCF4, and NUP133) from these 21 candidates showed positive expression in more than 90% of CDC samples, without high or medium IHC scores in common kidney malignancies, hinting at their potential as diagnostic biomarkers. Moreover, we employed Metascape [30] and ProHarMeD [31] to perform network-based drug repurposing and 102 proteins were filtered out after supervised analysis (Materials and Methods). Pathway and process enrichment analysis identified top 4 clusters in which these proteins were enriched, among which ribosome biogenesis was the most significant cluster (Fig. S4A). Network mapping revealed that 9 of 12 ribosome biogenesis-associated seed proteins formed a connected subnetwork within the integrated protein–drug–disease interactome (Fig. 4G). Additionally, 5 connector proteins were inferred, which were also involved in ribosome biogenesis (MPHOSPH6, C1D, UTP14A, and RPS6KB2) or translation initiation (EIF3C) processes. Moreover, drug target analysis suggested that multiple agents targeting RPS6KB1 might have the potential to treat patients with CDC (Fig. 4G). In summary, although both analytical approaches identified proteins associated with ribosome signaling, the specific proteins selected differed. The initial screening identified RPF2, GNL2, NCF4, and NUP133 as potential diagnostic biomarkers for CDC, with RPF2 and GNL2 functionally linked to ribosome biogenesis, while the network-based drug repurposing analysis identified RPS6KB1 as a potential therapeutic target with multiple FDA-approved targeted drugs for CDC.

### Increased ribosome biogenesis distinguishes CDC from other renal malignancies

Patients with CDC have drastically unfavorable clinical phenotypes [1–5]. We incorporated ccRCC (*n* = 232) and tRCC (*n* = 86) cases from published studies [20,32] for comparative analysis to investigate the distinctive mechanisms associated with clinical outcomes in CDC patients. In the combined cohort, patients with CDC had the worst OS and PFS, while patients with ccRCC had significantly favorable clinical outcomes (Fig. 4H). Protein identification is shown in Fig. S4B and PCA revealed distinct separation between tumors and NATs (Fig. S4C). Over-representation analysis (ORA) based on proteomic data suggested concordant differences in the enrichment of pathways among ccRCC, tRCC, and CDC regardless of TNM stage (Fig. 4I and Section SIV). Specifically, metabolic pathways (including fatty acid, glycose, and amino acid metabolism, as well as the citric acid cycle) and angiogenesis signal were enriched in ccRCC, consistent with the well-known conclusion that ccRCC is a metabolic disease characterized by a high level of angiogenesis [32,33]. tRCC was featured by the up-regulation of oxidative phosphorylation (OXPHOS), while no significant up-regulation of OXPHOS was observed in ccRCC and CDC. CDC was characterized by the increased ribosome biogenesis, cell migration, ECM organization, innate immune response, and regulation of cell cycle. Notably, ribosome biogenesis was the top enriched pathway in CDC compared to ccRCC and tRCC, demonstrating the pivotal role of ribosome biogenesis in CDC (Fig. 4I). A previous study proposed that elevated ribosomal content in epithelial cell lineages may augment metastatic propensity [34]. We observed a significant positive correlation between the ribosome pathway and the positive regulation of epithelial cell migration (Fig. 4J and Fig. S4D), both of which showed the highest levels in CDC (Fig. 4K), indicating that enhanced ribosome biogenesis may be associated with increased metastatic potential in CDC.

Further comparison of SCNAs revealed that patients with CDC exhibited higher frequencies of 9q and 13q losses, as well as 20p, 17q, and 17p gains (Fig. 4I). As a characteristic SCNA, 3p loss significantly occurred in patients with ccRCC (Fig. 4I). Among these SCNAs, 17q gain was positively correlated with both the ribosome pathway and the positive regulation of epithelial cell migration (Fig. 4I) and was also associated with inferior OS and PFS (Fig. 4L and Fig. S4E). Additionally, the increased ribosome signal consistently contributed to adverse clinical outcomes (Fig. S4F) in the combined cohort. Based on the level of ribosome signal and the alteration status of 17q, we divided patients into 3 subgroups (17q gain [*n* = 36], non-17q gain/high ribosome score [*n* = 122], and non-17q gain/low ribosome score [*n* = 184]). Notably, patients with 17q gain had the worst clinical outcomes and the highest level of epithelial cell migration signaling (Fig. 4M and N and Fig. S4G). Conversely, patients with a low ribosome score and non-17q gain exhibited the best clinical outcomes and the lowest level of epithelial cell migration signaling (Fig. 4M and N and Fig. S4G). After screening the coding genes on 17q, *UTP6* (Spearman’s correlation coefficient = 0.29, *P* value = 4.63e−08) and *HN1* (Spearman’s correlation coefficient = 0.18, *P* value = 1.14e−03) showed significant *cis*-effects of SCNA on protein abundances (Fig. 4O and Fig. S4H). IHC staining verified the up-regulation of UTP6 and HN1 in CDC compared to ccRCC and tRCC (Fig. S4I). Previous studies have reported that UTP6 participates in pre-18S ribosomal RNA (rRNA) processing within the nucleolus, while *HN1*, also known as *JPT1*, regulates cell migration in carcinomas including breast [35] and prostate cancer [36]. Consistently, we observed that UTP6 abundance was positively correlated with levels of proteins involved in ribosome biogenesis, whereas HN1 abundance co-varied with proteins enriched in cell migration pathway (Fig. 4O). In addition, at the phosphoproteome level, HN1 S42 phosphorylation was up-regulated in CDC compared to tRCC (Fig. S4J). High level of HN1 S42 phosphorylation was associated with poor OS and PFS (Fig. S4K). Further correlation analysis revealed that the high-level phosphorylation of HN1 S42 tended to predict a high incidence of tumor metastasis, along with high-level phosphorylation of proteins related to cell migration signaling (Fig. S4L and Data S4). Up-regulation of these phosphoproteins was also associated with reduced OS or PFS (Fig. S4L). Altogether, compared to ccRCC and tRCC, the significant high-frequency occurrence of 17q gain in CDC was associated with increased ribosome biogenesis and cell migration owing to the *cis*-acting elements on *UTP6* and *HN1*, respectively (Fig. 4P).

### Proteomic subtypes of CDC and their associations with clinical treatment options

**Fig. 5.**
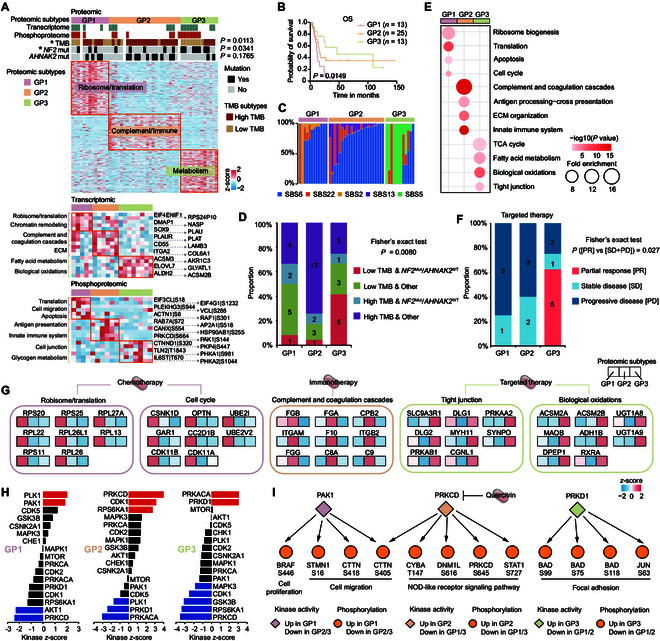
Proteomic subtypes of CDC and their associations with clinical treatment options. (A) Heatmap of differentially regulated proteins, transcripts, and phosphosites among the 3 proteomic subtypes (Wilcoxon rank-sum test, FC [T/N] >2, *P* value < 0.05), annotated with genetic features (Fisher’s exact test). (B) Kaplan–Meier curves of OS for the 3 proteomic subtypes (2-sided log-rank test). (C) Mutational signatures among the 3 proteomic subtypes. (D) Comparison of the 3 proteomic subtypes and the 4 subgroups based on genomic alterations in Fig. 2F (Fisher’s exact test). (E) Up-regulated pathways enriched in the 3 proteomic subtypes. (F) Proportions of responders (PR) and non-responders (SD/PD) to targeted (anti-VEGF) therapy among the 3 proteomic subtypes (Fisher’s exact test). PR, partial response; SD, stable disease; PD, progressive disease. (G) Proteins in 5 pathways (ribosome and translation, cell cycle, complement and coagulation cascades, tight junction, and biological oxidations) that were differentially expressed in the 3 proteomic subtypes. (H) Evaluation of kinase activities by KSEA in tumors across the 3 proteomic subtypes. (I) The kinase–substrate links of significantly activated kinases.

Due to the inter-tumoral heterogeneity, consensus clustering was conducted to identify 3 proteomic subtypes of CDC (Fig. S5A to C). These subtypes, labeled as GP1 (*n* = 13), GP2 (*n* = 25), and GP3 (*n* = 13), displayed distinct molecular and clinical features (Fig. 5A and B and Data S5). Among the 3 subtypes, GP1 showed particularly worse survival, while GP3 had relatively better survival (Fig. 5B), the highest proportion of tumor with low TMB, and the highest frequency of *NF2* mutation (Fig. 5A). The mutational signatures also varied among the 3 subtypes (Fig. 5C and Fig. S5D). Notably, 17 patients in GP2 contained SBS13 and 10 patients in GP3 contained SBS5, neither of which were identified in the overall mutational signatures (Figs. 1F and 5C). The unique mutational signature SBS2 in GP1 showed significant positive correlation with GP1-enriched pathways, including ribosome biogenesis, cell cycle, and translation imitation (Fig. S5E), suggesting a potential association between SBS2 and pro-proliferative pathways. Further comparison analysis between proteomic subtypes and genomic subgroups in Fig. 2F (Group 1: Low TMB and *NF2*^Mut^/*AHNAK2*^WT^; Group 2: Low TMB and Other; Group 3: High TMB and *NF2*^Mut^/*AHNAK2*^WT^; Group 4: High TMB and Other) revealed that GP3 showed a relatively higher overlap with Group 1, while GP2 predominantly overlapped with Group 4 (Fig. 5D).

Subtype-stratified pathway enrichment revealed distinct molecular profiles across the 3 proteomic subtypes. In total, 288, 185, and 218 proteins were found to be up-regulated in GP1, GP2, and GP3, respectively (Data S5). Proteins up-regulated in GP1 showed enrichment in pro-proliferative pathways including ribosome biogenesis, translation, apoptosis, and cell cycle. Conversely, GP2 up-regulation proteins were enriched in complement and coagulation cascades, antigen processing cross-presentation, ECM organization, and innate immune system. GP3 was more associated with enhanced metabolic pathways, including the citric acid (TCA) cycle, fatty acid metabolism, and biological oxidations (Fig. 5E). The results of pathway enrichment analysis based on the transcriptome and phosphoproteome data showed high consistency with those based on the proteome data (Fig. 5A).

The ultimate objective of protein subtyping was to guide precise clinical treatment. Due to the distinct molecular profiles among the 3 proteomic subtypes, the corresponding optimal treatment strategies also differ. Remarkably, GP3 had elevated energy and environment metabolisms, including tight junction (DLG1, DLG2, MYH11, etc.) and biological oxidations (ACSM2A, ACSM2B, UGT1A8, etc.) (Fig. 5G), which was associated with higher sensitivity to target therapy (including axitinib, pazopanib, sorafenib, and sunitinib) (Fig. 5F and Data S1). GP1 and GP2 exhibited increased tumor proliferation (CSNK1D, OPTN, CDK11B, etc.) and immune response (FGB, FGA, FGG, etc.), respectively (Fig. 5E and G), which was presumably related to sensitivity to chemotherapy (including gemcitabine plus cisplatin, gemcitabine, and gemcitabine plus oxaliplatin) and immunotherapy (including pembrolizumab and tislelizumab), respectively (Data S1). However, due to the rarity of CDC and the limited availability of patient treatment information, no significant differences were observed in response to either chemotherapy or immunotherapy among the 3 proteomic subtypes (Fig. S5F), suggesting the need for further exploration in larger cohorts.

Protein kinases are currently considered as attractive therapeutic targets in cancer treatment. Kinase activities were computationally inferred from substrate phosphorylation patterns using KSEA [37]. Comparative analysis identified distinct kinase activity profiles across the 3 proteomic subtypes, including PLK1 and PAK1 for GP1; PRKCD, CDK1, and RPS6KA1 for GP2; and PRKACA and PRKD1 for GP3 (Fig. 5H). The kinase–substrate links are shown in Fig. 5I. Quercitrin, an FDA-approved drug targeting PRKCD, is a potential treatment option for CDC in GP2.

### Characterization of immune infiltration in CDC

**Fig. 6.**
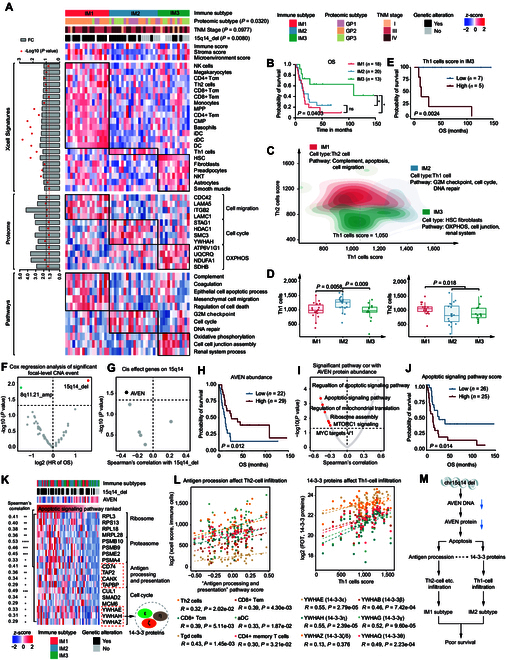
Characterization of immune infiltration in CDC. (A) The 3 immune subtypes identified by consensus clustering showing immune score, stromal score, microenvironment score, cell-type compositions, differentially regulated proteins, and single-sample GSEA (ssGSEA) pathways. Differential expression between tumors of one immune subtype versus the rest at the proteome level (Wilcoxon rank-sum test, *P* value < 0.05) and the corresponding enriched pathways (Wilcoxon rank-sum test, *P* value < 0.05) are shown. Fisher’s exact test was used to evaluate the association of immune subtypes with categorical variables, including proteomic subtypes, TNM stage, and 15q14 deletion. (B) Kaplan–Meier curves of OS for the 3 immune subtypes (2-sided log-rank test). (C) Contour plot of 2-dimensional density based on Th2 cells’ scores (*y*-axis) and Th1 cells’ scores (*x*-axis) for different immune subtypes. For each immune subtype, significant cell types and key up-regulated pathways are annotated. (D) Boxplots showing Th1 cells’ scores (left) and Th2 (right) cells’ scores among the 3 immune subtypes (Wilcoxon rank-sum test). Boxplots show the median (central line), the 25% to 75% IQR (box limits), and the ±1.5×IQR (whiskers). (E) Kaplan–Meier curves of OS for patients with different Th1 cells’ scores in IM3 (2-sided log-rank test). (F) Cox regression analysis of significant focal-level SCNA events. (G) Spearman correlation analysis showing a significant *cis*-effect gene on 15q14. (H) Kaplan–Meier curves of OS for patients with different AVEN protein abundance (2-sided log-rank test). (I) Spearman correlation analysis showing pathways correlated with AVEN protein abundance. (J) Kaplan–Meier curves of OS for patients with different apoptotic signaling pathway scores (2-sided log-rank test). (K) Heatmap showing proteins significantly positively correlated with the apoptotic signaling pathway. The corresponding enriched pathways are shown on the right. (L) Left panel: Scatter plots showing the positive associations between antigen processing and presentation (GSVA) and multiple cell-type compositions, including Th2 cells, CD8^+^ Tem, CD8^+^ Tcm, aDC, Tgd cells, and CD4^+^ memory T cells. Right panel: Scatter plots illustrating the positive correlations between Th1 cell infiltration and 14-3-3 protein abundances. Two-sided Spearman’s correlation test was used. (M) A model depicting the association between 15q14 deletion and immune signature in CDC.

To characterize the tumor microenvironment (TME) in CDC, we performed cell-type deconvolution of proteomic profiles using xCell [38]. Consensus clustering of deconvoluted cell-type proportion identified 3 immune subtypes (IM1 to IM3) showing significant association with OS (Fig. 6A and B, Fig. S6A to C, and Data S6). Interestingly, a certain degree of overlap was observed between immune and proteomic subtypes (Fig. S6D), indicating the impact of TME on protein expression profiles. Specifically, IM1 mainly overlapped with GP2, enriching CD4^+^ Tcm, CD4^+^ Tem, CD8^+^ Tcm, CD8^+^ Tem, and Th2 cells (Fig. 6A). IM2 predominantly overlapped with GP1 and GP2, with the highest Th1 cells and the lowest stromal infiltration (Fig. 6A and Fig. S6E). IM3 demonstrated a high overlap with GP3, showing higher infiltrations of stromal cells, such as fibroblasts, preadipocytes, and smooth muscle cells (Fig. 6A). Proteomic analysis further revealed that IM1 had up-regulated complement, apoptosis, and cell migration pathways, while IM2 showed increased G2M checkpoint, cell cycle, and DNA repair signals. Additionally, IM3 exhibited significant up-regulation of OXPHOS, cell junction, and renal system-related pathways (Fig. 6A and Fig. S6F). Figure 6C presents the defining characteristics of each immune subtype. Interestingly, the differential infiltrations of Th1 and Th2 cells could distinguish the 3 immune subtypes, with IM1 having the highest levels of Th2 cells and IM2 having the highest levels of Th1 cells (Fig. 6D). In IM3, higher levels of Th1 cells were associated with poorer survival (Fig. 6E). These results prompted the exploration of molecular mechanisms linking differential infiltration of Th1 and Th2 cells with distinct clinical outcomes among the 3 immune subtypes.

Remarkably, IM1 and IM2 tumors had higher frequencies of 15q14 deletion, which was the only SCNA event associated with poor survival (Fig. 6A and F). To investigate potential mechanisms linking 15q14 alterations to TME modulation and clinical outcomes in CDC, we prioritized *cis*-regulatory effects at this locus. At the proteome level, AVEN (apoptosis and caspase activation inhibitor) was the only *cis*-acting protein (Fig. 6G), whose high abundance was associated with favorable survival (Fig. 6H). AVEN has been reported as an inhibitor of apoptosis. Consistently, we observed an inverse correlation between AVEN protein abundance and apoptotic signaling (Fig. 6I), while higher apoptotic signaling contributed to poorer survival (Fig. 6J). Additionally, higher level of apoptotic signaling was correlated with higher expression of proteins involved in pathways including ribosome, proteasome, antigen processing and presentation, and cell cycle (including multiple 14-3-3 proteins) (Fig. 6K and Data S6). Correlation analysis further revealed a significantly positive correlation between antigen processing and presentation signaling and the infiltration of IM1-enriched immune cells (including Th2 cells, CD8^+^ Tem, and CD8^+^ Tcm), whereas 14-3-3 proteins (including YWHAE and YWHAH) were significant positively related to the infiltration of IM2-enriched Th1 cells (Fig. 6L). These results were consistent with the previous reports that 14-3-3 proteins could act as antigens and affect T cell polarization [39], and their overexpression was closely correlated with adverse prognosis in pancreatic, gastric, and colorectal cancer (CRC) [40]. Collectively, our results suggested that 15q14 deletion was associated with the aggregation of Th1 and Th2 cells, which were respectively related with the formation of IM2-like and IM1-like TMEs in CDC, and both these TMEs were further linked to poor survival (Fig. 6M).

### RPF2 promotes ribosome biogenesis through enhancing the catalytic activity of RNA pol I

**Fig. 7.**
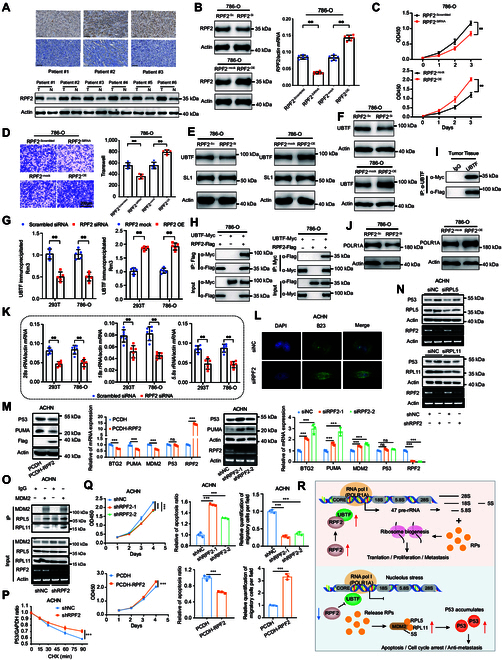
RPF2 promotes progression of CDC through ribosome production. (A) IHC (top) and WB (bottom) results of RPF2 expression in tumors and NATs. (B) Transfection efficiencies of RPF2-siRNA and RPF2 overexpression in 786-O cells are detected by WB (left) and RT-qPCR (right). (C) CCK8 assays characterize the effects of RPF2-siRNA (top) and RPF2 overexpression (bottom) on the proliferation of 786-O cells. (D) Transwell assays detect the effects of RPF2-siRNA (top) and RPF2 overexpression (bottom) on the invasiveness of 786-O cells. (E) The knockdown or overexpression of RPF2 inhibits or promotes the expression of UBTF, but has no effect on SL1 in 786-O cells. (F) Nuclear localization detection of UBTF in 786-O cells. (G) ChIP results between UBTF and Rrn3. (H and I) CO-IP assays clarify the physical interaction between RPF2 and UBTF in 786-O cells (H) and tumor tissues (I). (J) The knockdown (left) or overexpression (right) of RPF2 inhibits or promotes the expression of POLR1A in 786-O cells. (K) RPF2 knockdown inhibits the transcription of 28s, 18s, and 5.8s rRNA. (L) RPF2 depletion disrupts the nucleolar localization of NPM1 (B23). ACHN cells were transfected with control vector or RPF2 siRNA, followed by IF staining. (M) The overexpression (left) or knockdown (right) of RPF2 reduces or increases the expression of p53 and its target genes. ACHN cells were transfected with control vector, RPF2 plasmids, or RPF2 siRNAs, followed by IB and RT-qPCR analyses. (N) RPL5 or RPL11 knockdown compromises the induction of p53 by RPF2 depletion. ACHN cells were transfected with control vector, RPF2 shRNA, RPL5 siRNA, and RPL11 siRNA as indicated. Cell lysates were subjected to IB analysis with indicated antibodies. (O) RPL5–MDM2 and RPL11–MDM2 interactions are increased by depletion of RPF2. ACHN cells were transfected with RPF2 shRNA, followed by CO-IP-IB assays using antibodies as indicated. The proteasome inhibitor MG132 was supplemented into medium for 5 h before cell harvest. (P) RPF2 knockdown extends the half-life of p53 protein. ACHN cells were transfected with control vector or RPF2 shRNA. Cycloheximide (CHX) (100 mg/ml) was supplemented into medium for the indicated time before cells were harvested for IB analysis. (Q) Top panel: RPF2 knockdown suppresses proliferation and migration and promotes apoptosis of RCC cells. ACHN cells were transfected with lentivirus of control vector or RPF2 shRNAs, followed by cell viability assay, apoptosis assay, and transwell cell migration assay. Bottom panel: RPF2 overexpression promotes proliferation and migration of RCC cells. ACHN cells were transfected by lentivirus of PCDH or PCDH-RPF2 for cell viability assay, apoptosis assay, and transwell cell migration assay. The asterisks represent the statistical *P* values (*P < 0.05, **P < 0.01, ***P < 0.001). (R) A model depicting the regulation of RPF2.

In this study, we carried out supervised analysis to identify representative and robust prognostic proteins and anticipated to screen out drug targets (Fig. S3D and Fig. 4G). RPF2, involved in ribosome production, was specifically overexpressed in CDC (Fig. S3D) and significantly associated with poor clinical outcomes (Fig. S2C). IHC staining and Western blotting (WB) confirmed that RPF2 was overexpressed in CDC (Fig. 7A). As previously reported, RPF2 plays an important role in the process of ribosome biogenesis, which is not only recognized as a fundamental step in tumor proliferation, but also makes a difference in the maintenance of stem cell-like properties and epithelial–mesenchymal transition, further contributing to cancer progression. Thus, we tested whether high RPF2 expression increased tumor proliferation and migration ability. Due to the unavailability of CDC cell lines, we conducted function experiments using 4 additional kidney-derived cell lines. The results demonstrated that RPF2 overexpression profoundly promoted the proliferation and invasion of 786-O and 293T cells, while RPF2 inhibition had significantly opposite impacts (Fig. 7B to D and Fig. S7A to C).

To further investigate how RPF2 up-regulated ribosome biogenesis, we explored the relationship between RPF2 and 2 transcription factors (TFs) (SL1 and UBTF) [41] of RNA polymerase I (RNA pol I), which produces the 45S precursor of the 28s, 5.8s, and 18s rRNA components of the ribosome [42]. The results suggested that RPF2 overexpression increased UBTF protein abundance, but had no effect on SL1 (Fig. 7E and Fig. S7D). Conversely, RPF2 inhibition decreased the protein abundance of UBTF, rather than SL1 (Fig. 7E and Fig. S7D). The nuclear localization detection further revealed that RPF2 promoted the entry of UBTF into the nucleus (Fig. 7F and Fig. S7E), which is the site of ribosome biogenesis. Rrn3 is necessary for RNA pol I to effectively initiate transcription. The results of chromatin immunoprecipitation (ChIP) assay suggested that the interaction between UBTF and Rrn3 was significantly influenced by RPF2 expression (Fig. 7G). Co-immunoprecipitation (CO-IP) analysis further confirmed the physical interaction between RPF2 and UBTF at the cell line (Fig. 7H and Fig. S7F) and tumor tissue levels (Fig. 7I). Then, the expression of POLR1A, the largest and catalytic core component of RNA pol I, was detected separately under the conditions of RPF2 overexpression and inhibition to reflect the activity of RNA pol I. The results showed that RPF2 overexpression up-regulated POLR1A abundance, while RPF2 inhibition reversed this effect (Fig. 7J and Fig. S7G). Moreover, RPF2 overexpression increased the abundance of 28s, 18s, and 5.8s rRNA, whereas RPF2 inhibition decreased their abundance (Fig. 7K and Fig. S7H). Taken together, we confirmed that increased RPF2 promoted the expression and binding activity of UBTF, thereby enhancing the catalytic activity of RNA pol I and subsequent ribosome biogenesis, ultimately resulting in the development of a highly malignant phenotype (Fig. 7R).

### RPF2 deficiency plays an anti-oncogenic role via activating the RPL5/RPL11–MDM2–p53 axis

We subsequently examined the underlying mechanism by which RPF2 deficiency elicits an anti-oncogenic effect. We have revealed that RPF2 deficiency could weaken ribosome biogenesis, and disturbance of ribosome biogenesis has been reported to be related to nucleolus stress. Accordingly, we observed that depletion of RPF2 caused the release of NPM1 (B23), a nucleolar marker, from the nucleolus to the nucleoplasm in ACHN and 769-P cells by immunofluorescence (IF) staining (Fig. 7L and Fig. S7I), verifying the existence of nucleolus stress. Studies have shown that p53 activation mediates cellular response to nucleolus stress [43–45]. Consistently, we demonstrated that RPF2 overexpression or inhibition significantly reduced or enhanced the expression of p53 and its target genes, including BTG2, PUMA, and MDM2, respectively (Fig. 7M and Fig. S7J). However, the up-regulation of p53 protein caused by RPF2 depletion could be reversed by the inhibition of RPL5 and RPL11 (Fig. 7N and Fig. S7K). Additionally, we observed increased interactions between RPL5 and PRL11 with MDM2 in response to RPF2 deficiency, as demonstrated by the CO-IP assay (Fig. 7O), thus inhibiting the E3 ubiquitin ligase activity of MDM2 toward p53. Correspondingly, we found that RPF2 deficiency markedly extended the half-life of p53 protein, as quantified via the cycloheximide-chase assay (Fig. 7P and Fig. S7L). Ultimately, we found that RPF2 deficiency significantly promoted cell apoptosis and attenuated cell migration in both ACHN and 769-P cells, while RPF2 overexpression could reverse these effects (Fig. 7Q and Figs. S7M and S8A to D). Altogether, our results demonstrated that RPF2 deficiency plays an anti-oncogenic role by regulating the MDM2–p53 feedback loop in response to nucleolus stress (Fig. 7R).

## Discussion

As a rare but aggressive type of RCC, studies on CDC have mainly focused on the genomic level. The large cohort and multi-omic studies have certain limitations. To bridge the gap between genomic alterations and oncogenic protein mechanisms, a more comprehensive exploration of CDC based on proteome is needed. In this study, we present an integrative proteogenomic analysis of CDC, including genome, transcriptome, proteome, and phosphoproteome, investigating the molecular and clinical features of this disease (Fig. S8E).

TMB is a key biomarker in cancer research and has been widely studied in various malignant cancers, such as lung [46], bladder [47], and head and neck cancers [48]. Many studies have found that elevated TMB is often correlated with unfavorable prognosis and demonstrates a predictive value for immune checkpoint blockade response [49–52]. However, the role of TMB in CDC remains unclear. In our study, we confirmed the association between high TMB and poor prognosis and further considered 0.6 mut/Mb as the TMB cutoff based on (a) the low median value and (b) the high heterogeneity of TMB, as well as (c) the ability to distinguish prognosis of CDC patients. Moreover, we found the mutual exclusivity of *NF2* and the majority of frequently mutated genes, including *AHNAK2*. Further, we found that patients with *NF2*^mut^/*AHNKA2*^WT^ exhibited lower NF2 protein abundance and better prognosis in the low-TMB group, but not in the high-TMB group. Protein-based ORA revealed the association between higher TMB and activated ribosome biogenesis, while patients with *NF2*^mut^/*AHNKA2*^WT^ in the low-TMB group were characterized by increased PI3K-Akt pathway, and resveratrol targeting PRKD1 could be considered as an optional therapy for this subgroup of CDC. Intriguingly, integrated multi-omic analysis revealed that tumors harboring *AHNAK2* mutations exhibited associations with activated immune signaling and enhanced metabolic pathways. Conversely, preservation of *AHNAK2* structural integrity correlated with strengthened intercellular adhesion signaling. The detailed analyses are shown in Section SV. The recurrent *AHNAK2* alterations observed in this cohort merit further investigation into their biological implications in CDC in the future.

Mutational signatures revealed the somatic mutation patterns hidden in cancer genomes [53]. Interestingly, our analysis confirmed that the SBS6 mutational signature representing defective MMR was positively associated with TMB, but negatively associated with expression of MLH1 (Data S2), whose promoter hypermethylation has been reported to be related to the loss of MMR protein expression and higher TMB [54]. Moreover, by inferring that the patients with SBS22 were exposed to AA, we found that SBS22 was associated with higher TMB and up-regulated ribosome signaling, further indicating that AA exposure, associated with higher cumulative genetic alterations, might be conductive to the activation of ribosome signals (Data S2). The relevant analyses were presented in Sections SVI and SVII. Collectively, the integration of mutational signatures with multi-omic data provides an additional opportunity to explore the underlying biological mechanisms of CDC.

Previous studies have revealed the association between several kidney cancer subtypes and cytogenetic alterations, such as the deletion of 3p in ccRCC [33,55], trisomy of chromosome 7 or 17 in papillary RCC [56], and the deletion of 3p or 9p in tRCC [20,57]. However, the impact of CNA events in CDC is still unrevealed. Previous studies of CDC have indicated the recurrent deletions at 1p, 1q, 6p, 8p, 9p, 16p, and 21q [8,58]. In our cohort, we consistently identified significant 1p deletion, while other genetic alterations showed inconsistencies, which may be attributed to the heterogeneity of CDC, limited sample size, and population differences. Notably, in contrast to previous studies, we further investigated the relationship between 1p deletion and molecular expression patterns and suggested that 1p deletion was correlated with decreased ribosome biogenesis and the following translation, and ultimately with reduced tumor proliferation and extended OS of CDC patients by *cis*- and *trans*-effects.

Multi-omic analysis uncovered the profound differences between CDC tumors and NATs, particularly for the enhanced ribosome biogenesis in CDC. Further comparison analysis among CDC, ccRCC, and tRCC datasets indicated that ribosome biogenesis was the top enriched pathway in CDC compared to ccRCC and tRCC, which might be associated with the significant enrichment of 17q gain in CDC. Moreover, compared to ccRCC and tRCC, the ribosome biogenesis-related proteins RPF2 and GNL2 demonstrated both significantly elevated median expression levels and higher identification frequencies in CDC, supporting their potential utility as diagnostic biomarkers for CDC (Data S4). Notably, compared to CDC, the significantly up-regulated glycolysis, angiogenesis, and CA9 expression (Data S4) [59] in ccRCC, as well as significantly enhanced OXPHOS in tRCC, demonstrated the unique molecular profile of different RCC types and the diagnostic reliability of our CDC cohort. Moreover, the amplification of 2 *cis*-genes, *UTP6* and *HN1*, located at 17q, were found to activate ribosome biogenesis and cell migration in CDC, respectively, further relevant to the more malignant phenotypes, including tumor proliferation and metastasis. Therefore, it has great potential of targeting ribosome biogenesis for CDC treatment to improve the effectiveness of clinical agents.

The analysis of disease heterogeneity is the foundation of precision medicine. Our study reported 3 proteomic subtypes (GP1 to GP3) exhibiting distinct molecular profiles that stratify CDC patients by clinical outcomes. Consistent with previous studies in other tumors, translation and cell proliferation are associated with worse survival, while cellular environment and energy metabolism are related to better survival [20,32,60,61]. GP1 presented the worst survival and highest ribosome biogenesis, whereas GP3 showed the best survival, highest energy metabolism, and sensitivity to anti-vascular endothelial growth factor (VEGF) agents. The phosphoproteome data were applied in analyzing kinase features among the proteomic subtypes. PAK1 was activated in GP1, while PRKCD was activated in GP2, and PRKD1 was activated in GP3. These observations suggested that quercitrin-mediated PRKCD modulation might represent a candidate therapeutic for the GP2 subtype, where anti-VEGF options might demonstrate limited efficacy.

The tumor immune microenvironment of patients is closely linked to their clinical outcomes and therapeutic options [62–64]. Further immune subtyping of CDC tumors based on deconvolution of cell composition identified TME that described patients (IM1) who displayed higher Th2 cell infiltration from those (IM2) with higher Th1 cell infiltration. IM1 and IM2 were both associated with poor survival, while the former promoted cell migration and the latter enhanced cell cycle signal. Notably, we highlighted the specific association between 15q14 deletion and IM1- and IM2-like phenotype, which suggested the activation of the apoptotic signaling pathway. A possible explanation is that apoptosis could promote genomic instability and the creation of an onco-regenerative niche, resulting in a more aggressive phenotype [65]. In addition, we observed a significantly positive correlation between apoptosis and the recruitment of T cells, suggesting that the IM1 subtype with the richest T cell infiltration may benefit from immunotherapy. However, this conclusion needs to be further verified in a larger cohort in the future.

Our integrated analysis revealed that ribosome biogenesis was the most striking molecular feature in CDC tumors, and different bioinformatics approaches were used to filter out potential druggable candidates. Among these candidates, RPF2, involved in ribosome biogenesis, was recurrently identified through different screening methods. Previous studies have reported that RPF2 was overexpressed in several tumors, including HCC [66] and CRC [67,68]. In cultured cells, RPF2 promoted cancer cell proliferation, migration, and invasion [66,68]. Additionally, IHC staining data in the HPA dataset suggested an extremely low positive expression rate of RPF2 in common RCC types, further indicating that RPF2 might become a specific diagnostic and therapeutic target for CDC. However, the exact oncogenic mechanism of RPF2 in CDC has not been determined. In this study, we suggested that RPF2 overexpression could enhance the catalytic activity of RNA pol I via strengthening the expression and binding activity of UBTF, thereby up-regulating rRNAs production and ribosome formation, ultimately promoting tumor proliferation and invasion. Conversely, RPF2 deficiency could disrupt ribosome biogenesis and perturbed the MDM2–p53 interaction, consequently leading to impaired p53 ubiquitination, increased cell mortality and reduced cell migration. As mentioned above, due to the unavailability of CDC cell lines, these function experiments were performed using additional kidney-derived cell lines. Therefore, it is necessary to conduct these experiments again in the CDC cell line in the future.

## Conclusion

Overall, our study provides the first integrative multi-omic landscape of CDC. It reveals distinct biological insights by connecting genomic aberrations with their proteomic and phosphoproteomic consequences. Moreover, our analyses define the proteomic and immune characteristics necessary to stratify CDC patients with the aim of developing rational therapeutic strategies. We believe that the widespread use of these data will lead to biological discoveries in the future.

## Materials and Methods

### Clinical sample collection

The study adhered to the principles set forth in the World Medical Association’s Declaration of Helsinki and received approval from the institutional review board of Fudan University Shanghai Cancer Center (FUSCC; Approval No. 050432-4-1911D), Shandong Cancer Hospital and Institute (SCH; Approval No. SDTHEC-2021005017), Sun Yat-sen University Cancer Center (SYSUCC; Approval No. SL-B2021-455-01 and SL-B2022-287-01), and Tianjin Medical University Cancer Institute and Hospital (TMUCIH). Written informed consent was obtained from each patient before any study-specific investigation was conducted.

Specifically, the diagnostic procedure for CDC in our cohort included the following 3 parts.

Firstly, we strictly adhered to the 5th edition of WHO diagnostic criteria for CDC, which included (a) tumor arising in the renal medulla, (b) prominent tubular architecture, (c) desmoplastic stroma, (d) high-grade nuclear features, (e) infiltrative growth pattern, and (f) absence of other types of RCC or urothelial carcinoma components.

Secondly, before inclusion, these cases were independently reviewed by 2 senior uro-oncology pathologists from FUSCC. They conducted thorough pathological examinations and consistently diagnosed these cases as CDC.

Thirdly, representative hematoxylin–eosin (H&E) and IHC staining were conducted to present histopathological features and expression of multiple biomarkers of CDC, respectively. Generally, AE1/AE3, CK7, CK19, 34BE12, Vimentin, PAX2, PAX8, FH, and INI-1 showed positive staining, while CK20, P63, GATA3, P504S, TFE3, and CD10 exhibited negative staining, further ruling out other renal and urothelial carcinomas. The typical H&E and IHC staining images are shown in the Data S1.

Fresh frozen tissues were used in all omics experiments. Specifically, tumor and paired tumor adjacent tissues (collected >2 cm from the tumor margin) were collected within 30 min after resection, immediately transferred into sterile freezing vials, snap frozen in liquid nitrogen, cut into ~0.5-cm^3^ pieces under −40 °C, then split and stored at −80 °C until being used.

In summary, tumor and NAT samples were obtained from 53 eligible CDC patients, who underwent nephrectomy at the Urology Departments of FUSCC, SYSUCC, TMUCIH, and SCH. The median follow-up time was 15 months (range, 1.0 to 146.0 months). The cohort consisted of 71.7% (*n* = 38) males and 28.3% (*n* = 15) females, with a median age of 57 years. Among the patients, 5 patients (9.4%) had stage I tumors, while 48 patients (90.6%) had stage III/IV tumors. The detailed clinical characteristics of patients with CDC are shown in the Data S1.

### Therapeutic efficacy assessment

#### Baseline measurements

A maximum of 5 measurable target lesions (with no more than 2 per organ) were selected. Their longest diameters (LDs) were documented via contrast-enhanced CT or MRI, while lymph nodes were assessed based on short-axis measurements. The sum of the longest diameters (SLD) served as the reference value for subsequent comparisons.

#### Response criteria

Complete response (CR): Total disappearance of all target and non-target lesions, with any pathological lymph nodes shrinking to <10 mm in the short axis and no evidence of new lesions. Partial response (PR): A ≥30% reduction in SLD compared to baseline, with no new lesions or worsening of non-target disease. Stable disease (SD): Insufficient change to qualify as PR or PD (i.e., SLD reduction <30% and growth <20%), along with absence of new lesions. Progressive disease (PD): ≥20% increase in SLD from the lowest recorded value (nadir), with a minimum absolute increase of 5 mm, or emergence of any new metastatic lesions.

#### Assessment schedule and review process

Imaging studies were performed at 3-month intervals. Two certified radiologists independently analyzed the results, with any discrepancies resolved through consensus discussion.

### Whole-exome sequencing

#### DNA extraction and qualification

DNA from tumor and NAT samples was extracted using a QIAamp DNA Mini Kit (QIAGEN, Hilden, Germany) according to the manufacturer’s instructions. The concentration and integrity of DNA were detected using a Qubit 2.0 Fluorimeter (Invitrogen, CA, USA) and 1% agarose gels, respectively.

#### WES library preparation

The DNA sequencing libraries were generated using an Agilent SureSelect Human All Exon kit (Agilent Technologies, CA, USA) following the manufacturer’s recommendations. Index codes were then added to each sample. Briefly, the processes involved fragmentation of the DNA into 180- to 280-bp fragments using a hydrodynamic shearing system (Covaris, Massachusetts, USA). The remaining overhangs were converted into blunt ends via exonuclease/polymerase activities. Next, the 3′ ends of DNA fragments were adenylated, and adapter oligonucleotides were ligated. DNA fragments with adapter molecules at both ends were selectively enriched using a PCR reaction. The libraries were then hybridized with a biotin-labeled probe in the liquid phase and captured using magnetic beads with streptomycin to capture the exons of genes. Subsequently, the captured libraries underwent a PCR reaction to add index tags for sequencing preparation. Finally, the products were purified using an AMPure XP system (Beckman Coulter, Beverly, USA) and quantified using the Agilent high-sensitivity DNA assay on the Agilent Bioanalyzer 2100 system.

#### Clustering and sequencing

The clustering of the index-coded samples was performed on a cBot Cluster Generation System using a HiSeq PE Cluster Kit (Illumina) according to the manufacturer’s instructions. Subsequently, the DNA libraries were sequenced on an Illumina HiSeq platform, resulting in the generation of 150-bp paired-end reads.

### WES data analysis

#### WES data quantification and somatic mutation calling

The original fluorescence image files were converted into short reads (raw data) through base calling and recorded in FASTQ format. After the essential QC, valid sequencing data were mapped to the reference human genome (UCSC hg19) using the Burrows–Wheeler Aligner software [69]. The original mapping results were stored in binary alignment/map (BAM) format. The BAM files were then sorted and processed using SAMtools [70] and Picard (http://broadinstitute.github.io/picard/). After duplicate removal and base quality score recalibration, variant calling was conducted following Genome Analysis Toolkit (GATK) best practices. Somatic single-nucleotide variations and small insertions and deletions were detected using MuTect2 (GATK v4.1.2.0) and were annotated using ANNOVAR [71] based on known genes in UCSC refGene. The Maftools R package [72] was utilized to display mutant genes with non-synonymous mutations.

#### SCNAs calling

SCNAs were called using the CalculateTargetCoverage function in GATK (v4.1.2.0), following SCNA best practice. Then, Genomic Identification of Significant Targets in Cancer (GISTIC2.0) [28] was applied to identify significantly amplified or deleted focal-level and arm-level events. A *q* value < 0.05 was considered significant. The following parameters were used: amplification threshold = 0.1; deletion threshold = 0.1; cap value = 2.0; broad length cutoff = 0.90; remove X-chromosome = 0; confidence level = 0.95; join segment size = 4; arm-level peel off = 1; maximum sample segments = 2,000; gene GISTIC = 1. A threshold (0.1) copy number, representing the magnitude of deletion or amplification, was assigned to each gene in each sample.

#### Mutational signature analysis

Single base substitutions (SBSs) refer to the replacement of a certain nucleotide base. There are 6 possible substitutions: C>A, C>G, C>T, T>A, T>C, and T>G. Together with 5′- and 3′-flanking nucleotides, it could be stratified into 96 base substitutions in trinucleotide sequence contexts. The NMF algorithm of SigProfiler [73] was employed to identify the minimal components that could explain the maximum variance among the samples. Then, each component was compared to mutation patterns of validated cancer signatures from the COSMIC [74] database and cosine similarity was calculated to identify the best match.

#### Tumor mutational burden

TMB was defined as the number of somatic, coding, base substitution, and indel mutations per megabase of the examined genome. All base substitutions and indels in the coding region of targeted genes, including synonymous alterations, were also included to reduce sampling noise, as previously described [75]. Non-coding alterations were not taken into account. TMB was calculated by dividing the total number of counted mutations by the size of the coding sequence region.

### RNA-sequencing

#### RNA extraction, library preparation, and sequencing

Total RNA from each tissue sample was isolated using TRIzol Reagent (Invitrogen). The purity and integrity of the RNA samples were assessed. Once qualified, the RNA was reverse-transcribed into cDNA and used to construct libraries for sequencing. Clustering of the index-coded samples was performed on a cBot Cluster Generation System using the TruSeq PE Cluster Kit v3-cBot-HS (Illumina), in accordance with the manufacturer’s instructions. The libraries were then sequenced on an Illumina HiSeq 4000 platform, generating 125-bp paired-end reads.

### RNA-seq data analysis

RNA-seq raw data quality was assessed using FastQC (v0.11.9), and the adaptor was trimmed using Trim_Galore (v0.6.6) before applying any data filtering criteria. After adapter trimming and removal of low-quality tags, sequencing reads were mapped onto the hg19 reference genome using STAR software (v2.7.7.a). The mapped reads were then assembled into transcripts or genes using StringTie software (v2.1.4) [76]. For quantification purposes, the relative abundance of transcripts or genes was measured using FPKM, a normalized metric. Transcripts with a median FPKM > 1 were retained.

### Peptide preparation for MS analysis

#### Protein extraction and trypsin digestion

Collected tumor and NAT samples (approximately 50 mg of tissue each sample) were washed 3 times with 1× phosphate buffer saline (PBS) to remove blood and debris. Subsequently, the samples were minced and lysed in lysis buffer (8 M urea and 100 mM Tris hydrochloride, pH 8.0) containing protease and phosphatase inhibitors (Thermo Fisher Scientific). The samples were then sonicated for 1 min (3 s on and 3 s off, amplitude 25%, SONICS, VCX130). The lysates were centrifuged at 14,000×*g* for 10 min and the supernatants were collected as whole-tissue extracts. Protein concentrations were determined using the Bradford protein assay (TaKaRa, T9310A). Protein (1 mg) was extracted from each sample for trypsin digestion. Extracts were then reduced with 10 mM dithiothreitol at 56 °C for 30 min and alkylated with 10 mM iodoacetamide at room temperature in the dark for 30 min. The samples were then digested with trypsin using a filter-aided sample preparation method [77]. After digestion, peptides were eluted. Each eluent was divided into 2 tubes, one of which (containing 20 μg peptides) was vacuum-dried at 60 °C (Concentrator Plus, Eppendorf) and analyzed by liquid chromatography–tandem MS (LC-MS/MS) to obtain proteomic data. The remaining one was used for phosphopeptide enrichment.

#### Phosphopeptide enrichment

Phosphopeptides were enriched using the High-Select Fe-NTA Phosphopeptide Enrichment Kit (Thermo Fisher Scientific, A32992), according to the manufacturer’s instructions. Briefly, the remaining peptides were resuspended in 200 μl of binding/wash buffer and loaded onto an equilibrated spin column with Fe-NTA resin. The samples were gently tapped to mix with the resin and then incubated at room temperature for 30 min. Afterward, the mixtures were centrifuged at 1,000×*g* for 30 s to discard the flowthrough. The resin was washed 3 times with 200 μl of binding/wash buffer and once with 200 μl of LC-MS grade water. The enriched phosphopeptides bound to the NTA resin were eluted by adding 100 μl of elution buffer and centrifuging at 1,000×*g* for 30 s for 2 times. Finally, the eluted phosphopeptides were vacuum-dried using a SpeedVac at 30 °C for subsequent MS analysis.

#### Liquid chromatography–tandem MS

Samples were analyzed using the Orbitrap Exploris 480 Mass Spectrometer (Thermo Fisher Scientific) equipped with an Easy nLC-1200 (Thermo Fisher Scientific) and a Nanoflex source (Thermo Fisher Scientific). Dried peptides were dissolved in solvent A (0.1% formic acid in water) and loaded onto a trap column (100 μm × 2 cm, home-made; particle size, 3 μm; pore size, 120 Å; SunChrom, USA). They were then separated by a home-made silica microcolumn (150 μm × 30 cm; particle size, 1.9 μm; pore size, 120 Å; SunChrom, USA) using a gradient of 4% to 100% mobile phase B (80% acetonitrile and 0.1% formic acid) at a flow rate of 600 nl/min for 150 min. LC-MS/MS-based proteomic and phosphoproteomic experiments were conducted using Field Asymmetric Ion Mobility Spectrometry (FAIMS). FAIMS voltages of −45 and −65 V were set, respectively, and other parameters were consistent and set as follows: protein quantification consisted of an MS1 scan at a resolution of 120,000 (at 400 *m*/*z*). The automatic gain control (AGC) for full MS and MS/MS was set to 3E6 and 5E4, respectively, with maximum ion injection times of 80 and 22 ms, respectively.

### MS data analysis

#### Identification of peptide and protein

MS raw files were processed using the “Firmiana” [78] (a one-stop proteomic cloud platform). The human National Center for Biotechnology Information (NCBI) RefSeq protein database (updated on 4 July 2013, 32,015 entries) was used for analysis. Mascot 2.4 (Matrix Science Inc., London, UK) was used with a maximum of 2 missed cleavages. Mass tolerances were set at 20 parts per million for the precursor and 50 millimass unit for productions. Cysteine carbamidomethylation was set as fixed modification, while N-acetylation and methionine oxidation were variable modifications. For phosphoproteomic samples, phosphorylations at Ser/Thr/Tyr were considered additional variable modifications. A target-decoy-based strategy was applied to confirm protein identification quality, ensuring an FDR lower than 1% for both peptides and proteins.

#### Quantification of proteins and phosphoproteins

The one-stop proteomic cloud platform, “Firmiana”, was used to quantify proteins. The identification results and the raw data from the mzXML files were loaded into the platform. For each identified peptide, the extracted-ion chromatogram (XIC) was extracted by searching against MS1 based on its identification information. The abundance of each peptide was estimated by calculating the area under the extracted XIC curve. To calculate protein abundance, a non-redundant peptide list was used to assemble proteins following the parsimony principle. Protein abundance was then estimated using a traditional label-free, intensity-based absolute quantification (iBAQ) algorithm. This algorithm divided protein abundance (derived from identified peptide intensities) by the number of theoretically observable peptides [78,79].

The fraction of total (FOT), a relative quantification value, was defined as a protein’s iBAQ divided by the total iBAQ of all identified proteins in one experiment, calculated as the normalized abundance of a particular protein among experiments. Finally, FOT values were multiplied by 10e5 for ease of presentation and log2 transformed if necessary.

#### Missing value imputation

Proteins and phosphosites with a missing rate of greater than 75% were excluded to ensure that each sample had enough data for imputation, which aligns with a previous report [17,20,80,81]. K-nearest neighbor imputation was utilized to impute the missing values using the R package DreamAI [82]. The reliability analysis of our data filtering method is shown in Section SVIII.

#### Batch effect analysis

The ComBat function of R package sva [83] was used to eliminate the effect of batch identity. Afterward, Dip statistic test and PCA implemented in R v. 4.1.1 were adopted to assess the batch effect with regard to the following 2 variables: batch identity and sample type (tumor and NAT). The density plots of the mRNA, proteins, and phosphosites have an expected unimodal distribution (Fig. S1E), suggesting that the samples passed the QC. The results of the PCA procedure showed that batch effects were minimal for batch identity but significant for the sample types (Fig. S3A).

#### QC of the MS data

To evaluate the stability of the instrument, the HEK293T cell (National Infrastructure Cell Line Resource) lysate was regularly measured every 2 days as the standard. The QC standard underwent digestion and analysis using the same method and conditions as CDC samples. In the statistical analysis environment R (version 4.1.1), a pairwise Pearson’s correlation coefficient was calculated for all QC runs. The results are shown in fig. S1D. The median correlation coefficient among the standards was 0.95, demonstrating the consistent stability of the MS platform.

### Multi-omic data analysis

#### Co-occurrence and mutual exclusivity analysis of mutations

The somaticInteractions function in the R package Maftools was used to assess the co-occurrence and mutual exclusivity of frequently mutated genes in CDC tumors. Paired Fisher’s exact test was used to identify significant gene pairs.

#### mRNA–protein correlation analysis

A total of 4,742 genes corresponding to mRNA and protein abundances were selected to compute gene-wise and sample-wise mRNA–protein correlations. Spearman correlation coefficient between paired mRNA expression and protein abundance was measured. As a result, the median Spearman correlation coefficient for matched genes is 0.152 in tumors and 0.138 in NATs, while the median sample-wise correlation value is 0.51 for tumors and 0.54 for NATs. Functional pathways were enriched by GSEA [84] based on the correlation-ranked list of genes.

The possible reasons of the imperfect correlations of mRNA–protein included the following 3 points: (a) the temporal and spatial differences in mRNA and protein synthesis [85], (b) the low correlation between mRNA and protein half-lives [79], and (c) post-transcriptional and post-translational regulation. Please refer to Section SIX for a detailed explanation.

#### Tumor versus NATs differential genes, proteins, and phosphosites analysis

Wilcoxon rank-sum test was used to calculate the differentially expressed genes, differentially expressed proteins (DEPs), and phosphosites between CDC tumors and NATs. Genes with |log_2_(FC)| > 1 and adjusted *P* value < 0.05 were considered up-regulated genes, while |log_2_(FC)| < 1 and adjusted *P* value < 0.05 were considered down-regulated genes. For proteomics and phosphoproteomics data, cutoff value was set the same as transcriptomics. In addition, the same approach was also used to obtain DEPs between RCC types (one RCC type versus the other 2 types).

#### Pathway enrichment analysis

DEPs were subjected to functional enrichment analyses using ConsensusPathDB [86] or DAVID [87]. Gene sets of molecular pathways from Kyoto Encyclopedia of Genes and Genomes (KEGG) [88]/Hallmark [89]/Reactome [90]/Gene Ontology (GO) [91] databases were used to compute pathways. *P* value < 0.05 was considered statistically significant. The same strategy was also applied to the analysis of RNA-seq data and phosphoproteomic data.

#### Pathway score and correlation analysis

Single-sample gene set enrichment analysis (ssGSEA) was utilized to calculate pathway scores for each sample based on proteomic data using the R package GSVA [92]. This approach converted protein expression matrices across samples into gene set enrichment matrices, thereby enabling the assessment of the enrichment levels of different pathways among different samples. Gene sets including KEGG, Reactome, Hallmark, and GO downloaded from the MSigDB (https://www.gsea-msigdb.org/gsea/downloads.jsp) were set as background. Correlations between the pathway scores and other features, including protein abundance and xCell scores, were determined using Spearman’s correlation. *P* value < 0.05 was considered statistically significant.

#### Gene set enrichment analysis

GSEA [84] was conducted using the GSEA function in the R package, ClusterProfiler. Category C2, curated gene sets from online pathway databases, publications in PubMed, and knowledge of domain experts, was set as background. *P* value < 0.05 was used as a cutoff.

#### Kinase–substrate enrichment analysis

The phosphoproteomics data were processed by NetworKIN [93] to predict kinases corresponding to the identified phosphosites. Known substrates from PhosphoSitePlus [94] and UniProt were used to generate substrate sets, which were further predicted from NetworKIN with a NetworKIN score of 5 or higher. A kinase score was assigned to each kinase based solely on the collective phosphorylation status of its substrates, which was then transformed into a *z*-score. For the kinase enrichment analysis, a significance threshold of *P* value < 0.05 was used to determine significantly enriched kinases.

#### Candidate target analysis

Candidate proteins used for pathway and process enrichment (as well as protein–protein interaction enrichment) by Metascape were selected based on the following criteria: (a) the candidates were expressed in more than 50% of tumor samples; (b) the candidates exhibited up-regulated expression in tumors in more than 50% tumor–NAT pairs; (c) the median expression of candidates in tumors was at least 1.5 times higher than that in NATs (FDR < 0.05); and (d) the high expressions of candidates were significantly negatively correlated with OS or PFS (Cox regression analysis, *P* value < 0.05).

#### Proteomic subtype identification

Consensus clustering was conducted to identify proteomic subtypes of CDC tumors using the R package ConsensusClusterPlus [95] with Spearman correlation as the distance measure. The clustering was performed with the following settings: 1,000 bootstraps, 0.8 item subsampling proportion, and one feature subsampling proportion. The top 25% of the proteins with the highest CV in tumor samples (*n* = 1,849) were selected for partitioning around medoids clustering, resulting in up to 6 groups. The consensus matrices for *k* = 2, 3, 4, 5, and 6 clusters are shown in Fig. S5A, and the consensus matrix for *k* = 3 showed distinct separation among the clusters. The cumulative distribution function (CDF) plot for each *k* value is shown in Fig. S5B. The relative change in area under the CDF curve increased by approximately 30% from 2 clusters to 3 clusters, while others exhibited no appreciable increase (Fig. S5C). Based on the evidence above, the CDC tumors were clustered into 3 subtypes.

Subtype-specific up-regulated proteins were identified based on 2 criteria: (a) detection in > 25% of samples and (b) expressed higher than in other subtypes [Wilcoxon rank-sum test, log_2_(FC) > 1, *P* value < 0.05]. Subtype-specific up-regulated proteins were further analyzed using ConsensusPathDB [86] and DAVID [87]. The DEPs for each subtype and the enriched pathways are listed in Data S5.

#### Immune subtype identification

The raw enrichment scores of 64 different cell types in CDC tumors were estimated via xCell [38] (https://comphealth.ucsf.edu/app/xcell) using proteomic profiles. All 64 cell types were used for subsequent consensus clustering, using the R package ConsensusClusterPlus. The parameters used for clustering analysis were reps = 10,000, pItem = 0.8, pFeature = 1, clusterAlg = “hc”, and distance = “spearman”. *k* = 3 was selected for further analysis (Data S6).

#### Correlation between subtypes and clinical features

To examine the correlations between subtypes and clinical features, Fisher’s exact test was conducted on categorical variables, including *NF2* mutation, *AHNAK2* mutation, TMB, 15q14_del, and TNM stage. *P* value < 0.05 was considered statistically significant.

#### Survival analysis

The Kaplan–Meier method was used for survival analysis, and the 2-sided log-rank test or the Gehan–Breslow–Wilcoxon test was used to compare patients in different subgroups. *P* value < 0.05 was considered statistically significant. The HR was computed by Cox proportional hazards regression analysis. Variates with a *P* value < 0.05 were considered to have a significant impact on prognosis.

### Functional experiments

#### Cell culture

Human HEK293T (ATCC, CRL-11268; RRID: CVCL_QW54) and ACHN (ATCC, CRL-1611; RRID: CYCL_1067) cells were cultured in Dulbecco's modified eagle medium (DMEM) (high glucose) supplemented with 10% fetal bovine serum (FBS; Invitrogen), 100 units/ml penicillin (Invitrogen), and 100 μg/ml streptomycin (Invitrogen). 786-O (ATCC, CRL-1932; RRID: CVCL_1051) and 769-P (ATCC, CRL-1933; RRID: CVCL_1050) cells were maintained in RPMI 1640 medium containing 10% FBS. Cells were incubated in 5% CO_2_ at 37 °C.

#### Antibodies and primers

The primary antibodies used in this study were as follows: anti-UBTF antibody (Santa Cruz, sc-13125), anti-SL1 antibody (Santa Cruz, sc-393600), anti-POLR1A antibody (Proteintech, 20595-1-AP), anti-RPF2 antibody (Santa Cruz, sc-81060), anti-Flag antibody (ABclonal, AE005; Sigma-Aldrich, F1804), anti-Myc antibody (ABclonal, AE070), anti-GAPDH antibody (ABclonal, AC001; Proteintech, 60004-1-Ig), anti-p53 antibody (Santa Cruz, sc-126), anti-MDM2 antibody (Abcam, ab16895), anti-RPL5 antibody (Abcam, ab86863), anti-RPL11 antibody (Abcam, ab79352), anti-B23 antibody (Santa Cruz, sc-271737), anti-PUMA antibody (Cell Signaling Technology, 12450S), anti-β-actin antibody (Proteintech, 66009-1-Ig), HRP-conjugated AffiniPure Goat Anti-Rabbit IgG (BOSTER, BA1055; Proteintech, SA00001-2), and HRP-conjugated AffiniPure Goat Anti-Mouse IgG (BOSTER, BA1051; Proteintech, SA00001-1). Proteins were visualized using the ECL chemiluminescence reagent (Yeasen).

The primers used in the experiment are listed below. RPF2 (forward primer: 5′-ACAAAACCCATGCTGATATTTGC-3′, reverse primer: 5′-AGCCAGGCGGATATTTGATACT-3′); RPF2-1 (forward primer: 5′-TCAGAGGCCCCACAGTATCA-3′, reverse primer: 5′-AAAGGTCATCCGATGCCAGG-3′); UBTF (forward primer: 5′-CAGGACCGTGCAGCATATAAAG-3′, reverse primer: 5′-GCCTCGCAGCTTGGTCAT-3′); Rrn3 (forward primer: 5′-GACCGTGTCTCAGCATGATTG-3′, reverse primer: 5′-GGTGTCGATGGTACATATCTTGC-3′); 28SrRNA (forward primer: 5′-GCCATGGTAATCCTGCTCAGTAC-3′, reverse primer: 5′-GCTCCTCAGCCAAGCACATAC-3′); 18SrRNA (forward primer: 5′-ACAAAACCCATGCTGATATTTGC-3′, reverse primer: 5′-AGCCAGGCGGATATTTGATACT-3′); 5.8SrRNA (forward primer: 5′-CACTCGGCTCGTGCGTCGAT-3′, reverse primer: 5′-CGCTCAGACAGGCGTAGCCC-3′); GAPDH (forward primer: 5′-GGAGCGAGATCCCTCCAAAAT-3′, reverse primer: 5′-GGCTGTTGTCATACTTCTCATGG-3′); PUMA (forward primer: 5′-GCCAGATTTGTGAGACAAGAGG-3′, reverse primer: 5′-CAGGCACCTAATTGGGCTC-3′); MDM2 (forward primer: 5′-GAATCATCGGACTCAGGTACATC-3′, reverse primer: 5′-TCTGTCTCACTAATTGCTCTCCT-3′); BTG2 (forward primer: 5′-ACGGGAAGGGAACCGACAT-3′, reverse primer: 5′-CAGTGGTGTTTGTAGTGCTCTG-3′); p53 (forward primer: 5′-CCCAAGCAATGGATGATTTGA-3′, reverse primer: 5′-GGCATTCTGGGAGCTTCATCT-3′); and actin (forward primer: 5′-CATGTACGTTGCTATCCAGGC-3′, reverse primer: 5′-CTCCTTAATGTCACGCACGAT-3′).

Synthetic oligos were used for siRNA-mediated silencing of RPF2 (5′-GGCGAUGAUUUCGAUGUAA-3′; 5′-CCCUCAUUGGAUCUGGUUC-3′; 5′-GCGGCCAAAUAAUCUAGUA-3′), siRPL5 (5′-GGAGGAGAUGUAUAAGAAA-3′), and siRPL11 (5′-GGAACUUCGCAUCCGCAAA-3′), and scramble siRNA was used as a control. The sequences of shRNA were shRPF2-1 (5′-GCTGGCGATGATTTCGATGTA-3′) and shRPF2-2 (5′-GCAGAACACCACGGATTGAAT-3′).

The RPF2-overexpression plasmid (pCMV-RPF2-Flag, GeneChem, China; pENTER-RPF2-Flag, Vigene Biosciences, Shandong, China) was used for RPF2 overexpression.

#### Reverse transcription and RT-qPCR

Total RNA was isolated from cultured cells and converted into cDNA using specific primers and the HiScript III cDNA synthesis kit (Vazyme, Nanjing, China). The mRNA levels were determined using RT-qPCR on a CFX96 Touch real-time PCR detection system (Bio-Rad, Hercules, CA, USA). Actin was used as an internal reference gene.

#### Cell transfection

Plasmid transfection was carried out using 2 different methods: polyethylenimine (PEI) and Lipofectamine 2000 (Invitrogen). For the PEI transfection method, 500 μl of DMEM (serum-free medium) and the plasmid were combined in an empty EP tube and PEI (3 times the concentration of plasmid) was added to the medium with vigorous shaking. The mixture was incubated for 15 min. Meanwhile, the cell culture medium was replaced with 2 ml of fresh medium. After 15 min, the mixture was added to the cells, and the fresh medium was replaced after 12 h. After 36 h, the transfection process was completed and the cells were either treated or harvested. For the Lipofectamine 2000 transfection method, DMEM (250 μl) was added to 2 clean EP tubes. Then, 6 μl of Lipofectamine 2000 was added to one of the tubes and mixed for 5 min. The plasmid was added to the other tube and then combined with the medium containing Lipofectamine 2000. The mixture was mixed and allowed to stand for 20 min. Meanwhile, the cell culture medium was replaced by serum-free medium as serum can interfere with Lipofectamine 2000 transfection efficiency. After adding the mixture to the cells for 6 h, the medium was replaced with fresh normal medium, and the cells were collected 36 h later.

#### Western blotting analysis

Cultured cells were lysed using a 0.5% NP-40 buffer containing 50 mM Tris-HCl (pH 7.5), 150 mM NaCl, 0.5% Nonidet P-40, and a cocktail of protease inhibitors (Sigma-Aldrich, St. Louis, MO, USA). After centrifugation at 16,000×*g* at 4 °C for 15 min, the lysate supernatants were analyzed using Western blotting according to standard procedures. Protein abundance was detected by measuring chemiluminescence on a Typhoon FLA 9500 instrument (GE Healthcare, Little Chalfont, UK).

#### IF staining

ACHN and 769-P cells were transfected with siRNA as indicated in the figure legends. The transfected cells were fixed with methanol at −20 °C overnight. Subsequently, the cells were washed with PBS and blocked with 8% bovine serum albumin (BSA) in PBS for 1 h. The cells were then incubated with primary antibodies (anti-NPM1, diluted in 1:100) in 2% BSA at 4 °C overnight. After that, the cells were washed with PBS and incubated with the corresponding fluorescent secondary antibodies (Yeasen) and 4′,6-diamidino-2-phenylindole (Sigma-Aldrich). Images were acquired using an inverted fluorescence microscope (Leica, Wetzlar, Germany).

#### IHC staining

IHC was performed on formalin-fixed tumor and matched NATs. First, the tissues were embedded in paraffin, and 4-micron-thick sections were made. These sections were then unfolded in a water bath at 50 °C and baked at 60 °C for 2 h. Next, the tissues were dewaxed in xylene and rehydrated using gradient ethanol and finally in water. Endogenous peroxidase activity was blocked using H_2_O_2_ and antigen retrieval was performed. Further, 5% BSA was applied to block the sections for 30 min at 37 °C. The tissues were then incubated with the primary antibody at 4 °C overnight, followed by incubation with the secondary antibody for 30 min at 37 °C on the next day. Finally, the tissues were stained using hematoxylin, dehydrated, permeabilized, and sealed. Two board-certified pathologists, blinded to subtype, each selected 5 random high-power fields per sample. Within each field, staining intensity was scored as 1 (weak), 2 (moderate), or 3 (strong), and the *H* score was calculated as: (percentage of weak cells × 1) + (percentage of moderate cells × 2) + (percentage of strong cells × 3), yielding a range of 0 to 300. Differences between scorers of less than 5% were averaged.

#### CO-IP assay

Cells were harvested and lysed using mild RIPA buffer on a plate for 30 min. Meanwhile, 50 μl of Dynabeads Protein G (Life Technologies) was incubated with 3 μg of antibody at room temperature for 1 h. Next, the protein lysate was mixed with the beads–antibody complex and incubated overnight at 4 °C. The beads were washed 3 times with lysis buffer. The bound proteins and 10% inputs were detected using WB.

#### ChIP assay

ChIP assays were conducted using an EZ ChIP kit (Upstate) according to the manufacturer’s instructions. First, cultured cells and villous samples from humans were treated with 1% formaldehyde for 10 min to crosslink the DNA. Subsequently, the DNA was sonicated to generate fragments with a mean length of 200 to 500 bp. The sheared chromatin was then immunoprecipitated overnight at 4 °C using antibodies specific to UBTF, RPF2, or a non-specific rabbit IgG (Santa Cruz). The DNA fragments that were precipitated were identified and quantified using PCR and real-time qPCR with the primers designed for Rrn3.

#### Colony formation assay

Cells (1 × 10^3^) were plated in a 6-cm plate 6 to 18 h after transfection and cultured for 14 days. The medium was changed every 3 days until colonies became visible. Once visible, the colonies were fixed with methanol and stained with a 0.2% crystal violet solution at room temperature for 30 min. The count of colonies was then quantified using ImageJ.

#### Transwell assay

Before the experiment, the Matrigel matrix (BD, 356234) was melted overnight at 4 °C. The pipette tips were pre-cooled on ice for 30 min, and the Matrigel matrix was diluted to a working concentration of 300 μg/ml with pre-cooled serum-free medium. Then, 100 μl of the diluted matrix was added to each chamber (Corning, 3422) nested with a transwell-24 membrane and incubated in an incubator at 37 °C for 1 h to form a basement membrane simulating the body at the bottom of the chamber. The pre-treated cells were digested with trypsin, resuspended with serum-free medium, counted, and concentrated to 2.5×10^5^ cells/ml. Next, the Transwell chamber with the formed basement membrane was taken out, and the residual liquid in the chamber was removed. Then, 600 μl of medium containing 20% FBS was added to the lower chamber, and 100 μl of cell suspension with different pretreatment was added to the upper chamber. The chamber was incubated in a cell incubator at 37 °C and 5% CO_2_ for 24 h. After incubation, the chamber was taken out and washed 3 times with PBS. Each chamber was fixed with 600 μl of 100% methanol and left to stand at room temperature for 30 min. Subsequently, each chamber was stained with 600 μl of 0.1% crystal violet (Solarbio, G1063) and left to stand at room temperature for 20 min. Gently rubbing with a cotton ball was performed to remove non-invasive cells from the upper compartment. Finally, the cells were observed in random 5 fields under a 100× microscope, counted, and analyzed.

#### CCK-8 assay

Dispense 100 μl of a prepared cell suspension (5,000 cells/well) into a 96-well plate. Incubate the plate for an appropriate period (24, 48, or 72 h) in an incubator. Next, add 10 μl of CCK-8 solution to each well, taking care not to introduce any bubbles that may interfere with the OD reading. Incubate the plate for 2 h in the incubator. Finally, measure the absorbance at 450 nm using a microplate reader.

#### Apoptosis analysis

Apoptosis was examined using the Annexin-V-phycoerythrin (PE)/7-aminoactinomycin D (7-AAD) kit (Vazyme) and flow cytometry. Briefly, cells were harvested at 48 h after transfection and then stained with Annexin-V-PE and 7-AAD for 20 min at room temperature in the dark. The flow cytometry analysis was performed using a Beckman Coulter instrument to measure the percentage of apoptotic cells.

### Statistical analysis

Quantification methods and statistical analysis methods for proteomic and integrated analyses were mainly described and referenced in the respective subsections of Materials and Methods.

Additionally, standard statistical tests were used to analyze the clinical data, including but not limited to the Wilcoxon rank-sum test, Fisher’s exact test, Kruskal–Wallis test, Spearman’s correlation test, and log-rank test. Statistical significance was considered when the *P* value < 0.05. To address the issue of multiple testing, the *P* values were adjusted using the Benjamini–Hochberg FDR correction. Kaplan–Meier plots (2-sided log-rank test or Gehan–Breslow–Wilcoxon test) were used to describe survival rates. Variables associated with OS and PFS were identified using univariate Cox proportional hazards regression models. All analyses of the clinical data were performed using R (version 4.1.1) and GraphPad Prism (version 9.5.0).

## Data Availability

The proteome and phosphoproteome raw datasets generalized in this study have been deposited to the ProteomeXchange Consortium (dataset identifier: PXD056385) via the iProX partner repository (https://www.iprox.cn/) [96] under Project ID: IPX0008041000. No special code was used in this study, and code for all figures in the study are available for research purposes from the corresponding authors upon request.

## References

[1] Wright JL, Risk MC, Hotaling J, Lin DW. Effect of collecting duct histology on renal cell cancer outcome. J Urol. 2009;182(6):2595–2599. 19836761 10.1016/j.juro.2009.08.049 PMC2828767

[2] Pal SK, Choueiri TK, Wang K, Khaira D, Karam JA, Van Allen E, Palma NA, Stein MN, Johnson A, Squillace R, et al. Characterization of clinical cases of collecting duct carcinoma of the kidney assessed by comprehensive genomic profiling. Eur Urol. 2016;70(3):516–521. 26149668 10.1016/j.eururo.2015.06.019

[3] Sui W, Matulay JT, Robins DJ, James MB, Onyeji IC, RoyChoudhury A, Wenske S, DeCastro GJ. Collecting duct carcinoma of the kidney: Disease characteristics and treatment outcomes from the National Cancer Database. Urol Oncol. 2017;35(9):540.e13–540.e18. 10.1016/j.urolonc.2017.04.010 28495554

[4] Xiao R, Liu C, He W, Ma L. Prognostic factors and a nomogram predicting overall survival and cancer-specific survival for patients with collecting duct renal cell carcinoma. Biomed Res Int. 2021;2021: Article 6736008. 34805402 10.1155/2021/6736008 PMC8601848

[5] Deuker M, Stolzenbach F, Rosiello G, Palumbo C, Martin T, Tian Z, Chun FK, Saad F, Shariat SF, Kapoor A, et al. Renal cell carcinoma: Comparison between variant histology and clear cell carcinoma across all stages and treatment modalities. J Urol. 2020;204(4):671–676. 32250728 10.1097/JU.0000000000001063

[6] Zhang C, Li Z, Qi F, Hu X, Luo J. Exploration of the relationships between tumor mutation burden with immune infiltrates in clear cell renal cell carcinoma. Ann Transl Med. 2019;7(22): Article 648. 31930049 10.21037/atm.2019.10.84 PMC6944593

[7] Pagani F, Colecchia M, Sepe P, Apollonio G, Claps M, Verzoni E, de Braud F, Procopio G. Collecting ducts carcinoma: An orphan disease. Literature overview and future perspectives. Cancer Treat Rev. 2019;79: Article 101891. 31491662 10.1016/j.ctrv.2019.101891

[8] Becker F, Junker K, Parr M, Hartmann A, Füssel S, Toma M, Grobholz R, Pflugmann T, Wullich B, Strauss A, et al. Collecting duct carcinomas represent a unique tumor entity based on genetic alterations. PLOS ONE. 2013;8(10): Article e78137. 24167600 10.1371/journal.pone.0078137 PMC3805592

[9] Wang J, Papanicolau-Sengos A, Chintala S, Wei L, Liu B, Hu Q, Miles KM, Conroy JM, Glenn ST, Costantini M, et al. Collecting duct carcinoma of the kidney is associated with CDKN2A deletion and SLC family gene up-regulation. Oncotarget. 2016;7(21):29901–29915. 27144525 10.18632/oncotarget.9093 PMC5058651

[10] Zhang H, Lu X, Huang G, Hua M, Zhang W, Wang T, Huang L, Wang Z, Chen Q, Li J, et al. A genomic mutation spectrum of collecting duct carcinoma in the Chinese population. BMC Med Genet. 2022;15(1): Article 1. 10.1186/s12920-021-01143-2 PMC8722201 34980126

[11] Karakiewicz PI, Trinh QD, Rioux-Leclercq N, de la Taille A, Novara G, Tostain J, Cindolo L, Ficarra V, Artibani W, Schips L, et al. Collecting duct renal cell carcinoma: A matched analysis of 41 cases. Eur Urol. 2007;52(4):1140–1145. 17336449 10.1016/j.eururo.2007.01.070

[12] Oudard S, Banu E, Vieillefond A, Fournier L, Priou F, Medioni J, Banu A, Duclos B, Rolland F, Escudier B, et al. Prospective multicenter phase II study of gemcitabine plus platinum salt for metastatic collecting duct carcinoma: Results of a GETUG (Groupe d’Etudes des Tumeurs Uro-Génitales) study. J Urol. 2007;177(5):1698–1702. 17437788 10.1016/j.juro.2007.01.063

[13] Wang XM, Lu Y, Song YM, Dong J, Li RY, Wang GL, Wang X, Zhang SD, Dong ZH, Lu M, et al. Integrative genomic study of Chinese clear cell renal cell carcinoma reveals features associated with thrombus. Nat Commun. 2020;11(1): Article 739. 32029730 10.1038/s41467-020-14601-9 PMC7005298

[14] Hoang ML, Chen CH, Chen PC, Roberts NJ, Dickman KG, Yun BH, Turesky RJ, Pu YS, Vogelstein B, Papadopoulos N, et al. Aristolochic acid in the etiology of renal cell carcinoma. Cancer Epidemiol Biomarkers Prev. 2016;25(12):1600–1608. 27555084 10.1158/1055-9965.EPI-16-0219 PMC5533284

[15] Petljak M, Alexandrov LB, Brammeld JS, Price S, Wedge DC, Grossmann S, Dawson KJ, Ju YS, Iorio F, Tubio JM, et al. Characterizing mutational signatures in human cancer cell lines reveals episodic APOBEC mutagenesis. Cell. 2019;176(6):1282–1294.e20. 30849372 10.1016/j.cell.2019.02.012 PMC6424819

[16] Xu JY, Zhang C, Wang X, Zhai L, Ma Y, Mao Y, Qian K, Sun C, Liu Z, Jiang S, et al. Integrative proteomic characterization of human lung adenocarcinoma. Cell. 2020;182:245–261.e17. 32649877 10.1016/j.cell.2020.05.043

[17] Zhang H, Bai L, Wu XQ, Tian X, Feng J, Wu X, Shi GH, Pei X, Lyu J, Yang G, et al. Proteogenomics of clear cell renal cell carcinoma response to tyrosine kinase inhibitor. Nat Commun. 2023;14(1): Article 4274. 37460463 10.1038/s41467-023-39981-6 PMC10352361

[18] Chen YJ, Roumeliotis TI, Chang YH, Chen CT, Han CL, Lin MH, Chen HW, Chang GC, Chang YL, Wu CT, et al. Proteogenomics of non-smoking lung cancer in East Asia delineates molecular signatures of pathogenesis and progression. Cell. 2020;182(1):226–244.e17. 32649875 10.1016/j.cell.2020.06.012

[19] Zhang B, Wang J, Wang X, Zhu J, Liu Q, Shi Z, Chambers MC, Zimmerman LJ, Shaddox KF, Kim S, et al. Proteogenomic characterization of human colon and rectal cancer. Nature. 2014;513(7518):382–387. 25043054 10.1038/nature13438 PMC4249766

[20] Qu Y, Wu X, Anwaier A, Feng J, Xu W, Pei X, Zhu Y, Liu Y, Bai L, Yang G, et al. Proteogenomic characterization of MiT family translocation renal cell carcinoma. Nat Commun. 2022;13(1): Article 7494. 36470859 10.1038/s41467-022-34460-w PMC9722939

[21] Ying W. Phenomic studies on diseases: Potential and challenges. Phenomics. 2023;3(3):285–299. 36714223 10.1007/s43657-022-00089-4 PMC9867904

[22] Lake BB, Menon R, Winfree S, Hu Q, Melo Ferreira R, Kalhor K, Barwinska D, Otto EA, Ferkowicz M, Diep D, et al. An atlas of healthy and injured cell states and niches in the human kidney. Nature. 2023;619(7970):585–594. 37468583 10.1038/s41586-023-05769-3 PMC10356613

[23] Budak B, Arga KY. Tumor mutation burden as a cornerstone in precision oncology landscapes: Effect of panel size and uncertainty in cutoffs. OMICS. 2024;28(4):193–203. 38657109 10.1089/omi.2024.0015

[24] Muquith M, Espinoza M, Elliott A, Xiu J, Seeber A, el-Deiry W, Antonarakis ES, Graff SL, Hall MJ, Borghaei H, et al. Tissue-specific thresholds of mutation burden associated with anti-PD-1/L1 therapy benefit and prognosis in microsatellite-stable cancers. Nat Cancer. 2024;5(7):1121–1129. 38528112 10.1038/s43018-024-00752-x

[25] Cooper J, Xu Q, Zhou L, Pavlovic M, Ojeda V, Moulick K, de Stanchina E, Poirier JT, Zauderer M, Rudin CM, et al. Combined inhibition of NEDD8-activating enzyme and mTOR suppresses NF2 loss-driven tumorigenesis. Mol Cancer Ther. 2017;16(8):1693–1704. 28468780 10.1158/1535-7163.MCT-16-0821 PMC5929164

[26] Pelletier CL, Maggi LB Jr, Brady SN, Scheidenhelm DK, Gutmann DH, Weber JD. TSC1 sets the rate of ribosome export and protein synthesis through nucleophosmin translation. Cancer Res. 2007;67(4):1609–1617. 17308101 10.1158/0008-5472.CAN-06-2875 PMC2859708

[27] Malone D, Lardelli RM, Li M, David M. Dephosphorylation activates the interferon-stimulated Schlafen family member 11 in the DNA damage response. J Biol Chem. 2019;294(40):14674–14685. 31395656 10.1074/jbc.RA118.006588 PMC6779438

[28] Mermel CH, Schumacher SE, Hill B, Meyerson ML, Beroukhim R, Getz G. GISTIC2.0 facilitates sensitive and confident localization of the targets of focal somatic copy-number alteration in human cancers. Genome Biol. 2011;12(4): Article R41. 21527027 10.1186/gb-2011-12-4-r41 PMC3218867

[29] Debaize L, Troadec MB. The master regulator FUBP1: Its emerging role in normal cell function and malignant development. Cell Mol Life Sci. 2019;76(2):259–281. 30343319 10.1007/s00018-018-2933-6 PMC11105487

[30] Zhou Y, Zhou B, Pache L, Chang M, Khodabakhshi AH, Tanaseichuk O, Benner C, Chanda SK. Metascape provides a biologist-oriented resource for the analysis of systems-level datasets. Nat Commun. 2019;10(1): Article 1523. 30944313 10.1038/s41467-019-09234-6 PMC6447622

[31] Adamowicz K, Arend L, Maier A, Schmidt JR, Kuster B, Tsoy O, Zolotareva O, Baumbach J, Laske T. Proteomic meta-study harmonization, mechanotyping and drug repurposing candidate prediction with ProHarMeD. npj Syst Biol Appl. 2023;9(1): Article 49. 37816770 10.1038/s41540-023-00311-7 PMC10564802

[32] Qu Y, Feng J, Wu X, Bai L, Xu W, Zhu L, Liu Y, Xu F, Zhang X, Yang G, et al. A proteogenomic analysis of clear cell renal cell carcinoma in a Chinese population. Nat Commun. 2022;13(1): Article 2052. 35440542 10.1038/s41467-022-29577-x PMC9019091

[33] Clark DJ, Dhanasekaran SM, Petralia F, Pan J, Song X, Hu Y, da Veiga Leprevost F, Reva B, Lih TS, Chang HY, et al. Integrated proteogenomic characterization of clear cell renal cell carcinoma. Cell. 2019;179(4):964–983.e31. 31675502 10.1016/j.cell.2019.10.007 PMC7331093

[34] Ebright RY, Lee S, Wittner BS, Niederhoffer KL, Nicholson BT, Bardia A, Truesdell S, Wiley DF, Wesley B, Li S, et al. Deregulation of ribosomal protein expression and translation promotes breast cancer metastasis. Science. 2020;367(6485):1468–1473. 32029688 10.1126/science.aay0939 PMC7307008

[35] Zhang ZG, Chen WX, Wu YH, Liang HF, Zhang BX. MiR-132 prohibits proliferation, invasion, migration, and metastasis in breast cancer by targeting HN1. Biochem Biophys Res Commun. 2014;454(1):109–114. 25450365 10.1016/j.bbrc.2014.10.049

[36] Varisli L, Ozturk BE, Akyuz GK, Korkmaz KS. HN1 negatively influences the beta-catenin/E-cadherin interaction, and contributes to migration in prostate cells. J Cell Biochem. 2015;116(1):170–178. 25169422 10.1002/jcb.24956

[37] Wiredja DD, Koyutürk M, Chance MR. The KSEA app: A web-based tool for kinase activity inference from quantitative phosphoproteomics. Bioinformatics. 2017;33(21):3489–3491. 28655153 10.1093/bioinformatics/btx415 PMC5860163

[38] Aran D, Hu Z, Butte AJ. xCell: Digitally portraying the tissue cellular heterogeneity landscape. Genome Biol. 2017;18(1): Article 220. 29141660 10.1186/s13059-017-1349-1 PMC5688663

[39] Chakravarti R. Immune regulations by 14-3-3: A misty terrain. Immunobiology. 2021;226(6): Article 152145. 34628289 10.1016/j.imbio.2021.152145

[40] Huang Y, Yang M, Huang W. 14-3-3 σ: A potential biomolecule for cancer therapy. Clin Chim Acta. 2020;511:50–58. 32950519 10.1016/j.cca.2020.09.009

[41] Friedrich JK, Panov KI, Cabart P, Russell J, Zomerdijk JC. TBP-TAF complex SL1 directs RNA polymerase I pre-initiation complex formation and stabilizes upstream binding factor at the rDNA promoter. J Biol Chem. 2005;280(33):29551–29558. 15970593 10.1074/jbc.M501595200 PMC3858828

[42] McStay B, Grummt I. The epigenetics of rRNA genes: From molecular to chromosome biology. Annu Rev Cell Dev Biol. 2008;24:131–157. 18616426 10.1146/annurev.cellbio.24.110707.175259

[43] Gan Y, Deng J, Hao Q, Huang Y, Han T, Xu JG, Zhao M, Yao L, Xu Y, Xiong J, et al. UTP11 deficiency suppresses cancer development via nucleolar stress and ferroptosis. Redox Biol. 2023;62: Article 102705. 37087976 10.1016/j.redox.2023.102705 PMC10149416

[44] Hao Q, Wang J, Chen Y, Wang S, Cao M, Lu H, Zhou X. Dual regulation of p53 by the ribosome maturation factor SBDS. Cell Death Dis. 2020;11: Article 197. 32198344 10.1038/s41419-020-2393-4 PMC7083877

[45] Zhou X, Hao Q, Liao J, Zhang Q, Lu H. Ribosomal protein S14 unties the MDM2-p53 loop upon ribosomal stress. Oncogene. 2013;32(3):388–396. 22391559 10.1038/onc.2012.63 PMC3736832

[46] Shen WX, Li GH, Li YJ, Zhang PF, Yu JX, Shang D, Wang QS. Prognostic significance of tumor mutation burden among patients with non-small cell lung cancer who received platinum-based adjuvant chemotherapy: An exploratory study. J Cancer Prev. 2023;28(4):175–184. 38205359 10.15430/JCP.2023.28.4.175 PMC10774481

[47] Su X, Jin K, Guo Q, Xu Z, Liu Z, Zeng H, Wang Y, Zhu Y, Xu L, Wang Z, et al. Integrative score based on CDK6, PD-L1 and TMB predicts response to platinum-based chemotherapy and PD-1/PD-L1 blockade in muscle-invasive bladder cancer. Br J Cancer. 2024;130(5):852–560. 38212482 10.1038/s41416-023-02572-9 PMC10912081

[48] Tanwar NA, Malhotra R, Satheesh AP, Khuntia SP, Sreekanthreddy P, Varghese L, Kolla S, Chandrani P, Choughule A, Pange P, et al. Understanding the impact of population and cancer type on tumor mutation burden scores: A comprehensive whole-exome study in cancer patients from India. JCO Glob Oncol. 2023;9: Article e2300047. 38085046 10.1200/GO.23.00047 PMC10846780

[49] Niu X, Martinez L. Harnessing p53 to improve immunotherapy for lung cancer treatment. Cancer Res. 2023;84(2):179–180. 10.1158/0008-5472.CAN-23-3929 38095514

[50] Rizvi NA, Hellmann MD, Snyder A, Kvistborg P, Makarov V, Havel JJ, Lee W, Yuan J, Wong P, Ho TS, et al. Cancer immunology. Mutational landscape determines sensitivity to PD-1 blockade in non-small cell lung cancer. Science. 2015;348(6230):124–128. 25765070 10.1126/science.aaa1348 PMC4993154

[51] Carbone DP, Reck M, Paz-Ares L, Creelan B, Horn L, Steins M, Felip E, van den Heuvel M, Ciuleanu TE, Badin F, et al. First-line nivolumab in stage IV or recurrent non-small-cell lung cancer. N Engl J Med. 2017;376(25):2415–2426. 28636851 10.1056/NEJMoa1613493 PMC6487310

[52] Yarchoan M, Hopkins A, Jaffee EM. Tumor mutational burden and response rate to PD-1 inhibition. N Engl J Med. 2017;377(25):2500–2501. 29262275 10.1056/NEJMc1713444 PMC6549688

[53] Alexandrov LB, Stratton MR. Mutational signatures: The patterns of somatic mutations hidden in cancer genomes. Curr Opin Genet Dev. 2014;24:52–60. 24657537 10.1016/j.gde.2013.11.014 PMC3990474

[54] Stelloo E, Jansen AML, Osse EM, Nout RA, Creutzberg CL, Ruano D, Church DN, Morreau H, Smit VTHBM, van Wezel T, et al. Practical guidance for mismatch repair-deficiency testing in endometrial cancer. Ann Oncol. 2017;28(1):96–102. 27742654 10.1093/annonc/mdw542

[55] Cancer Genome Atlas Research. Comprehensive molecular characterization of clear cell renal cell carcinoma. Nature. 2013;499(7456):43–49. 23792563 10.1038/nature12222 PMC3771322

[56] Murugan P, Jia L, Dinatale RG, Assel M, Benfante N, al-Ahmadie HA, Fine SW, Gopalan A, Sarungbam J, Sirintrapun SJ, et al. Papillary renal cell carcinoma: A single institutional study of 199 cases addressing classification, clinicopathologic and molecular features, and treatment outcome. Mod Pathol. 2022;35(6):825–835. 34949764 10.1038/s41379-021-00990-9 PMC9177523

[57] Marcon J, DiNatale R, Sanchez A, Kotecha RR, Gupta S, Kuo F, Makarov V, Sandhu A, Mano R, Silagy AW, et al. Comprehensive genomic analysis of translocation renal cell carcinoma reveals copy-number variations as drivers of disease progression. Clin Cancer Res. 2020;26(14):3629–3640. 32220885 10.1158/1078-0432.CCR-19-3283 PMC7367714

[58] Polascik TJ, Cairns P, Epstein JI, Fuzesi L, Ro JY, Marshall FF, Sidransky D, Schoenberg M. Distal nephron renal tumors: Microsatellite allelotype. Cancer Res. 1996;56(8):1892–1895. 8620510

[59] Elfakharany HK, Ghoraba HM, Gaweesh KA, Eldeen AAS, Eid AM. Immunohistochemical expression of cytochrome P4A11 (CYP4A11), carbonic anhydrase 9 (CAIX) and Ki67 in renal cell carcinoma; diagnostic relevance and relations to clinicopathological parameters. Pathol Res Pract. 2024;253: Article 155070. 38183818 10.1016/j.prp.2023.155070

[60] Xu N, Yao Z, Shang G, Ye D, Wang H, Zhang H, Qu Y, Xu F, Wang Y, Qin Z, et al. Integrated proteogenomic characterization of urothelial carcinoma of the bladder. J Hematol Oncol. 2022;15(1): Article 76. 35659036 10.1186/s13045-022-01291-7 PMC9164575

[61] Deng M, Ran P, Chen L, Wang Y, Yu Z, Cai K, Feng J, Qin Z, Yin Y, Tan S, et al. Proteogenomic characterization of cholangiocarcinoma. Hepatology. 2022;77(2):411–429. 35716043 10.1002/hep.32624 PMC9869950

[62] Shi Y, Wu Y, Li F, Jiang K, Fang X, Wang Y, Song X, Wang R, Chen L, Zheng Z, et al. Investigating the immunogenic cell death-dependent subtypes and prognostic signature of triplenegative breast cancer. Phenomics. 2024;4(1):34–35. 38605910 10.1007/s43657-023-00133-x PMC11003942

[63] Wu J, Liu W, Qiu X, Li J, Yu Z, Song K, Shen S, Huo L, Chen L, Xu M, Wang H, et al. A noninvasive approach to evaluate tumor immune microenvironment and predict outcomes in hepatocellular carcinoma. Phenomics. 2023;3(6):549–564. 38223688 10.1007/s43657-023-00136-8 PMC10781918

[64] Hu HH, et al. *Holist Integr Oncol*. 2025.

[65] Morana O, Wood W, Gregory CD. The apoptosis paradox in cancer. Int J Mol Sci. 2022;23(3): Article 1328. 35163253 10.3390/ijms23031328 PMC8836235

[66] An Y, Xia Y, Wang Z, Jin GZ, Shang M. Clinical significance of ribosome production factor 2 homolog in hepatocellular carcinoma. Clin Res Hepatol Gastroenterol. 2024;48(3): Article 102289. 38307254 10.1016/j.clinre.2024.102289

[67] Lu M, Hu X, Cheng C, Zhang Y, Huang L, Kong X, Li Z, Zhang Q, Zhang Y. RPF2 mediates the CARM1-MYCN axis to promote chemotherapy resistance in colorectal cancer cells. Oncol Rep. 2024;51(1):1–10. 37997821 10.3892/or.2023.8670 PMC10696550

[68] Li H, Hu X, Cheng C, Lu M, Huang L, Dou H, Zhang Y, Wang T. Ribosome production factor 2 homolog promotes migration and invasion of colorectal cancer cells by inducing epithelial-mesenchymal transition via AKT/Gsk-3β signaling pathway. Biochem Biophys Res Commun. 2022;597:52–57. 35123266 10.1016/j.bbrc.2022.01.090

[69] Li H, Durbin R. Fast and accurate short read alignment with Burrows-Wheeler transform. Bioinformatics. 2009;25(14):1754–1760. 19451168 10.1093/bioinformatics/btp324 PMC2705234

[70] Etherington GJ, Ramirez-Gonzalez RH, MacLean D. Bio-samtools 2: A package for analysis and visualization of sequence and alignment data with SAMtools in ruby. Bioinformatics. 2015;31(15):2565–2567. 25819670 10.1093/bioinformatics/btv178

[71] Wang K, Li M, Hakonarson H. ANNOVAR: Functional annotation of genetic variants from high-throughput sequencing data. Nucleic Acids Res. 2010;38(16): Article e164. 20601685 10.1093/nar/gkq603 PMC2938201

[72] Mayakonda A, Lin DC, Assenov Y, Plass C, Koeffler HP. Maftools: Efficient and comprehensive analysis of somatic variants in cancer. Genome Res. 2018;28(11):1747–1756. 30341162 10.1101/gr.239244.118 PMC6211645

[73] Alexandrov LB, Nik-Zainal S, Wedge DC, Campbell PJ, Stratton MR. Deciphering signatures of mutational processes operative in human cancer. Cell Rep. 2013;3(1):246–259. 23318258 10.1016/j.celrep.2012.12.008 PMC3588146

[74] Tate JG, Bamford S, Jubb HC, Sondka Z, Beare DM, Bindal N, Boutselakis H, Cole CG, Creatore C, Dawson E, et al. COSMIC: The catalogue of somatic mutations in cancer. Nucleic Acids Res. 2019;47(D1):D941–D947. 30371878 10.1093/nar/gky1015 PMC6323903

[75] Chalmers ZR, Connelly CF, Fabrizio D, Gay L, Ali SM, Ennis R, Schrock A, Campbell B, Shlien A, Chmielecki J, et al. Analysis of 100,000 human cancer genomes reveals the landscape of tumor mutational burden. Genome Med. 2017;9(1): Article 34. 10.1186/s13073-017-0424-2 PMC5395719 28420421

[76] Pertea M, Pertea GM, Antonescu CM, Chang TC, Mendell JT, Salzberg SL. StringTie enables improved reconstruction of a transcriptome from RNA-seq reads. Nat Biotechnol. 2015;33(3):290–295. 25690850 10.1038/nbt.3122 PMC4643835

[77] Wisniewski JR, Zougman A, Nagaraj N, Mann M. Universal sample preparation method for proteome analysis. Nat Methods. 2009;6(5):359–362. 19377485 10.1038/nmeth.1322

[78] Zhang W, Zhang J, Xu C, Li N, Liu H, Ma J, Zhu Y, Xie H. LFQuant: A label-free fast quantitative analysis tool for high-resolution LC-MS/MS proteomics data. Proteomics. 2012;12(23–24):3475–3484. 23081734 10.1002/pmic.201200017

[79] Schwanhäusser B, Busse D, Li N, Dittmar G, Schuchhardt J, Wolf J, Chen W, Selbach M. Global quantification of mammalian gene expression control. Nature. 2011;473(7347):337–342. 21593866 10.1038/nature10098

[80] Krug K, Jaehnig EJ, Satpathy S, Blumenberg L, Karpova A, Anurag M, Miles G, Mertins P, Geffen Y, Tang LC, et al. Proteogenomic landscape of breast cancer tumorigenesis and target ed therapy. Cell. 2020;183(5):1436–1456.e31. 33212010 10.1016/j.cell.2020.10.036 PMC8077737

[81] Satpathy S, Krug K, Beltran PM, Savage SR, Petralia F, Kumar-Sinha C, Dou Y, Reva B, Kane MH, Avanessian SC, et al. A proteogenomic portrait of lung squamous cell carcinoma. Cell. 2021;184(16):4348–4371.e40. 34358469 10.1016/j.cell.2021.07.016 PMC8475722

[82] Ma W, Kim S, Chowdhury S, Li Z, Yang M, Yoo S, Petralia F, Jacobsen J, Li JJ, Ge X, et al. DreamAI: Algorithm for the imputation of proteomics data. bioRxiv. 2021. 10.1101/2020.07.21.214205

[83] Leek JT, Johnson WE, Parker HS, Jaffe AE, Storey JD. The sva package for removing batch effects and other unwanted variation in high-throughput experiments. Bioinformatics. 2012;28(6):882–883. 22257669 10.1093/bioinformatics/bts034 PMC3307112

[84] Subramanian A, Tamayo P, Mootha VK, Mukherjee S, Ebert BL, Gillette MA, Paulovich A, Pomeroy SL, Golub TR, Lander ES, et al. Gene set enrichment analysis: A knowledge-based approach for interpreting genome-wide expression profiles. Proc Natl Acad Sci USA. 2005;102(43):15545–15550. 16199517 10.1073/pnas.0506580102 PMC1239896

[85] Buccitelli C, Selbach M. mRNAs, proteins and the emerging principles of gene expression control. Nat Rev Genet. 2020;21(10):630–644. 32709985 10.1038/s41576-020-0258-4

[86] Kamburov A, Pentchev K, Galicka H, Wierling C, Lehrach H, Herwig R. ConsensusPathDB: Toward a more complete picture of cell biology. Nucleic Acids Res. 2011;39(Suppl 1):D712–D717. 21071422 10.1093/nar/gkq1156 PMC3013724

[87] Huang da W, Sherman BT, Lempicki RA. Systematic and integrative analysis of large gene lists using DAVID bioinformatics resources. Nat Protoc. 2009;4(1):44–57. 19131956 10.1038/nprot.2008.211

[88] Ogata H, Goto S, Sato K, Fujibuchi W, Bono H, Kanehisa M. KEGG: Kyoto Encyclopedia of Genes and Genomes. Nucleic Acids Res. 1999;27(1):29–34. 9847135 10.1093/nar/27.1.29 PMC148090

[89] Liberzon A, Birger C, Thorvaldsdóttir H, Ghandi M, Mesirov JP, Tamayo P. The molecular signatures database (MSigDB) hallmark gene set collection. Cell Syst. 2015;1(6):417–425. 26771021 10.1016/j.cels.2015.12.004 PMC4707969

[90] Milacic M, Beavers D, Conley P, Gong C, Gillespie M, Griss J, Haw R, Jassal B, Matthews L, May B, et al. The Reactome pathway knowledgebase 2024. Nucleic Acids Res. 2024;52(D1):D672–d678. 37941124 10.1093/nar/gkad1025 PMC10767911

[91] Ashburner M, Ball CA, Blake JA, Botstein D, Butler H, Cherry JM, Davis AP, Dolinski K, Dwight SS, Eppig JT, et al. Gene ontology: Tool for the unification of biology. Nat Genet. 2000;25(1):25–29. 10802651 10.1038/75556 PMC3037419

[92] Hanzelmann S, Castelo R, Guinney J. GSVA: Gene set variation analysis for microarray and RNA-Seq data. BMC Bioinform. 2013;14(1): Article 7. 10.1186/1471-2105-14-7 PMC3618321 23323831

[93] Linding R, Jensen LJ, Ostheimer GJ, van Vugt M, Jørgensen C, Miron IM, Diella F, Colwill K, Taylor L, Elder K, et al. Systematic discovery of in vivo phosphorylation networks. Cell. 2007;129(7):1415–1426. 17570479 10.1016/j.cell.2007.05.052 PMC2692296

[94] Hornbeck PV, Zhang B, Murray B, Kornhauser JM, Latham V, Skrzypek E. PhosphoSitePlus, 2014: Mutations, PTMs and recalibrations. Nucleic Acids Res. 2015;43(D1):D512–D520. 25514926 10.1093/nar/gku1267 PMC4383998

[95] Wilkerson MD, Hayes DN. ConsensusClusterPlus: A class discovery tool with confidence assessments and item tracking. Bioinformatics. 2010;26(12):1572–1573. 20427518 10.1093/bioinformatics/btq170 PMC2881355

[96] Ma J, Chen T, Wu S, Yang C, Bai M, Shu K, Li K, Zhang G, Jin Z, He F, et al. iProX: An integrated proteome resource. Nucleic Acids Res. 2019;47(D1):D1211–d1217. 30252093 10.1093/nar/gky869 PMC6323926

