# Supplementary material for: Proteogenomic Analysis Identifies Clinically Relevant Subgroups of Collecting Duct Carcinoma
Source: Research (Wash D C). 2025 Sep 3;8:0859. doi: 10.34133/research.0859 (PMC12408255; doi:10.34133/research.0859)
Supplement: Supplementary 1 — Figs. S1 to S8 Data S1 to S6 [file research.0859.f1.zip › Supplementary Instruction-0721-accepted-clean.docx]

**SUPPLEMENTARY INSTRUCTION**

**Section I: Comparison of genomic alterations**

**About alterations in *CDKN2A/B***

The alterations in *CDKN2A/B*, especially in *CDKN2A*, have been reported in previous studies in CDC. Specifically, Pal et al.’s study (*European Urology*, 2016, PMID: 26149668) showed that 12% (2 out of 17) of CDC cases had genomic alterations in *CDKN2A*, including one homozygous deletion and one truncation. Wang et al.’s study (*Oncotarget*, 2016, PMID: 27144525) identified 8 homozygous losses (50%) and 2 heterozygous losses (12.5%) of *CDKN2A* in 16 patients with CDC. In unclassified RCC from AACR Project GENIE database (https://www.cbioportal.org/), no somatic mutations in *CDKN2A/B* were detected, while 4.8% of cases had homozygous losses of *CDKN2A/B*. Furthermore, Zhang et al.’s study (*BMC Medical Genomics*, 2022, PMID: 34980126) detected only one frameshift-deleted *CDKN2A* mutation in 10 Chinese patients with CDC, while the frequently deleted focal region was found in 9p21.3, containing *CDKN2A* and *CDKN2B*. These results indicated that there was a higher percentage of CNV alterations than SNV alterations of *CDKN2A/B* in CDC cohorts. Additionally, due to the small sample size of the previous studies, ranging from 10 to 17, the alteration frequency of *CDKN2A/B* varies. Notably, our study provided the largest cohort of CDC to date. In our cohort, although no somatic mutations in *CDKN2A* or *CDKN2B* were detected, losses of *CDKN2A* and *CDKN2B* were detected in 31.25% (15 out of 48) of CDC patients at the CNV-based gene level (**Figure S1A**). Taken together, both previous studies and our study revealed that losses of *CDKN2A*, rather than somatic mutations in *CDKN2A*, were more often identified in CDC, suggesting that losses of *CDKN2A* may play a more important role in CDC than its somatic mutations.

**About alterations in *SMARCB1***

*SMARCB1* mutation exhibits discrepancy in different CDC cohorts. Pal et al.’s study (*European Urology*, 2016, PMID: 26149668) showed that 18% (3 out of 17) of CDC cases had *SMARCB1* truncation, and 4.8% of cases in unclassified renal cell carcinoma subtype from AACR Project GENIE database (https://www.cbioportal.org/) developed *SMARCB1* mutation. However, *SMARCB1* mutations were not reported in either Wang et al.’s study (Western cohort) (*Oncotarget*, 2016, PMID: 27144525) or Zhang et al.’s study (Chinese cohort) (*BMC Medical Genomics*, 2022, PMID: 34980126). These results suggested that this discrepancy could be due to either small sample sizes or racial differences. Similar to the results from the Chinese cohort (*BMC Medical Genomics*, 2022, PMID: 34980126), somatic mutations of *SMARCB1* were not detected in our cohort, illustrating the consistency of *SMARCB1* mutations in the same racial context.

**About alterations of *KMT2C/D***

Despite mutations in *KMT2C/D* were detected in our cohort, which has not been reported in previous studies of CDC, we observed that more than half (57.14%) of *KMT2C/D* mutations occurred in a co-occurrence manner to alterations in *NF2* and *SETD2* (**Figure S1B**). Notably, alterations in *NF2* and *SETD2* are rarely reported in UC (*Cancer Cell*, 2021, PMID: 34129823; *Journal of Hematology & Oncology*, 2022, PMID: 35659036), but they have been identified as characteristic frequent mutations of CDC in several CDC cohorts (*European Urology*, 2016, PMID: 26149668; *BMC Medical Genomics*, 2022, PMID: 34980126), including our cohort. This suggested the existence of *KMT2C/D* mutations in CDC, which were not caused by misdiagnosis, and further illustrated a complex and possibly heterogeneous genetic landscape for CDC.

**About alterations in *VHL***

Although *VHL* mutations typically occur in ccRCC, previous studies have also reported *VHL* mutations in CDC. Specifically, in Pal et al.’s study (*European Urology*, 2016, PMID: 26149668), *VHL* was identified at a frequency of 6% (1/17 patients carried mutations in *VHL*). Moreover, in unclassified RCC cases from AACR Project GENIE database (https://www.cbioportal.org/), 2.5% of cases developed *VHL* mutations. Similar to these reports, *VHL* mutations were detected in 2 (CDC_#49 and CDC_#51) out of 48 patients in our cohort, with a frequency of 4%. These results suggested that despite *VHL* mutations were thought to be related to ccRCC, they may also occasionally manifest in CDC, albeit infrequently. The H&E and IHC staining images of these two cases were shown in the **Supplementary Data 1**.

Through comparison of the main gene mutations mentioned above, we believe that there are high similarities between the genomic alteration profiles of our cohort and those of previous studies, including frequent losses of *CDKN2A/B*, as well as frequent mutations in *NF2* and *SETD2*. As for the *AHNAK2* mutation specifically identified in our cohort, it co-occurs with the majority of frequent gene mutations and is mutually exclusive with *NF2* mutations. This may suggest a novel subgroup in CDC besides those with *NF2* mutations, which needs to be further demonstrated in larger, diverse ethnic cohorts in the future.

Additionally, as for the discrepancy of genomic alteration profiles in CDC, it is prevalently present in published studies. For example, in the three CDC cohorts mentioned above, a total of 67 mutated genes were identified, of which only 6 genes (9%, including *NF2*, *ATM*, *FBXW7*, *TP53*, *CDKN2A* and *SETD2*) were detected recurrently in different cohorts, and 2 genes (3%, including *FBXW7* and *TP53*) were detected in all three cohorts (**Figure S1C**). This discrepancy may be due to the small sample sizes, with most gene mutations identified only in a single patient. Notably, among these 6 mutated genes, 4 (*NF2*, *FBXW7*, *TP53*, *SETD2*) were recurrently identified in our cohort, including 2 genes (*FBXW7* and *TP53*) that were identified in all three cohorts. Additionally, despite no somatic mutation in *CDKN2A/B* was identified in our cohort, frequent losses of *CDKN2A/B* were detected, which aligned with previous studies by Wang et al. and Zhang et al. Taken together, these results further illustrate the accuracy of the CDC diagnosis in this cohort.

**
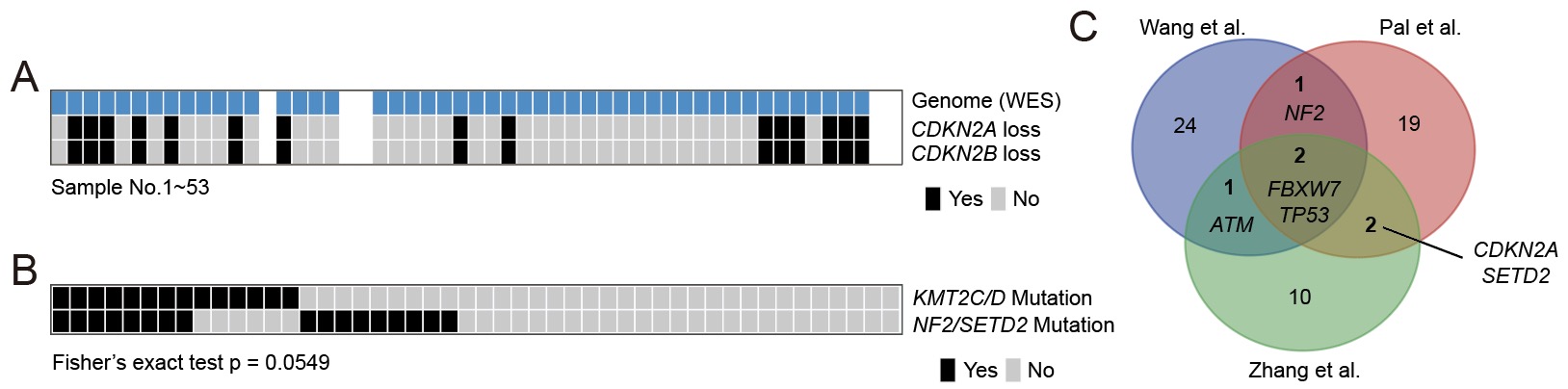
**

**Figure S1**

A. Patients with *CDKN2A/B* loss among those undergoing WES analysis.

B. Comparison of *KMT2C/D* mutations and alterations in *NF2* and *SETD2* (Fisher’s exact test).

C. Venn diagram showing the mutated genes identified in three CDC cohorts.

**Section II: About the potential nephron sites of origin of RCCs**

In the study of Lack *et al.*, droplet-based transcriptome assay (Chromium v3) for single nuclei (snCV3) was used, permitting the discovery of 100 distinct cell populations. These cell types were distributed along the depth of the kidney from the cortex to the papillary tip, in each nephron segment and the interstitium. The minimal combinatorial marker genes were identified using NSForestv2 software for all cell types. As a result, one hundred and eighty-five marker genes were filtered to distinguish the 99 distinct cell populations (except mast cell), which were annotated to 7 substructures (including proximal tubules, intermediate tubules, distal tubules, collecting tubules, interstitium, renal corpuscle, and vessels) and 5 putative cell states, namely cycling, transitioning, adaptive (successful or maladaptive repair), degenerative (damage or stressed), and reference (**Figure S2A**).

Based on these marker genes, we investigated the cell type composition of CDC tissues and common kidney cancers (including KIRC, KIRP, and KICH) to infer the potential nephron site of origin of these malignancies. By analyzing the positive expression of these marker genes in different RCC tissues, we matched twenty-five cell populations in CDC, thirty-four in KIRC, twenty-two in KIRP, and eighteen in KICH, respectively (**Figure S2B**). Further comparative analysis showed that two cell populations were specifically matched in KICH, namely Outer Medullary Collecting Duct Intercalated Cell Type A (OMCD-IC-A) and Degenerative Cortical Intercalated Cell Type A (dC-IC-A), which was consistent with the known origin of KICH from intercalated cells. Degenerative Inner Medullary Collecting Duct Cell (dIMCD) was identified in both CDC and KICH, but not in KIRC and KIRP, indicating medullary collecting duct was the putative common site of origin for CDC and KICH. Moreover, we observed Adaptive / Maladaptive / Repairing Thick Ascending Limb Cell (aTAL1) and Cycling Distal Convoluted Tubule Cell (cycDCT) were specifically matched in CDC, which suggested that in addition to medullary collecting duct, distal tubules were also potential sites of origin for CDC. Furthermore, we observed specific aggregation of Proximal Tubule Epithelial Cell (PT) in KIRC, which was consistent with the previous reports that KIRC originated in the more proximal (cortical) regions of the nephron (*Science*, 2018, PMID: 30093597; *Cancer Cell*, 2020, 32359397). In addition, we found that individual tumor types contained cells in multiple states, further suggesting the presence of intra-tumoral heterogeneity (**Figure S2C**).

Overall, the analysis using marker genes from Lake et al. indicated that CDC may not only originate from medullary collecting duct epithelial cell (such as inner medullary collecting duct cell), which has been well-documented, but also be closely related to distal tubular epithelial cells (including thick ascending limb cell and distal convoluted tubule cell).

**
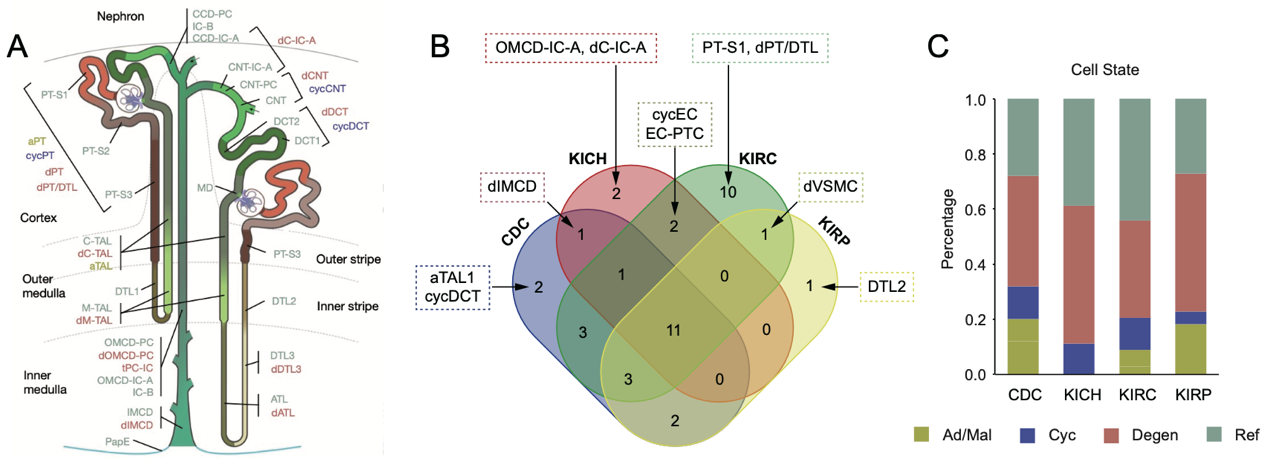
**

**Figure S2**

A. Schematic of the human nephron showing cell types.

B. Venn diagram showing cell populations identified in distinct kidney malignancies. KIRC: clear cell renal cell carcinoma; KIRP: papillary renal cell carcinoma; KICH: chromophobe renal cell carcinoma.

C. Comparison of cell states in different RCC types.

**Section III: About the TMB cutoff**

The rationale for this selection can be divided into three parts: (1) CDC showed a lower median TMB (0.85 mut/Mb) compared to the majority of tumors in TCGA; (2) the cutoff of 0.6 mut/Mb could associate TMB with clinical outcome in CDC; (3) comparison of proteomics using 0.6 mut/Mb as TMB cutoff had similar molecular characters with that of using 10 mut/Mb as TMB cutoff.

Firstly, in this CDC cohort, TMB showed a high heterogeneity, ranging from 0.03 to 76.4, with a median value of 0.85 (**Figure 1c**). More than half of the patients (26/48) had TMB values less than 1. To further clarify the TMB level of CDC, we compared the distribution of TMB between CDC and other tumors in TCGA, and found that CDC had a lower median TMB value compared to most tumors in TCGA, including KIRC and KIRP (**Supplementary Figure 1b**). These results indicated that CDC was a cancer type with low TMB and values closed to the median (0.85 mut/Mb) could be considered as the TMB cutoff.

Secondly, we further associated TMB cutoff with progression free survival (PFS), and found that using 0.6 mut/Mb, closed to the median, as the TMB cutoff could effectively distinguish PFS of CDC patients (**Figure 2a**). Twenty-nine out of 48 patients had TMB values greater than 0.6 and had significantly shorter PFS (**Figure 2a**, log-rank test p value = 0.0366). Overall, based on (1) the low median value of TMB and (2) the ability to distinguish PFS of CDC patients, 0.6 mut/Mb was considered as the TMB cutoff for downstream analysis.

Thirdly, we verified the robustness of 0.6 mut/Mb as TMB cutoff. As previous reported, although the optimal cutoff value varies depending on the TMB distribution of the different tumor types, TMB ≥ 10 mut/Mb has been approved as diagnostic biomarker for pembrolizumab in the treatment of advanced cancer, including RCC (*Nature Cancer*, 2024, PMID: 38528112). Therefore, we used 10 mut/Mb as TMB cutoff to analyze whether it had similar impacts on the clinical outcome and molecular expression profile in this cohort, compared to the results using 0.6 mut/Mb as TMB cutoff. Over-representation analysis (ORA) revealed that ribosome biogenesis was the top pathway enriched in patients with TMB ≥ 10 mut/Mb (**Figure S3A**), which was consistent with that using 0.6 mut/Mb as TMB cutoff (**Figure 2c**). Moreover, RPF2 (Wilcoxon test p = 0.008) and OTUB1 (Wilcoxon test p = 0.027), overexpressed in patients with TMB ≥ 0.6 mut/Mb, were also significantly upregulated in patients with TMB ≥ 10 mut/Mb (**Figure S3B, Figure 2c**). However, we found that the TMB cutoff of 10 mut/Mb couldn’t distinguish OS and PFS (log-rank test p > 0.05) of CDC patients (**Figure S3C**), while using 0.6 mut/Mb as TMB cutoff could distinguish PFS (log-rank test p value = 0.0366). These results indicated that compared to using 0.6 mut/Mb as TMB cutoff, using 10 mut/Mb as TMB cutoff had similar impacts on molecular expression profile, however, the latter couldn’t distinguish clinical outcome of patients. Furthermore, compared to 29 patients with TMB greater than 0.6, only 13 patients with TMB greater than 10, which resulted in the majority of patients being unable to be explained under this cutoff (10 mut/Mb). These results suggested the need to dynamically define the optimal cutoff value, especially for rare cancers.


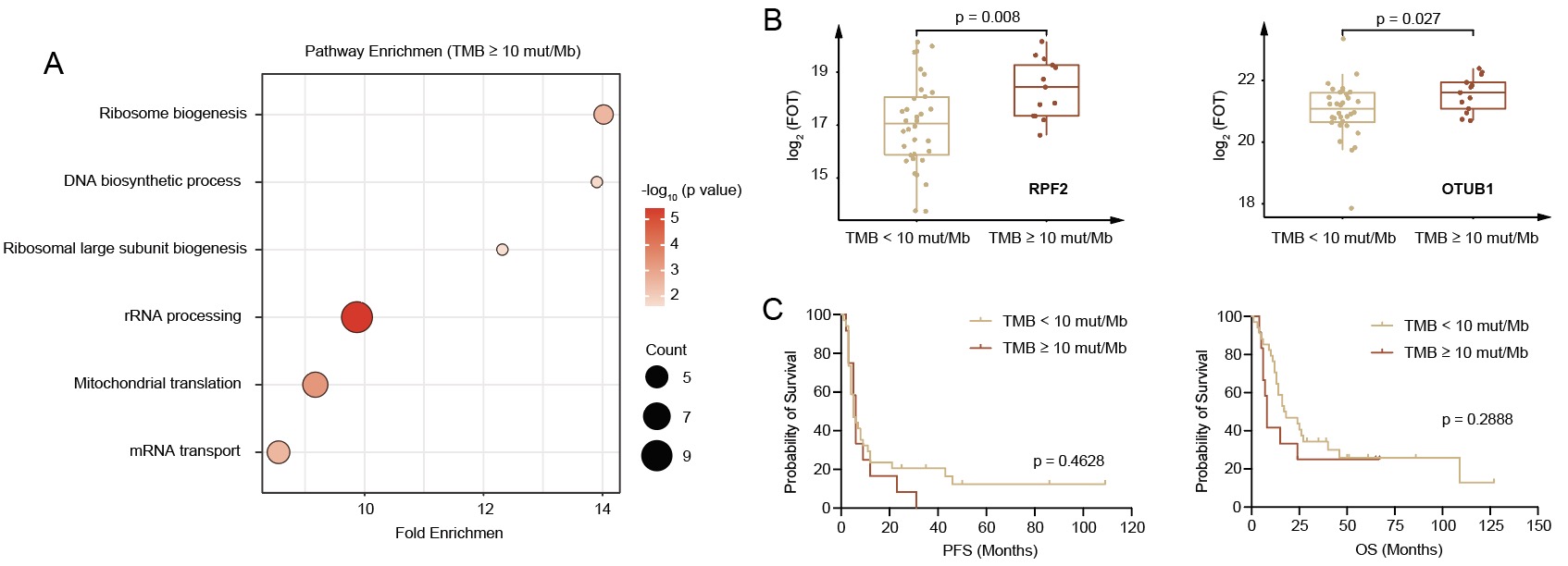


**Figure S3**

A. Bubble chart showing the enriched biological pathways in tumors with TMB ≥ 10 mut/Mb.

B. Comparison of RPF2 and OTUB1 protein abundances in TMB < 10 mut/Mb (n = 35) and TMB ≥ 10 mut/Mb (n = 13) tumors (Wilcoxon test).

C. Kaplan-Meier curves of PFS and OS for patients with distinct TMB scores (two-sided log-rank test).

**Section IV: About the differential protein expression profiles between TNM Stage I/II and III/IV tumors across ccRCC, tRCC, and CDC**

In **Figure 4i**, tumor samples from three RCC types (ccRCC, tRCC, and CDC) were stratified into two groups according to TNM staging (I/II vs. III/IV). Within each group, proteins exhibiting differentially expressed across the types were identified, retaining only those demonstrating markedly elevated expression in one type compared to both others (FC > 2, Wilcoxon rank-sum test, FDR < 0.05). The results demonstrated that 236, 62, and 167 upregulated proteins were identified in ccRCC, tRCC, and CDC, respectively, within stage I/II tumors (**Supplementary Data 4**). Corresponding analysis of stage III/IV tumors revealed 339, 81, and 303 upregulated proteins in these RCC types (**Supplementary Data 4**). Comparative analysis of upregulated proteins identified in stage I/II versus stage III/IV tumors revealed inter-stage overlap rates of 36.3% in ccRCC, 34.9% in tRCC, and 31.3% in CDC (**Figure S4A**). Moreover, we found that in stage I/II tumors, 83, 25, and 55 proteins were specifically upregulated in ccRCC, tRCC, and CDC, respectively, and their expression was also relatively higher in corresponding types of stage III/IV tumors, with 82, 25, and 51 proteins having fold-changes (FCs, one type vs. the remaining types) greater than 1, respectively. Similarly, in stage III/IV tumors, 186, 44, and 191 proteins were specifically elevated in ccRCC, tRCC, and CDC, respectively, and their expression was also relatively higher in corresponding types of stage III/IV tumors, with 182, 40, and 165 proteins having FCs greater than 1, respectively. Collectively, these findings indicated high similarity in differential protein expression profiles between TNM Stage I/II and III/IV tumors across all three RCC types.

Furthermore, pathway enrichment analysis of differentially expressed proteins across the three RCC types revealed substantially similar biological pathways enriched among upregulated proteins in both stage I/II and stage III/IV tumors (**Figure S4B**). Across both tumor stages (I/II and III/IV), upregulated proteins in ccRCC consistently demonstrated enrichment in metabolic and angiogenesis-related pathways, tRCC exhibited significant enrichment of upregulated proteins in oxidative phosphorylation (OXPHOS), and CDC showed predominant enrichment of upregulated proteins in pro-proliferative and immune-related pathways, including DNA replication, ribosome biogenesis, extracellular matrix (ECM) organization, cell adhesion, and innate immune response.

Collectively, the differential protein expression profiles and enriched biological pathways exhibited striking similarities between stage I/II and stage III/IV tumors across all three RCC types. This stage-independent conservation suggested limited influence of tumor staging on the observed proteomic features. Consequently, the differential protein expression profiles appeared to represent RCC type-specific differences rather than results of tumor progression stages.


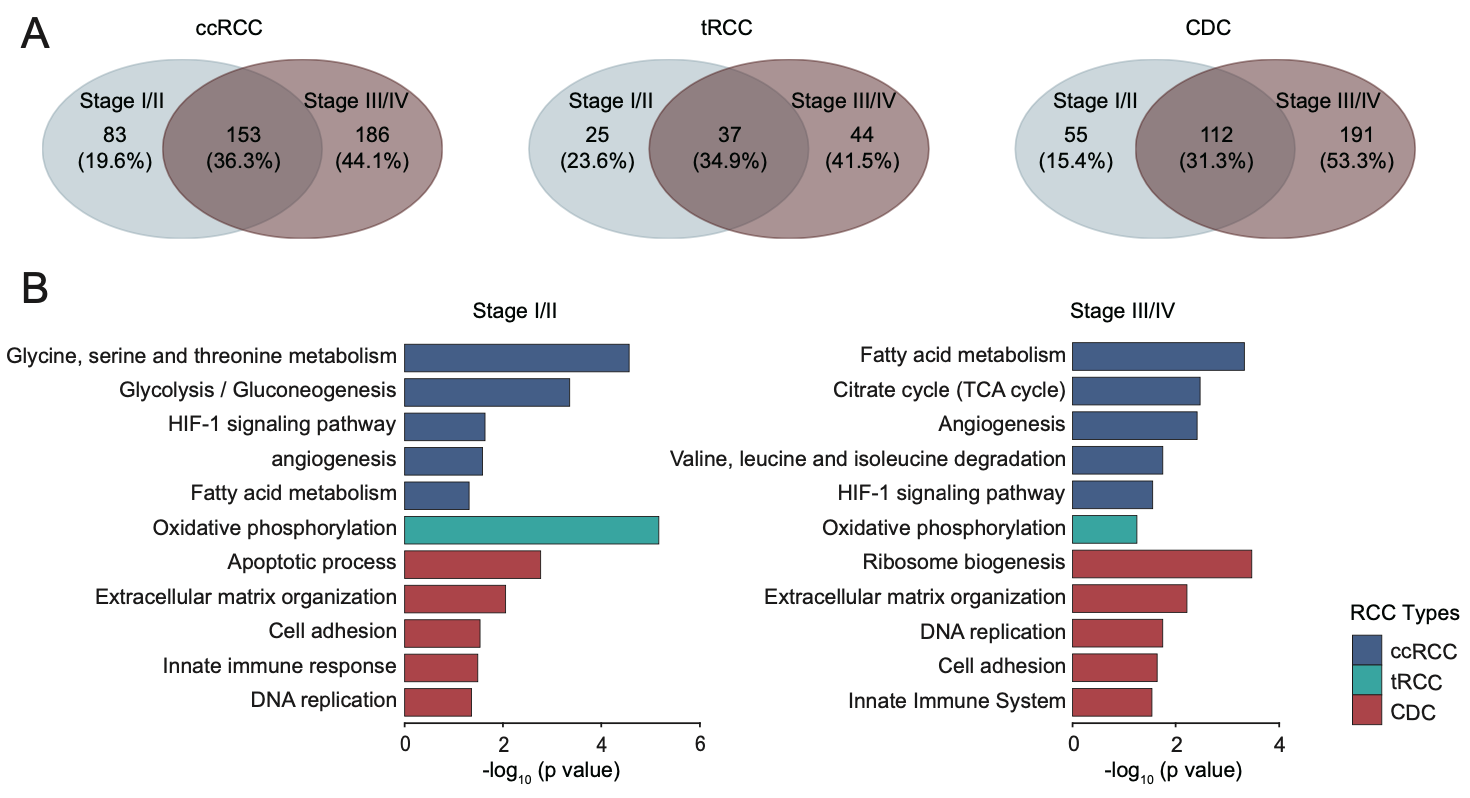


**Figure S4**

A. Venn diagram showing the overlap of upregulated proteins in the three RCC types between stage I/II and stage III/IV tumors. ccRCC, clear cell renal cell carcinoma; tRCC, translocation renal cell carcinoma; CDC, collecting duct carcinoma.

B. Biological pathways enriched among upregulated proteins demonstrated conservation across stage I/II and stage III/IV tumors in all three RCC types.

**Section V: About the biological impact of *AHNAK2* mutations in CDC**

*AHNAK2*, also known as *C14orf78*, encodes a large scaffold protein that participates in various cellular processes, including muscle membrane repair and calcium signal transduction (*Journal Of Gene Medicine*, 2022, PMID: 35882062; *Faseb Journal*, 2005, PMID: 16319140). Recent studies have shown that AHNAK2 protein may play the role of a proto-oncogene in various tumor types, including ccRCC (*Theranostics*, 2017, PMID: 28435451), thyroid cancer (*Life Sciences*, 2021, PMID: 34627772), and melanoma (*Cancer Biotherapy and Radiopharmaceuticals*, 2019, PMID: 31621397). Moreover, an analysis of lung adenocarcinoma (LUAD) showed that *AHNAK2* expression demonstrated significant positive correlation with cell adhesion-related pathways, while showed significant negative correlations with oxidative phosphorylation, amino acid metabolism, and infiltration of multiple immune cell types, including activated B cell, activated CD8 + T cell, and immature B cell (*International Immunopharmacology*, 2021, PMID: 33168407). However, the role of *AHNAK2* mutations in CDC remains unclear.

To investigate the potential functional significance of *AHNAK2* mutations in CDC, we performed multi-omics comparative analysis of patients with *AHNAK2*-mutant versus wild-type profiles in this cohort. Firstly, we found that in our cohort, AHNAK2 mutation status demonstrated no significant association with OS or PFS (**Figure S5A**). Proteomic analysis revealed significant upregulation of metabolic and HIF-1 signaling pathways in patients with *AHNAK2*-mutant patients, whereas wild-type cases exhibited predominant upregulation of biological oxidation, ECM-receptor interaction, and focal adhesion. Phosphoproteomic profiling identified significant enrichment of immune-related signaling in *AHNAK2*-mutant patients, with concurrent upregulation of VEGFA-VEGFR2 pathway and signaling by receptor tyrosine kinase. By contrast, wild-type patients demonstrated enhanced phosphorylation in focal adhesion, membrane trafficking, tight junction, and ErbB signaling pathway. Transcriptomic data corroborated immune signaling activation in mutant cases, while wild-type patients showed elevated MAPK family signaling cascades, ErbB signaling pathway, and focal adhesion (**Figure S5B**). Collectively, these multi-omics analyses indicated that *AHNAK2* mutations associated with activation of immune signaling and enhanced metabolic pathways, while preservation of *AHNAK2* structural integrity correlated with strengthened intercellular adhesion signaling.


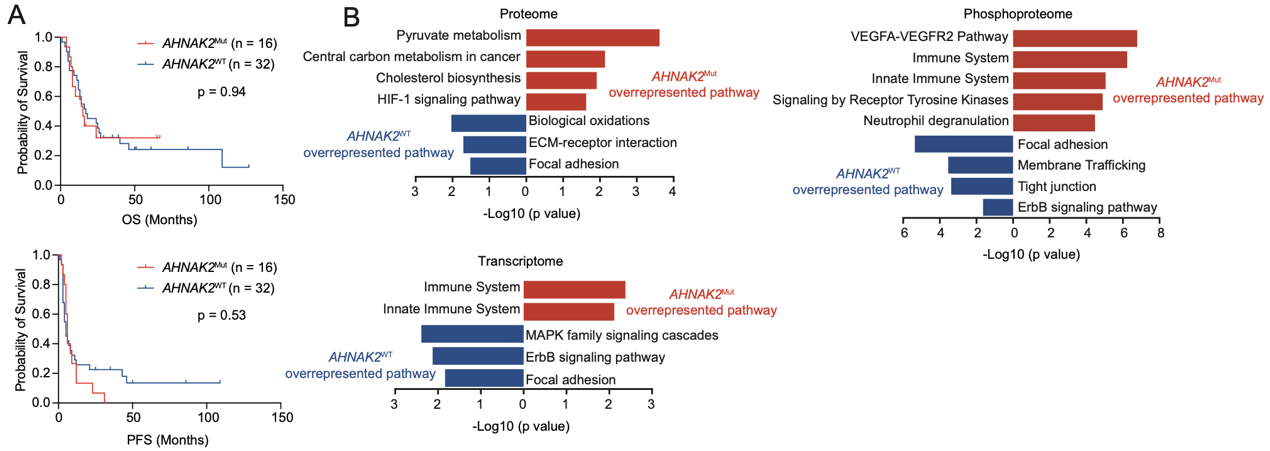


**Figure S5**

A. Kaplan-Meier curves of OS and PFS for patients with or without *ANHAK2* mutation (two-side log-rank test).

B. Pathway enrichment analysis using differentially expressed genes, proteins and phosphoproteins for patients with or without *ANHAK2* mutation.

**Section VI: About SBS6 and MLH1 expression**

Previous studies have shown that *MLH1* promoter hypermethylation or germline / somatic mismatch repair (MMR) gene mutation could lead to the loss of MMR protein expression, which may weaken DNA mismatch repair and be associated with microsatellite instability (MSI) in colorectal cancer, further related to higher TMB (*Annals of Oncology*, 2017, PMID: 27742654). In our cohort, a total of two patients (CDC_#36, CDC_#40) were detected with *MLH1* somatic missense mutations. Consistent with previous reports, tumors with *MLH1* mutations in this cohort exhibited SBS6 mutational signature, representing defective DNA MMR (**Figure S6A**). Moreover, both of them belonged to the high TMB group, with TMB values much greater than the median level (0.85 mut/Mb) of this cohort, at 21.33 and 17.72 mut/Mb, respectively (**Figure S6B**). These results preliminarily suggested that *MLH1* mutations may indeed contribute to defective MMR and higher TMB in CDC.

Unfortunately, due to the rarity of CDC and limited sample volumes, after multi-omics analysis, including genome, transcriptome, proteome, and phosphoproteome, there were no additional samples available for methylome analysis. Although methylome data were not available, multi-omics data provided an additional opportunity to explore the association between suppression of MLH1 expression with defective MMR and higher TMB in CDC.

In our cohort, MLH1 expression was detected at the proteome, transcriptome, and phosphoproteome levels. Interestingly, correlation analysis further revealed that MLH1 expression and SBS6 mutational signature exhibited significantly opposite trends at the multi-omics levels, indicating the potential association between MLH1 expression deficiency and weakened DNA MMR in CDC (**Figure S6C**). This diminished ability to repair DNA damage increased the risk of genetic errors in tumor cells, which was further associated with a higher TMB. Consistently, we found that TMB was significantly positive correlated with SBS6 mutational signature representing defective MMR in our cohort (**Figure S6D**). However, whether the suppression of MLH1 expression in CDC is caused by *MLH1* promoter hypermethylation needs to be further validated in newly collected CDC samples in the future.


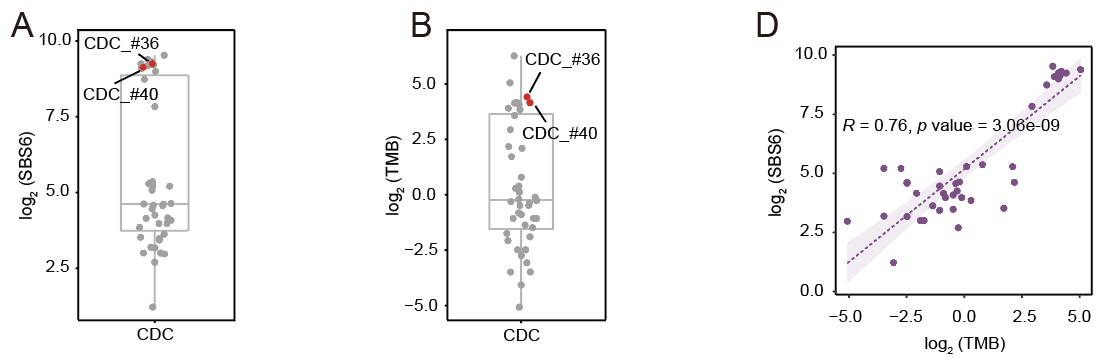


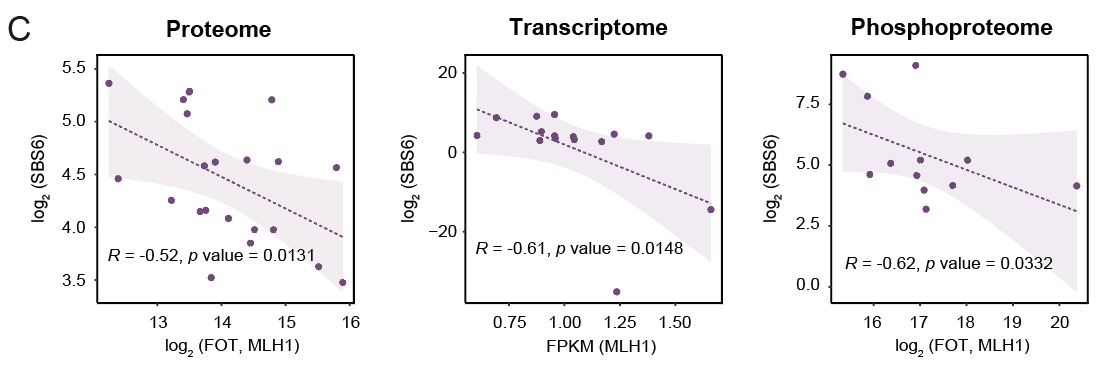


**Figure S6**

A. Boxplot showing the distribution of SBS6 mutational signature in CDC.

B. Boxplot showing the distribution of TMB in CDC.

C. SBS6 signature and MLH1 expression exhibit significantly opposite trends at the proteome (left), transcriptome (middle) and phosphoproteome (right) levels (Spearman’s correlation test).

D. TMB and SBS6 show a strong positive correlation (Spearman’s correlation test).

**Section VII: About SBS22 and** **aristolochic acid (AA) exposure**

Due to the diversity of foods and traditional Chinese medicines containing aristolochic acid (AA), it is difficult to accurately assess detailed data on patient exposure to AA (*Nature Reviews Cancer*, 2022, PMID: 35854147; *Nature*, 2024, PMID: 38693263). Thus, for the accuracy and reliability of data analysis, we inferred that the patients with SBS22 were exposed to AA.

Firstly, we calculated the mutational spectra of this cohort. The frequency of T > A transversion was the second largest in our cohort (**Figure S7A**). Then we adopted the non-negative matrix factorization (NMF) algorithms on the mutational spectra and mapping the mutational signature to the COSMIC database. SBS6, SBS22, SBS40 and SBS2 were detected in our cohort (**Figure 1f**), among which SBS22 was associated with exposure to AA. AA bonded to dA and dG residues in DNA to form aristo lactam-DNA adducts which are concentrated in the renal cortex. It is reported that aristo lactam-DNA were causally related to the initiation phase of tumorigenesis. Both dG and dA adducts block DNA replication and give rise to misincorporation of dA. When dAMP is inserted opposite the dA-AL adduct owing to misincorporation, the dA-AL is excised and replaced with dTMP leading to permanent A-to-T transversion. The repair results in a mutational pattern of marked no transcribed strand bias and the persistence of dA-AL adducts in tissues even after stopping exposure to AA for decades. SBS22 was found in cancer samples with known exposures to aristolochic acid and the pattern of mutations exhibited by the signature is consistent with that observed in experimental systems of aristolochic acid exposure (*Mutagenesis*, 2015, PMID: 26443852; *Science Translational Medicine*, 2017, PMID: 29046434). In conclusion, patients with SBS22 mutational signature were supposed to be AA exposed, which was also widely used in recent studies (*Nature Communications*, 2020, PMID: 32029730; *Cancer Cell*, 2022, PMID: 34971568; *Cell*, 2019, PMID: 31730861; *Nature Communications*, 2023, PMID: 37460463).

On this basis, we further analyzed the multi-omics characteristics of patients with SBS22 (n = 35), associated with AA exposure, compared to patients without SBS22 (n = 13). Consistent with our original manuscript, patients with SBS22 showed higher TMB level (**Figure S7B**, Wilcoxon sum-rank test p = 0.0447). As stated in the original manuscript, high expression of ribosome signaling was characteristic of the high TMB group. In this revision, single-sample gene set enrichment analysis (ssGSEA) further revealed that patients with SBS22 signature were significantly upregulated ribosome signaling (**Figure S7C**, Wilcoxon sum-rank test p = 0.0381). Overall, these results indicated that AA exposure, associated with higher cumulative genetic alterations, may be conductive to the activation of ribosome signals.


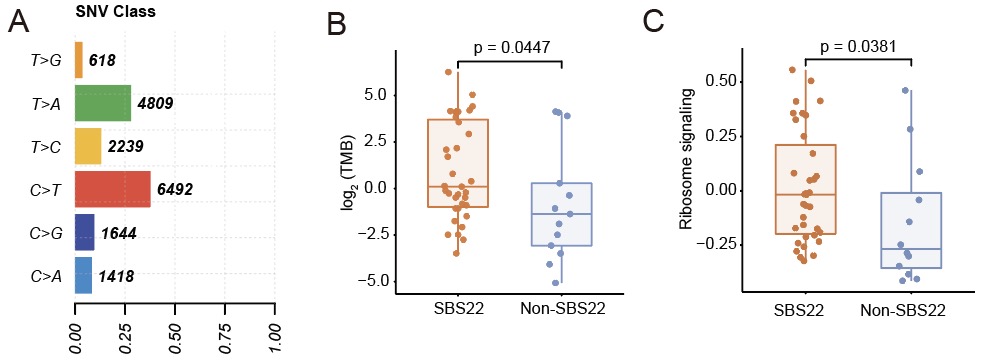


**Figure S7**

A. Mutational spectra of CDC tumors in this cohort.

B. Comparison of TMB between tumors with or without SBS22 signature (Wilcoxon sum-rank test).

C. Comparison of pathway scores for the ribosome signaling in tumors with or without SBS22 signature (Wilcoxon sum-rank test).

**Section VIII: About the proteins** **and phosphosites with a missing rate of less than 75% of samples**

It was well-established that cancer showed distinct intra-tumoral and inter-tumoral heterogeneity. A considerable portion of proteins and phospho-proteins were only expressed in a portion of tumor samples, which would lead to excessive variation of them. To improve the generality of our analysis results, proteins and phosphosites with medium to high identification frequency were included for the downstream analysis.

Secondly, to further investigate whether different thresholds showed significantly diverse biological impacts, we performed over-representation analysis (ORA) based on the differentially expressed proteins and phospho-proteins (DEPs) with different thresholds. Specifically, the DEPs (FC > 2, Wilcox test’s adjusted p value < 0.05) were detected in more than 25%, more than 45%, more than 65% and more than 85% of samples, respectively. The results showed that DEPs based on the four thresholds represented similar enriched pathways. Notably, at proteomic level, regardless of the threshold, ribosome biogenesis related pathways were the top pathways enriched in CDC tumors (**Figure S8A**), whereas metabolic processes and oxidative phosphorylation pathway were enriched in NATs (**Figure S8B**). In addition, at phospho-proteomic level, ribosome biogenesis and assembly pathways, as well as cell migration were activated in CDC tumors (**Figure S8C**), regardless of the threshold. In NATs, pathway enrichment results also showed high consistency (**Figure S8D**). Overall, these results indicated that using proteins and phosphosites with a missing rate of < 75% of samples for analysis was robust.

Thirdly, the analysis focusing on the proteins with a missing rate of less than 75% of samples has been applied in the previously published studies. For example, the breast cancer study published in *Cell* (2020, PMID: 33212010) have demonstrated that proteins were required to have a missing rate of < 75% of samples in order to be included in the proteome dataset. In the study of lung squamous cell carcinoma from CPTAC published in *Cell* (2021, PMID: 34358469), proteins missing in > 75% of samples were excluded from downstream analysis. A ccRCC cohort undergoing Sunitinib treatment (*Nature Communications,* 2023, PMID:37460463) and a diffuse-type gastric cancer cohort (*Nature Communications*, 2018, PMID: 29739932) also used a missing rate of 75% as cutoff for the downstream analysis. These results showed that our data filtering method was reliable.

In addition, there were 7,396 proteins and 8,738 phosphosites detected in at least 25% of the samples in our cohort. After data filtering, we identified 5,439 proteins per sample, ranging from 3,613 to 6,544. More than 93% of samples had identified protein numbers over 4500, and only two samples had identified protein numbers less than 4000 (3,613 and 3,961, respectively). Moreover, after data filtering, about 80% of samples had identified phosphosite numbers over 4000. These results further indicated that the substantial portion of the data was observed rather than inferred.


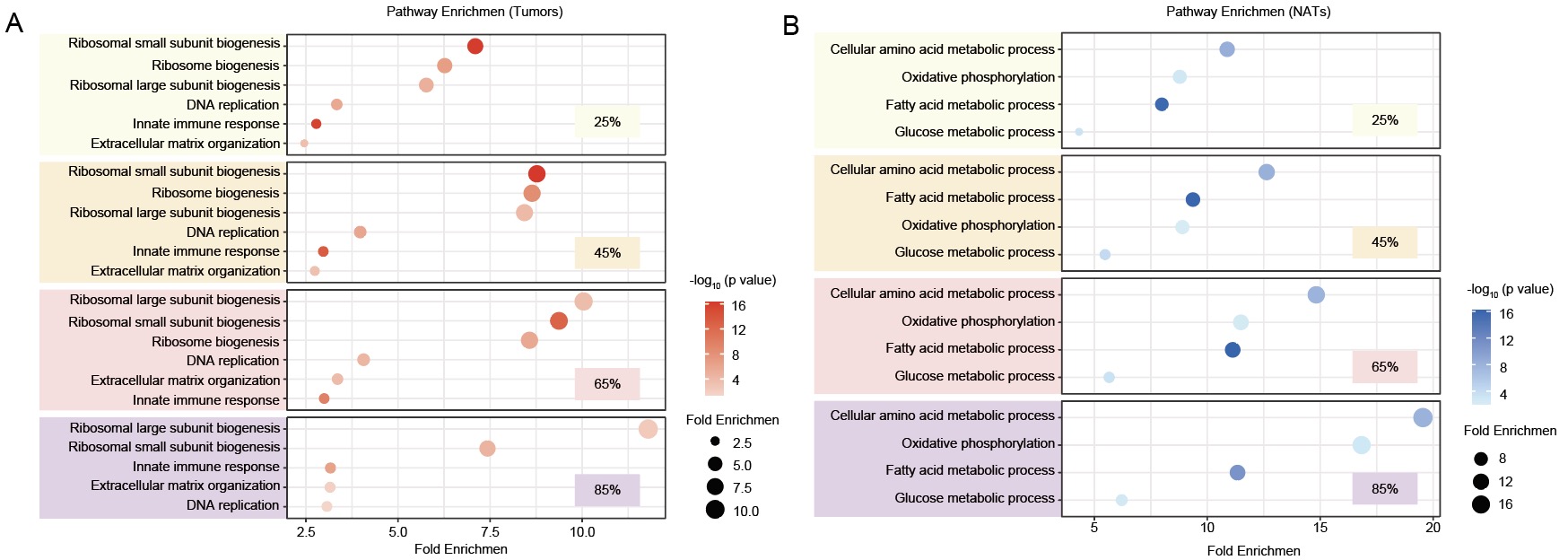


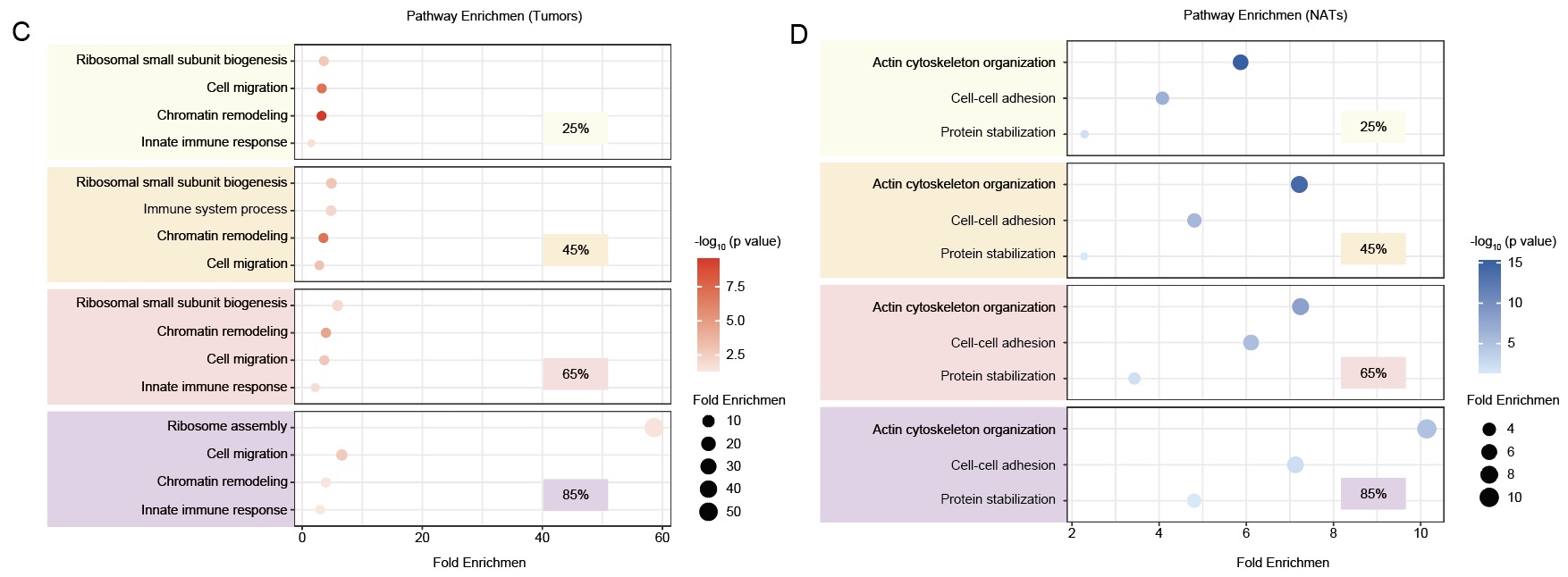


**Figure S8**

A, B. Over-representation analysis (ORA) of differentially expressed proteins from four threshold (25%, 45%, 65% and 85%).

C, D. ORA of differentially expressed phospho-proteins from four threshold (25%, 45%, 65% and 85%).

**Section IX: About the** **possible reasons of the imperfect correlations of mRNA-protein**

**Possible reason 1: The temporal and spatial differences in mRNA and protein synthesis result in a low RNA-protein correlation observed at a certain time.** According to the central dogma of molecular biology, DNA carrying genetic information is first transcribed into mRNA. Then, mRNA moves from the nucleus to the cytoplasm, where it binds to the ribosome and is translated into the mature protein to achieve the transmission of genetic information and maintain the physiological functions of the body. It takes time for a gene to go from being transcribed into mRNA to being translated into protein (**Figure S9A**). However, when we observed the correlation between RNA and protein, we choose the same time point, which may result in mRNA synthesis not being transported to the cytoplasm for further translation into protein. Due to the temporal and spatial differences in mRNA and protein synthesis, the mRNA-protein correlation obtained by detecting mRNA and protein levels at a certain time may be lower than their actual correlation (*Nature Reviews Genetics*, 2020, PMID: 32709985).

**Possible reason 2: The correlation between protein and mRNA half-lives is low.** The second reason for the low correlation between mRNA and protein may be that mRNA and protein have different half-lives. Schwanhäusser et al. reported that the median half-life of proteins is 46h, while the median half-life of mRNA is 9h (**Figure S9B**) (*Nature*, 2011, PMID: 21593866). In addition, the dynamic range of half-lives of proteins spans more than 3.5 orders of magnitude, while the dynamic range of half-lives of mRNAs spans 1.5 orders of magnitude (**Figure S9B**) (*Nature*, 2011, PMID: 21593866). Notably, Schwanhäusser et al. found no correlation between protein and mRNA half-lives (R^2^ = 0.02) (**Figure S9C**) (*Nature*, 2011, PMID: 21593866). Thus, two genes with the same RNA half-life may have distinct protein half-lives, which reduces the RNA-protein correlation observed. This factor further contributes to the low RNA-protein correlation.

**Possible reason 3: Post-transcriptional and post-translational regulation.** In addition to the reasons described above, post-transcriptional and post-translational regulation further affect the mRNA and protein correlation. From RNA to protein is a complex and delicate process, and regulatory activities such as post-transcriptional regulation and post-translational regulation are likely to occur after mRNA formation. Post-transcriptional regulation (such as mRNA splicing, etc.), the bridge between mRNA and protein, which directly determines the mRNA and protein levels. Post-translational regulation (such as glycosylation, ubiquitination, etc.) can lead to protein isomerization and quantity change. This factor further affects the mRNA and protein correlation.

Thus, measuring mRNA and protein levels is an integral part of our effort to understand how the genome impacts phenotype. The hierarchy of the gene expression pathway forms the basis of the largely correct assumption that protein abundance should scale with mRNA abundance, both across and within genes. However, due to (1) the temporal and spatial differences in mRNA and protein synthesis, (2) the low correlation between mRNA and protein half-lives, and (3) post-transcriptional and post-translational regulation, the observed mRNA-protein correlation is often low.

**
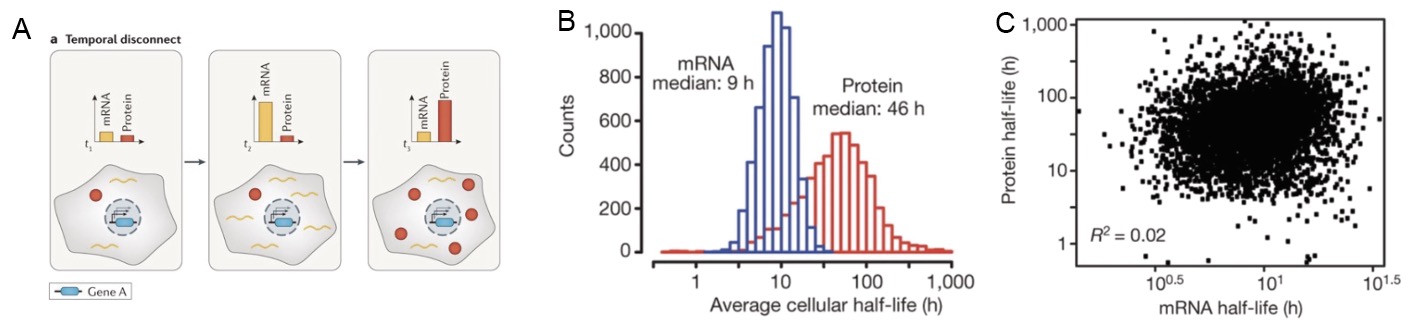
**

**Figure S9**

A. Temporal sequence of RNA and corresponding protein synthesis. mRNA levels increase transiently at the second time point, followed by delayed increases in protein levels. (*Nature Reviews Genetics*, 2020, PMID: 32709985)

B. Histograms of mRNA (blue) and protein (protein) half-lives. (*Nature*, 2011, PMID: 21593866)

C. Correlation between mRNA and protein half-lives. (*Nature*, 2011, PMID: 21593866)
