# Supplementary figures and images for: Proteogenomic Analysis Identifies Clinically Relevant Subgroups of Collecting Duct Carcinoma
Source: Research (Wash D C). 2025 Sep 3;8:0859. doi: 10.34133/research.0859 (PMC12408255; doi:10.34133/research.0859)

# Figure S1

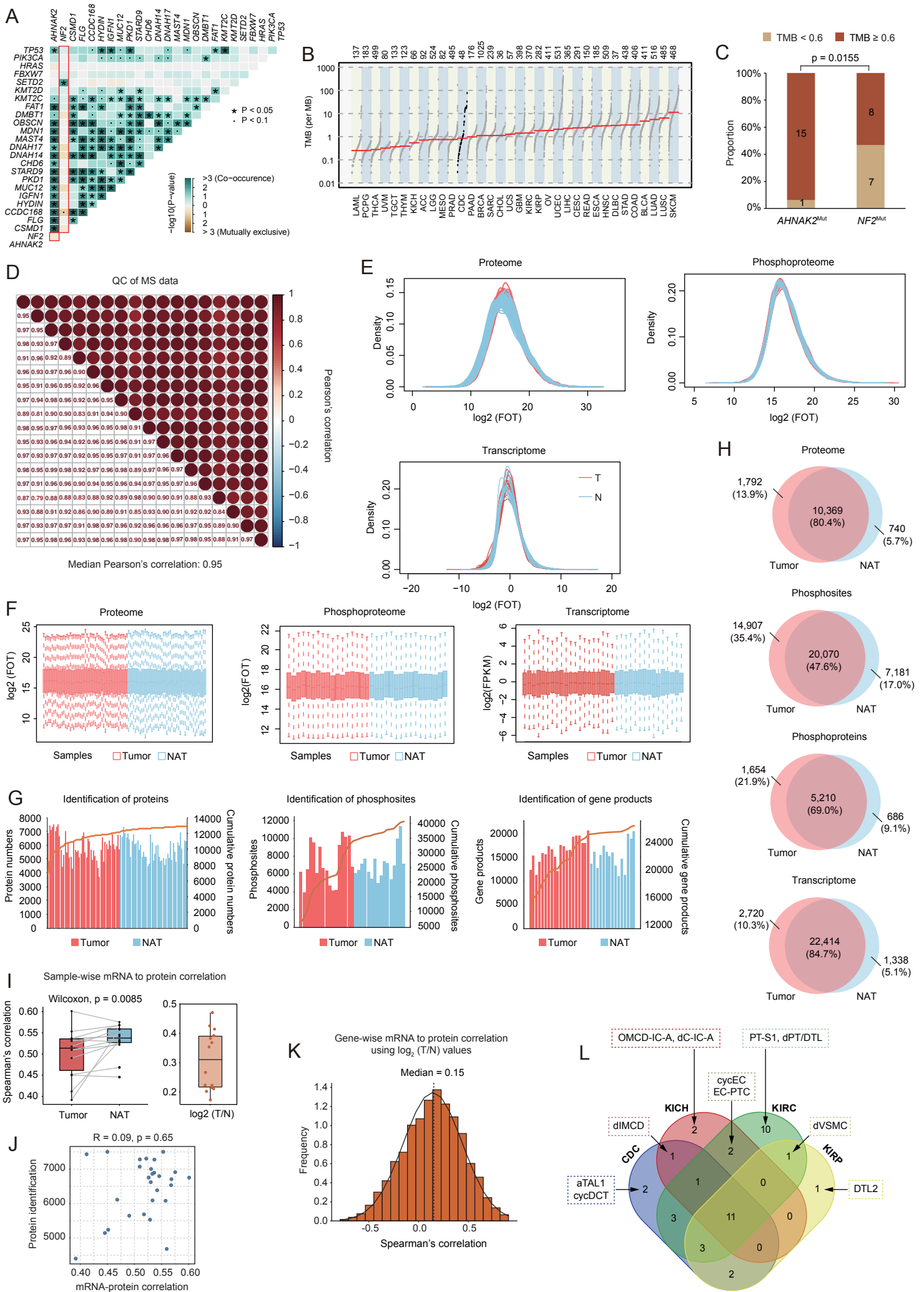

Supplement: Supplementary 1 — Figs. S1 to S8 Data S1 to S6 [file research.0859.f1.zip › Figure S1.pdf]

# Figure S2

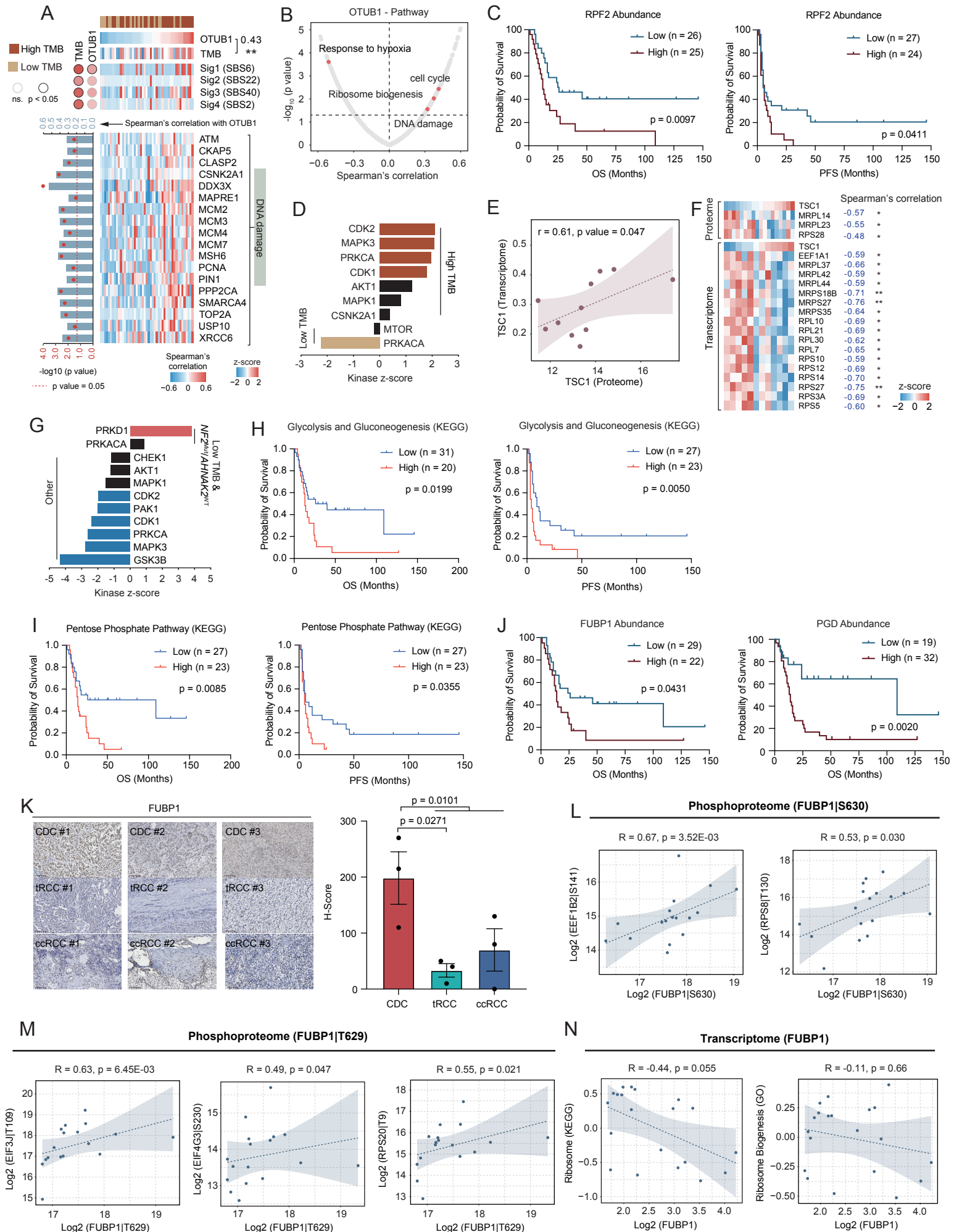

Supplement: Supplementary 1 — Figs. S1 to S8 Data S1 to S6 [file research.0859.f1.zip › Figure S2-1.pdf]

# Figure S3

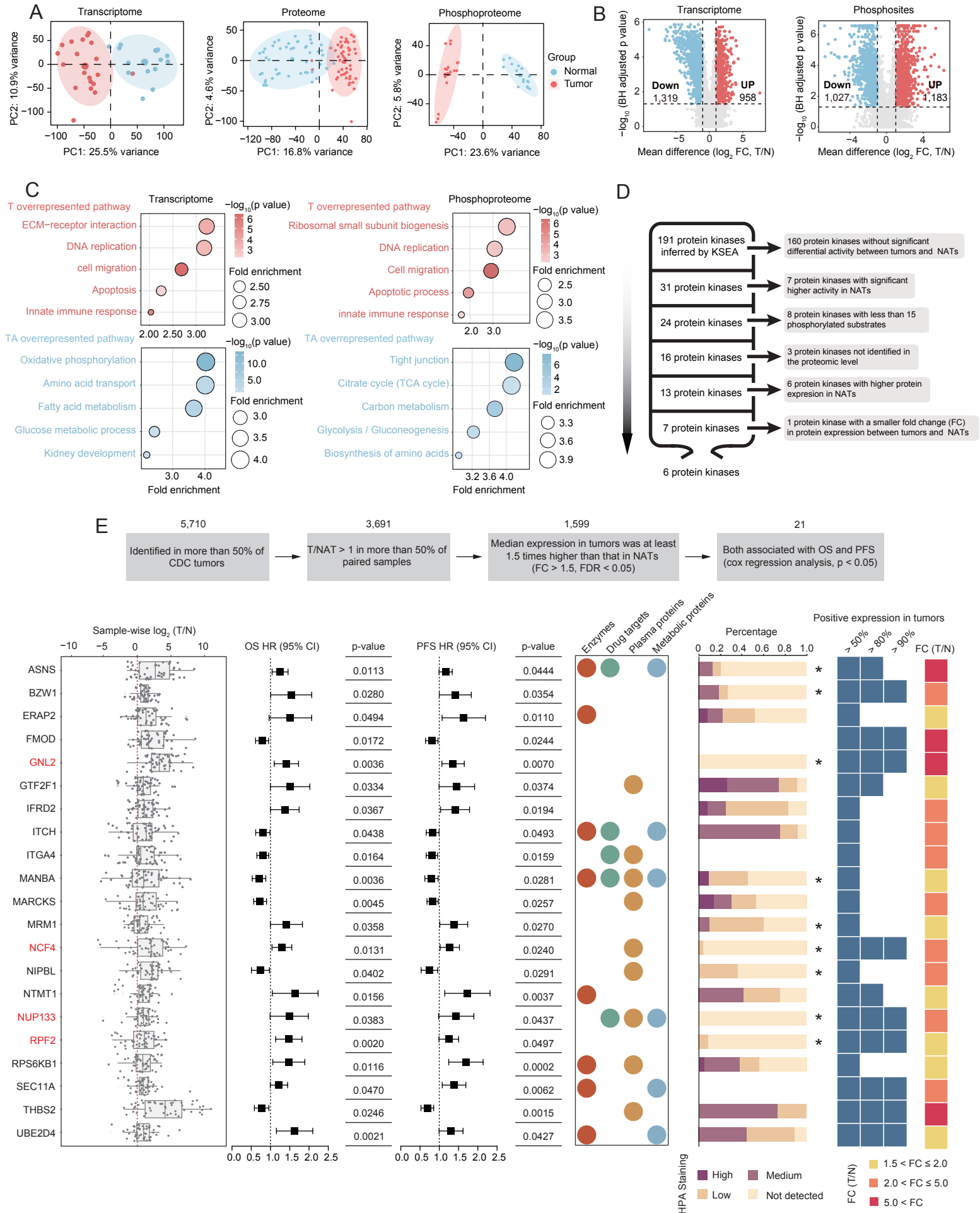

Supplement: Supplementary 1 — Figs. S1 to S8 Data S1 to S6 [file research.0859.f1.zip › Figure S3.pdf]

# Figure S4

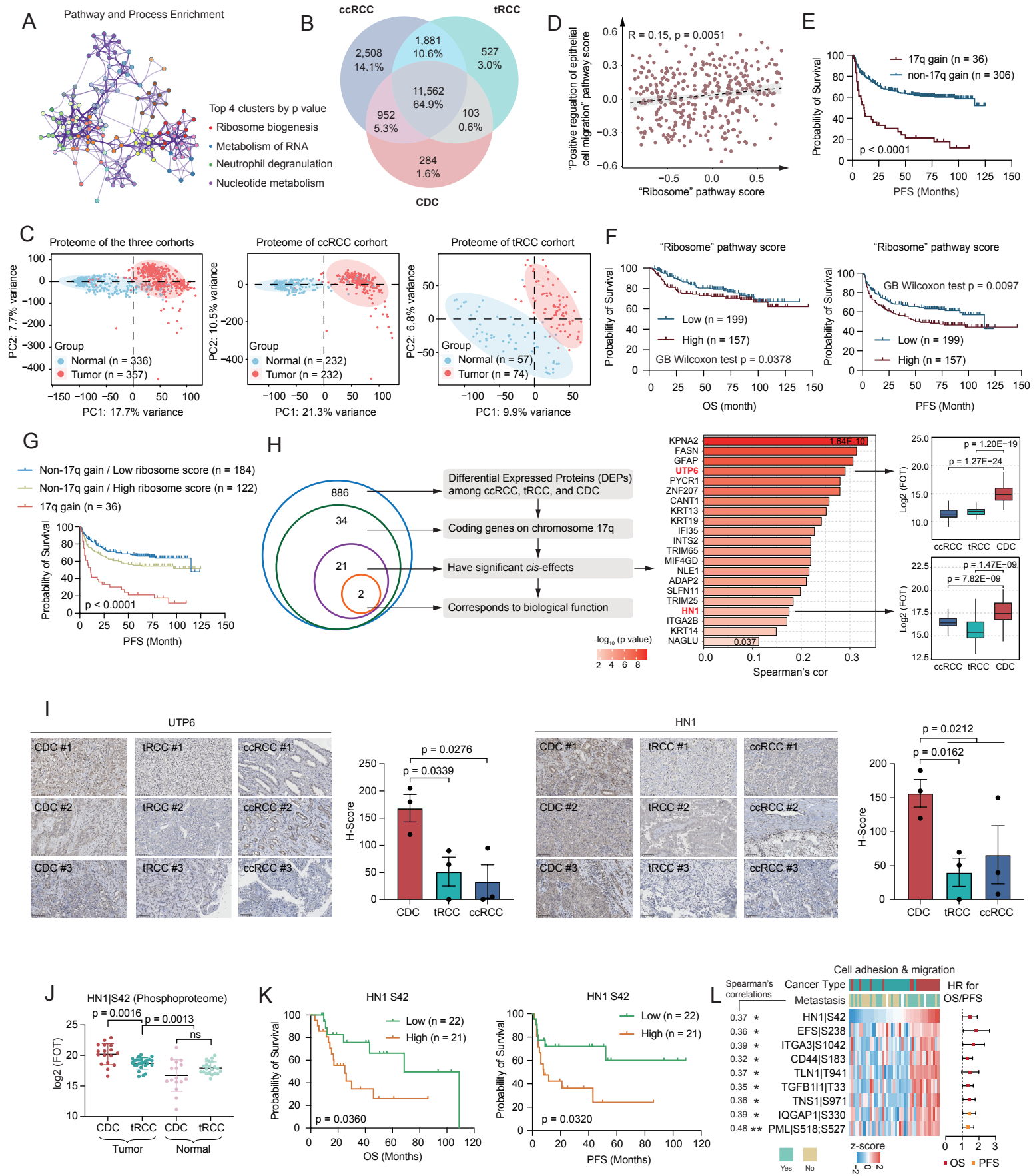

Supplement: Supplementary 1 — Figs. S1 to S8 Data S1 to S6 [file research.0859.f1.zip › Figure S4-1.pdf]

# Figure S5

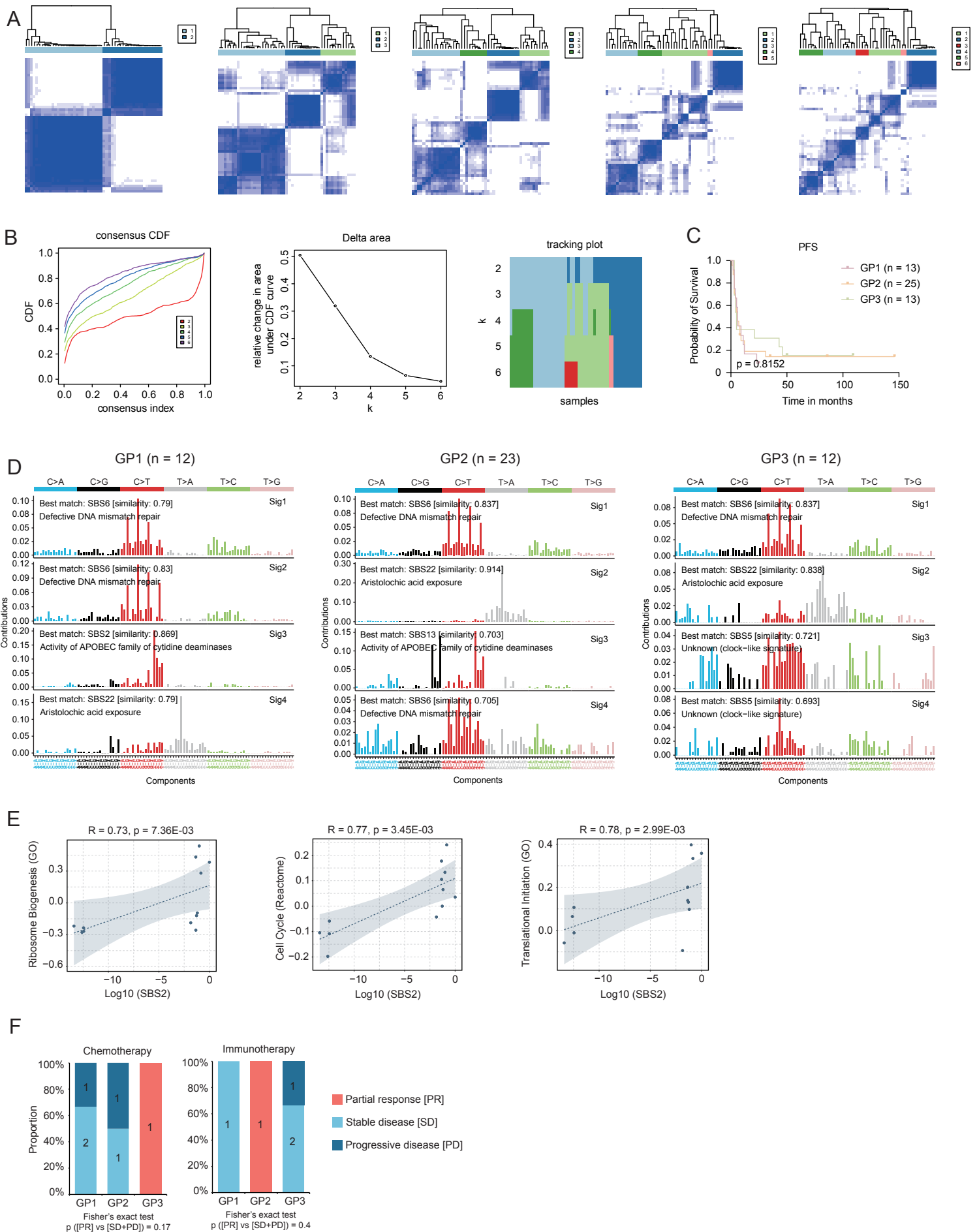

Supplement: Supplementary 1 — Figs. S1 to S8 Data S1 to S6 [file research.0859.f1.zip › Figure S5.pdf]

# Figure S6

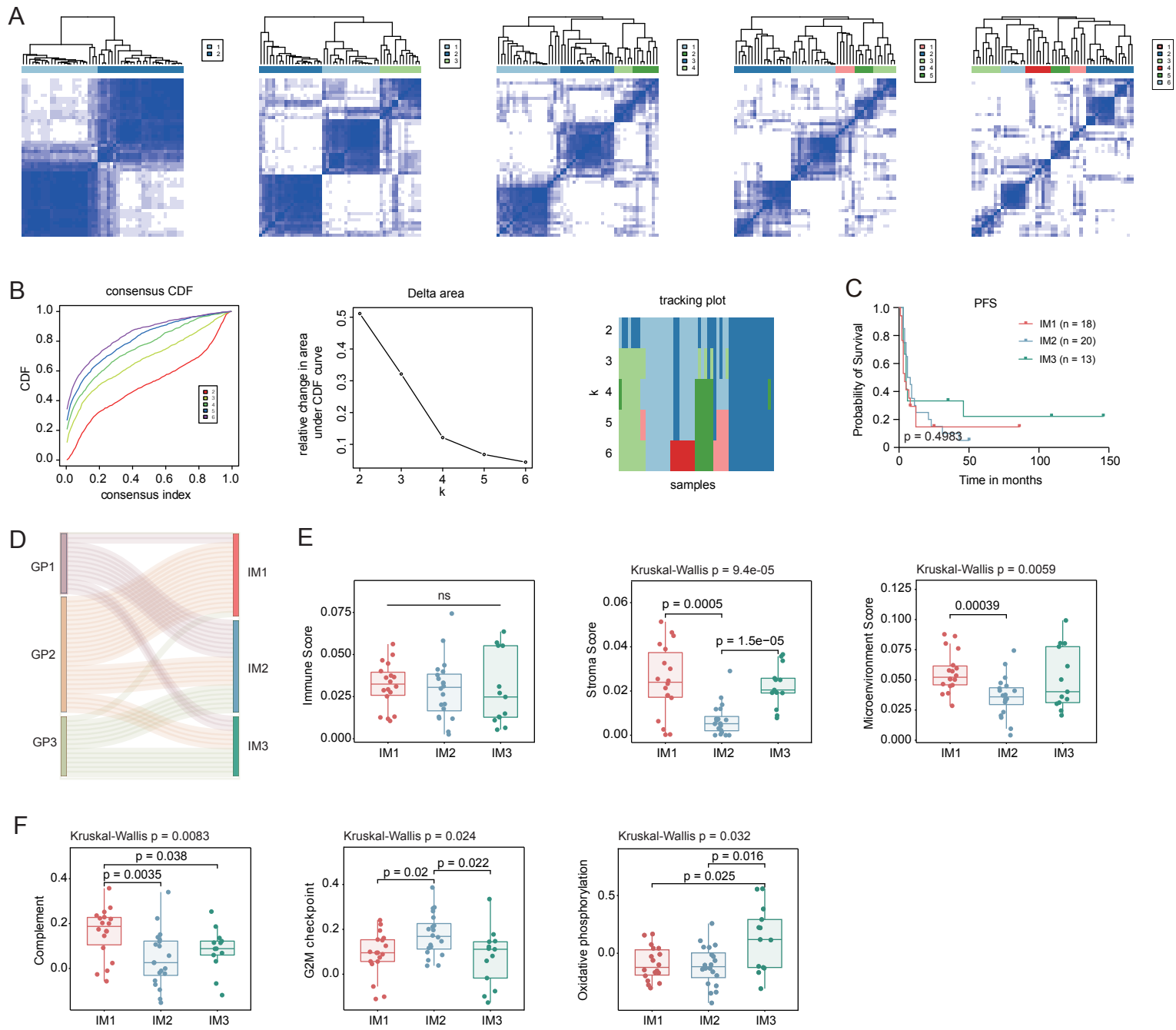

Supplement: Supplementary 1 — Figs. S1 to S8 Data S1 to S6 [file research.0859.f1.zip › Figure S6.pdf]

# Figure S7

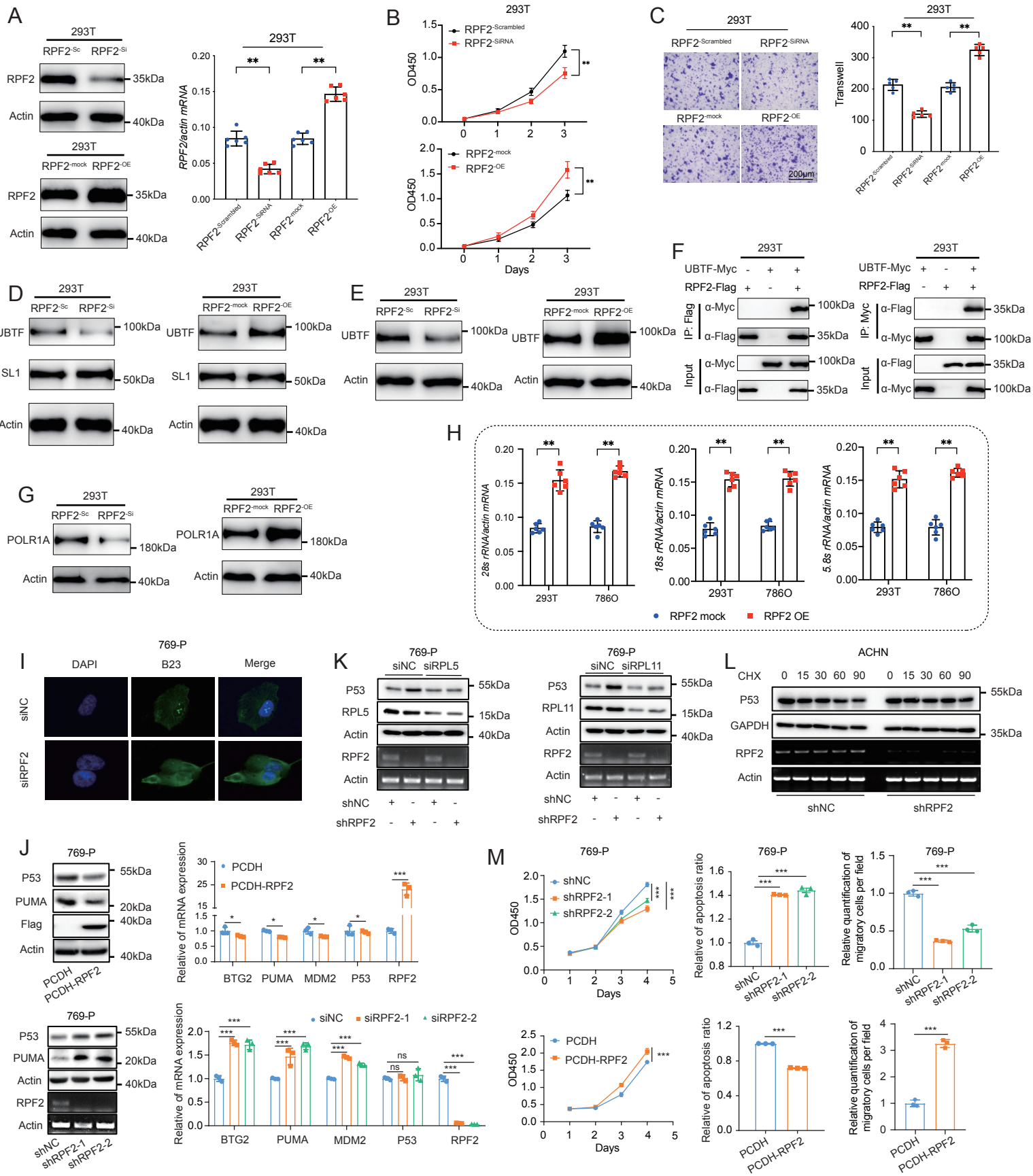

Supplement: Supplementary 1 — Figs. S1 to S8 Data S1 to S6 [file research.0859.f1.zip › Figure S7.pdf]

Figure S8

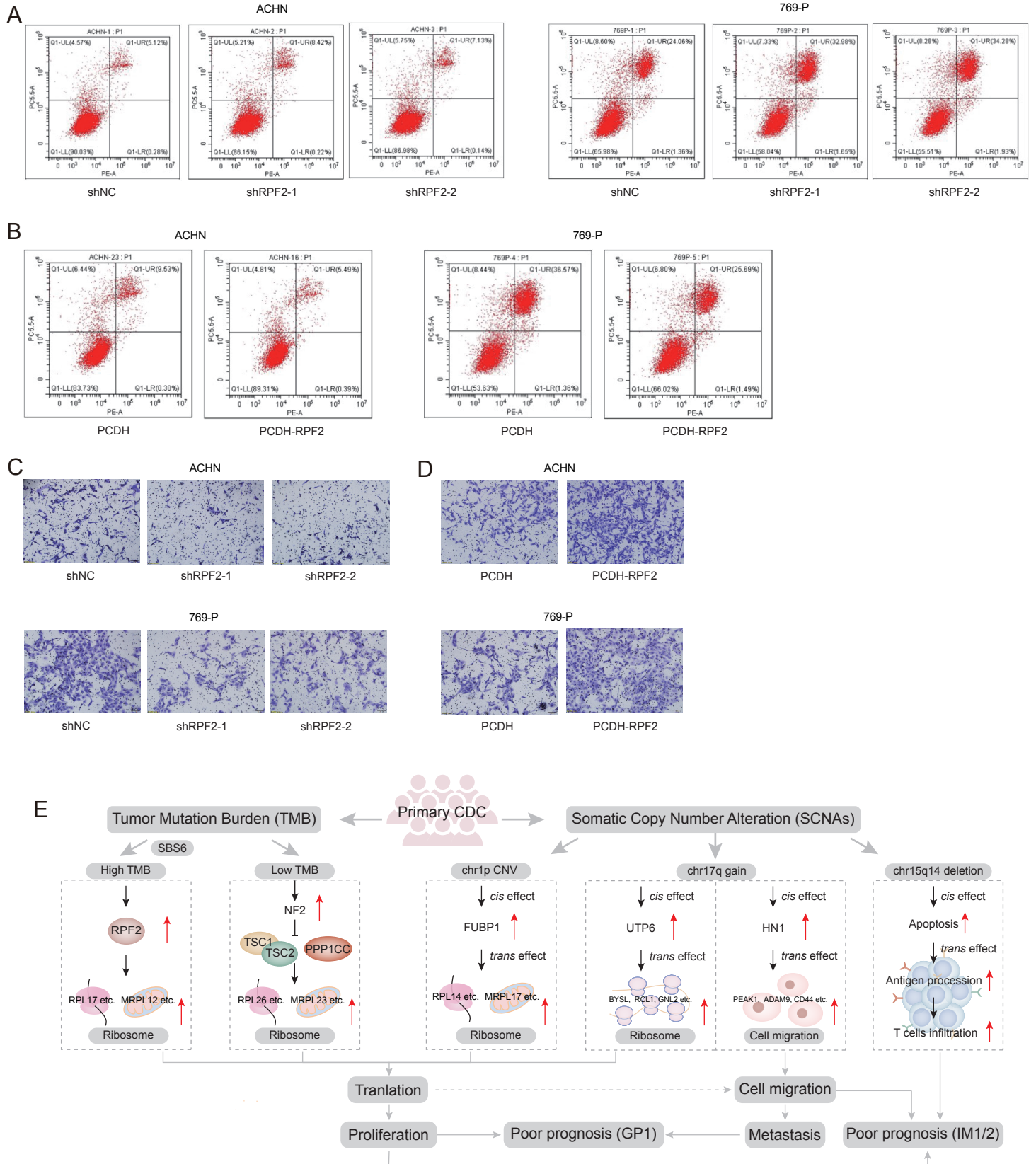

Supplement: Supplementary 1 — Figs. S1 to S8 Data S1 to S6 [file research.0859.f1.zip › Figure S8.pdf]
